# Plant pectin acetylesterase structure and function: new insights from bioinformatic analysis

**DOI:** 10.1186/s12864-017-3833-0

**Published:** 2017-06-08

**Authors:** Florian Philippe, Jérôme Pelloux, Catherine Rayon

**Affiliations:** EA3900-BIOPI, Biologie des Plantes et Innovation, Université de Picardie Jules Verne, 80039 Amiens, France

**Keywords:** Pectin acetylesterase, *Arabidopsis thaliana*, Phylogenetic tree, Conserved motifs, 3D homology

## Abstract

**Background:**

Pectins are plant cell wall polysaccharides that can be acetylated on C2 and/or C3 of galacturonic acid residues. The degree of acetylation of pectin can be modulated by pectin acetylesterase (EC 3.1.1.6, PAE). The function and structure of plant PAEs remain poorly understood and the role of the fine-tuning of pectin acetylation on cell wall properties has not yet been elucidated.

**Results:**

In the present study, a bioinformatic approach was used on 72 plant PAEs from 16 species among 611 plant PAEs available in plant genomic databases. An overview of plant PAE proteins, particularly *Arabidopsis thaliana* PAEs, based on phylogeny analysis, protein motif identification and modeled 3D structure is presented. A phylogenetic tree analysis using protein sequences clustered the plant PAEs into five clades. AtPAEs clustered in four clades in the plant kingdom PAE tree while they formed three clades when a phylogenetic tree was performed only on Arabidopsis proteins, due to isoform AtPAE9. Primitive plants that display a smaller number of PAEs clustered into two clades, while in higher plants, the presence of multiple members of PAE genes indicated a diversification of AtPAEs. 3D homology modeling of AtPAE8 from clade 2 with a human Notum protein showed an α/β hydrolase structure with the hallmark Ser-His-Asp of the active site. A 3D model of AtPAE4 from clade 1 and AtPAE10 from clade 3 showed a similar shape suggesting that the diversification of AtPAEs is unlikely to arise from the shape of the protein. Primary structure prediction analysis of AtPAEs showed a specific motif characteristic of each clade and identified one major group of AtPAEs with a signal peptide and one group without a signal peptide. A multiple sequence alignment of the putative plant PAEs revealed consensus sequences with important putative catalytic residues: Ser, Asp, His and a pectin binding site. Data mining of gene expression profiles of *AtPAE* revealed that genes from clade 2 including *AtPAE7*, *AtPAE8* and *AtPAE11*, which are duplicated genes, are highly expressed during plant growth and development while AtPAEs without a signal peptide, including *AtPAE2* and *AtPAE4*, are more regulated in response to plant environmental conditions.

**Conclusion:**

Bioinformatic analysis of plant, and particularly Arabidopsis, AtPAEs provides novel insights, including new motifs that could play a role in pectin binding and catalytic sites. The diversification of AtPAEs is likely to be related to neofunctionalization of some *AtPAE* genes.

**Electronic supplementary material:**

The online version of this article (doi:10.1186/s12864-017-3833-0) contains supplementary material, which is available to authorized users.

## Background

The plant primary cell wall is a dynamic structure consisting mostly of polysaccharides, which are organized in a highly cross-linked polymer network [1, 2]. Pectins are important structural polysaccharides in the primary cell wall, representing up to one third of its dry mass. These polysaccharides are rich in galacturonic acids (GalA) and comprise different structural domains: homogalacturonan (HG), xylogalacturonan (XGA), rhamnogalacturonan-I (RG-I) and minor amounts of rhamnogalacturonan-II (RG-II) [3, 4]. The ratio between these pectic polysaccharides depends on the species, tissues and developmental stages [5]. However, HG is the most abundant pectin polymer and is composed of α-1,4-linked-D-galacturonic acid units [3]. RG-I is the second most abundant pectic polysaccharide and consists of a repeating disaccharide of α-1,4 GalA-α-1,2-L-rhamnose, which can be substituted with side chains of galactans and arabinans [6]. The GalA residues can be acetylated at positions O-2 or O-3 in both HG and RG-I [6–8]. The degree of acetylation can be regulated by pectin acetylesterase (E.C. 3.1.1.6; PAE) which cleave the acetylester bond from pectin [5, 9]. Plant PAEs belong to family CE13 of carbohydrate esterases in the CAZy database [10]. PAEs have also been identified in bacteria and fungi where they belong to family CE12 [10–15]. CE12 and CE13 are classified as SGNH family proteins (Pfam accession number: CL0264). The 12 families in this superfamily are characterized by four conserved residues: S, G, N and H. There is also an aspartic residue that appears to be involved in the catalytic site [16, 17]. To date, few studies have reported the biochemical properties and physiological function of PAE [5, 9, 18–21]. Furthermore, analyses of the evolution, function and structure of the *PAE* gene family in plants are very scarce [21, 22]. An evolutionary history analysis of 16 pectin-related gene families from 10 diverse plant genomes showed that *Physcomitrella patens*, a lower plant, has fewer pectin-related gene family members compared to Arabidopsis [22]. The Physcomitrella genome was found to contain only one *PAE* gene while in higher plants multiple members were observed, suggesting a minimum of one ancestral PAE in the earliest land plant [22]. Another phylogenetic tree study based on PAE protein sequences from up to 35 plant species, including Arabidopsis, proposed a putative minimal set of 4 distinct PAEs in the plant for its function [21]. In addition, these authors identified three specific grass PAE clades, one of which showed a low level of conservation with the other clades of the tree, consistent with the hypothesis of a neofunctionalization of PAEs in grass.

The sequencing of several plant genomes has significantly promoted the identification and characterization of plant genes. Several portals and databases of genes, protein sequences and functions, including Phytozome, Uniprot, PlantCAZyme, and eFP Browser, can be used to investigate the *PAE* gene family in the plant kingdom [23–26]*.* One way to have a better understanding of the structure and function of a protein family, including Arabidopsis, is to use comparative genomics and phylogenetic approaches [21].

In our study, a comprehensive analysis of plant PAEs, and particularly that of Arabidopsis, was undertaken using bioinformatic approaches. A phylogenetic tree using Arabidopsis PAE protein sequences was generated. Three clades of Arabidopsis PAEs were identified. The 3D homology of one Arabidopsis PAE from each cluster was modeled to investigate the diverging branches. The 3D homology models could not explain the diversification of PAEs but could identify a common structural location of the putative catalytic triad. Based on Arabidopsis PAE primary protein sequences, conserved motifs of AtPAE as well as specific motifs for each clade were further characterized. Their putative role was analyzed. In addition, a comprehensive analysis of the gene expression profiles of Arabidopsis *PAE* genes in different tissues, at different stages of development and in response to several stresses was performed. It appears that the expression of AtPAEs from clade 2 is more specifically regulated during plant growth and development. In contrast to clade 2, the expression of *AtPAE4* from clade 3, whose protein does not contain a signal peptide, is more regulated in response to environmental conditions. This allowed us to hypothesize that the differences in primary sequences and expression could drive the functional diversification of AtPAEs. This led to the identification of a number of PAE isoforms of interest, including AtPAE which should be further studied to understand the contribution of the fine-tuning of pectin acetylation to plant development.

## Results

### Phylogenetic tree of plant PAE proteins

In order to study plant PAEs in a broader evolutionary context, a phylogenetic tree was generated from 72 plant PAEs using the full-length sequence of the proteins without their signal peptides. The sequences of putative PAEs were retrieved from the Phytozome, PlantCAZyme and Uniprot databases [24–26]. The 611 putative plant PAEs available in these databases were from 37 diverse genomes. Of these 611, 72 plant PAEs from 4 clades (Moss, Spike moss, Dicots, Monocots) and 16 species, including Arabidopsis, maize, rice, Physcomitrella, Selaginella, Sorghum, Brachypodium, flax, poplar, Medicago, litchi, lettuce, castor bean and cacao tree, were selected (Additional files 1 and 2). These data were chosen using different criteria, including extensive sequence annotation of the PAE protein of some genomes, availability of full-length protein sequence, model species as well as relevant literature on plant PAE gene function [5, 20–22].

In all species, PAEs are encoded by a multigene family, except for Physcomitrella, in which only one PAE has been annotated according to the Phytozome database. The number of *PAE* genes in lower plants appears lesser (1–7) than in higher plants (7–27). The average number of PAEs in higher plants (between 10 and 11) is independent of the size of the genome (Additional file 1). The highest number of PAE isoforms was mostly found in dicots, including apple (27), soybean (23), oil seed rape (22), and yellow monkey flower (20). However, 24 putative PAEs were annotated in Switchgrass, a monocot, while 11 and 12 putative isoforms were annotated in Sorghum and Brachypodium, respectively (Additional file 1). We cannot exclude that the low numbers of *PAE* genes in some genomes could be related to the incomplete sequenced genome or the incomplete annotation sequence of some genomes, including cucumber, castor bean, lettuce and flax, which might be an issue.

**Fig. 1:**
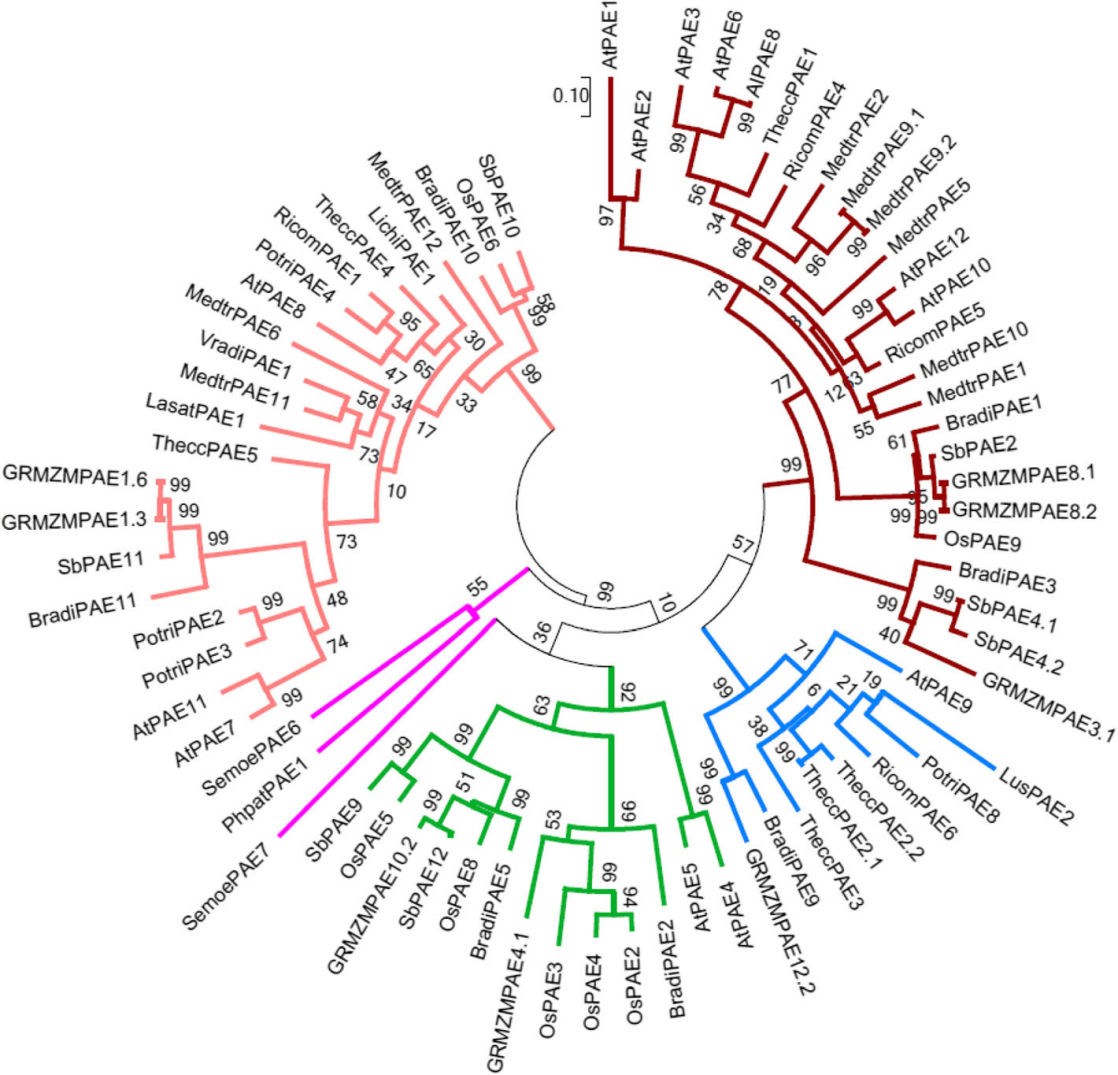
Phylogenetic tree of 72 plant PAE proteins. The evolutionary history was inferred by using the Maximum Likelihood method based on the JTT matrix-based model [71]. The tree with the highest log likelihood (−22,303.4018) is shown. Initial tree(s) for the heuristic search were obtained automatically by applying Neighbor-Join and BioNJ algorithms to a matrix of pairwise distances estimated using a JTT model, and then selecting the topology with superior log likelihood value. The tree is drawn to scale, with branch lengths measured in the number of substitutions per site. The analysis involved 72 amino acid sequences. All positions with less than 95% site coverage were eliminated. That is, fewer than 5% alignment gaps, missing data, and ambiguous bases were allowed at any position. There were a total of 341 positions in the final dataset. Evolutionary analyses were conducted in MEGA7 [27]. Each major clade is identified with a specific color. Clade 1 is in *purple*, clade 2 in *green*, clade 3 in *blue*, clade 4 in *dark red* and clade 5 in *pink*

The phylogenetic analysis of the 72 selected PAE sequences was carried out with MEGA 7 using the maximum likelihood method following multiple sequence alignment with Clustal W [27]. We constructed a multiple sequence alignment of the 72 selected AtPAEs using the BLOSUM60 score matrix. A 60% threshold was used due to the potential sequence diversity between plant species. In this tree, five clades representing PAEs from different plant species were identified (Fig. 1). The number of PAE proteins in clades 1, 2, 3, 4, and 5 was 2, 14, 9, 25, and 22, respectively. One PAE from Moss and one from Spike moss formed one cluster, clade 1, suggesting that lower plant PAEs have lower sequence similarity than higher plant PAEs. However, a spike moss PAE, SemoePAE7 clustered with some grass PAEs and two Arabidopsis PAEs, AtPAE4 and AtPAE5 to form clade 2. This indicates a potential evolution of some PAE from lower plants. Arabidopsis PAE proteins (AtPAEs) appeared in the four other clades. Clade 2 included two AtPAEs, AtPAE4 and AtPAE5, supporting the idea that these two proteins, which are more closely related to PAEs from lower plants, could act on a broad spectrum of pectic polysaccharides. Clade 3 contained one AtPAE, AtPAE9. Clade 4 is composed of six AtPAEs, AtPAE1, AtPAE2, AtPAE3, AtPAE6, AtPAE10 and AtPAE12 and Clade 5 displays three AtPAEs, AtPAE7, AtPAE8 and AtPAE11.

A phylogenetic tree of some grass PAEs including maize, rice, sorghum along with AtPAEs was constructed in a similar way. Four clades were identified (Additional file 3a). AtPAE proteins were present in the four clades suggesting some conservation between grass PAE and AtPAE proteins.

### Phylogenetic tree of Arabidopsis PAE proteins

To try and explain the sequence-structure-function relationship of plant PAEs, we focused on the classification of PAEs from Arabidopsis.

The Arabidopsis *PAE* gene family comprises 12 members that are present on each of the chromosomes (Additional file 4). A number of genes, including *AtPAE7* (*At4g19410*) and *AtPAE8* (*At4g19420*) or *AtPAE4* (*At3g09405*) and *AtPAE5* (*At3g09410*) are likely to correspond to tandem duplication. The pairwise protein sequence identity for the tandem duplicated genes was higher for *AtPAE4*/*AtPAE5* (80%) than for *AtPAE7*/*AtPAE8* (57%), (Additional file 5). In most cases, the *AtPAE* gene structure was characterized by a large number of introns (10–12) and a coding region comprising 11–13 exons. This high number of exons and introns appears to be rather specific to the *AtPAE* gene family when compared to other pectin-related gene families, such as *PMEs* and *PGs*.

**Fig. 2:**
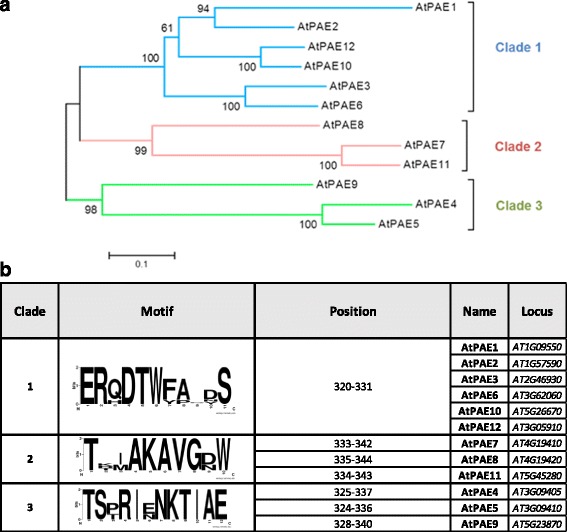
Phylogenetic analysis and specific conserved sequence motifs in the *Arabidopsis thaliana* PAE protein sequences. **a** The tree shows three distinct groups of Arabidopsis PAEs. The tree was generated by neighbor-joining distance analysis of PAE protein sequences using the Muscle program in Mega 7.0 software [27]. Protein sequence alignment was achieved by BLOSUM80. The sequences used are full-length proteins without their signal peptide. **b** The sequence logo was made using WebLogo 3 [34]. AtPAEs are clustered in three groups according to the motif displayed by Logoplot. The position of the identified motifs is indicated

We constructed a phylogenetic tree for the 12 AtPAEs, in a similar way to the one obtained with the plant PAEs (Fig. 2a). A BLOSUM80 score matrix was used since AtPAEs are homolog proteins. The twelve Arabidopsis PAEs were grouped into three clades, instead of the four clades observed in the plant kingdom tree. As observed in the phylogenetic tree of the land plant (Fig. 1), AtPAE1, AtPAE2, AtPAE3, AtPAE6, AtPAE10 and AtPAE12 clustered in a similar way and formed clade 1. Clade 2 included AtPAE7, AtPAE8 and AtPAE11, while AtPAE4, AtPAE5 and AtPAE9 clustered in clade 3. Pairwise sequence identity was in the range of 55–88%, 54–83% and 46–79% for clades 1, 2 and 3, respectively (Additional file 5). This indicates that AtPAEs from clade 1 are more conserved across this clade. In addition, AtPAE proteins from clade 1 had 44–55% and 39–49% amino acid identity with AtPAEs from clades 2 and 3, respectively. AtPAEs from clade 2 had 42–48% amino acid identity with AtPAEs from clade 3. The percentage of similarity of amino acids for a given pair of AtPAEs was 20–30% higher than their pairwise protein sequence identity (Additional file 5).

### Structural similarity of AtPAE to human Notum

We hypothesized that the diversification of AtPAEs could arise from differences in the structure among the three clades of AtPAE.

Based on the classification of Arabidopsis PAE (Fig. 2a), we generated 3D homology models of PAE4 (clade 3), PAE8 (clade 2) and PAE10 (clade 1). These genes were selected based on our Arabidopsis phylogenetic tree and literature data. Indeed it was reported that AtPAE4 (clade 3), AtPAE5 (clade 3), AtPAE8 (clade 2), AtPAE9 (clade 3), AtPAE10 (clade 1), and AtPAE12 (clade 1) are putative candidates for a minimal set of AtPAEs (AtPAE4/AtPAE5, AtPAE8/AtPAE9, AtPAE10/AtPAE12) important for plant development [15]. In addition, the authors suggested that at least one gene of the pair is important to maintain plant growth. Based on these data and one selected gene per clade, *AtPAE4*, *AtPAE8* and *AtPAE10* were chosen.

For this purpose, a search for structural templates was carried out. There is no plant PAE structure available in the PDB database. The 3D structure of one *Aspergillus aculeatus* rhamnogalacturonan esterase (AacRGAE) has been solved (PDB code: 1PP4) [28]. This fungus PAE belongs to the CE12 family. When one Arabidopsis AtPAE8 was threaded with AacRGAE using the FUGUE algorithm, the structure was severely distorted using the RGAE template, although the root mean square deviation (RMSD) and the template modeling score (TM-score) values were 1.1 Å and 0.55, respectively (Additional file 6). In addition, sequence structure comparison threaded with FUGUE gave an amino acid sequence identity of 17%, which is low. The difference between the sizes of the two proteins and a low pairwise amino acid similarity could corrupt the core structure of AtPAE8. The processed AtPAE8 protein contained 375 amino residues while the RGAE 3D structure contained only 233 residues. This result might highlight some structural differences between PAE from CE12 and CE13. Similar unfolded models were obtained when using AtPAE4 or AtPAE10 (data not shown).

**Fig. 3:**
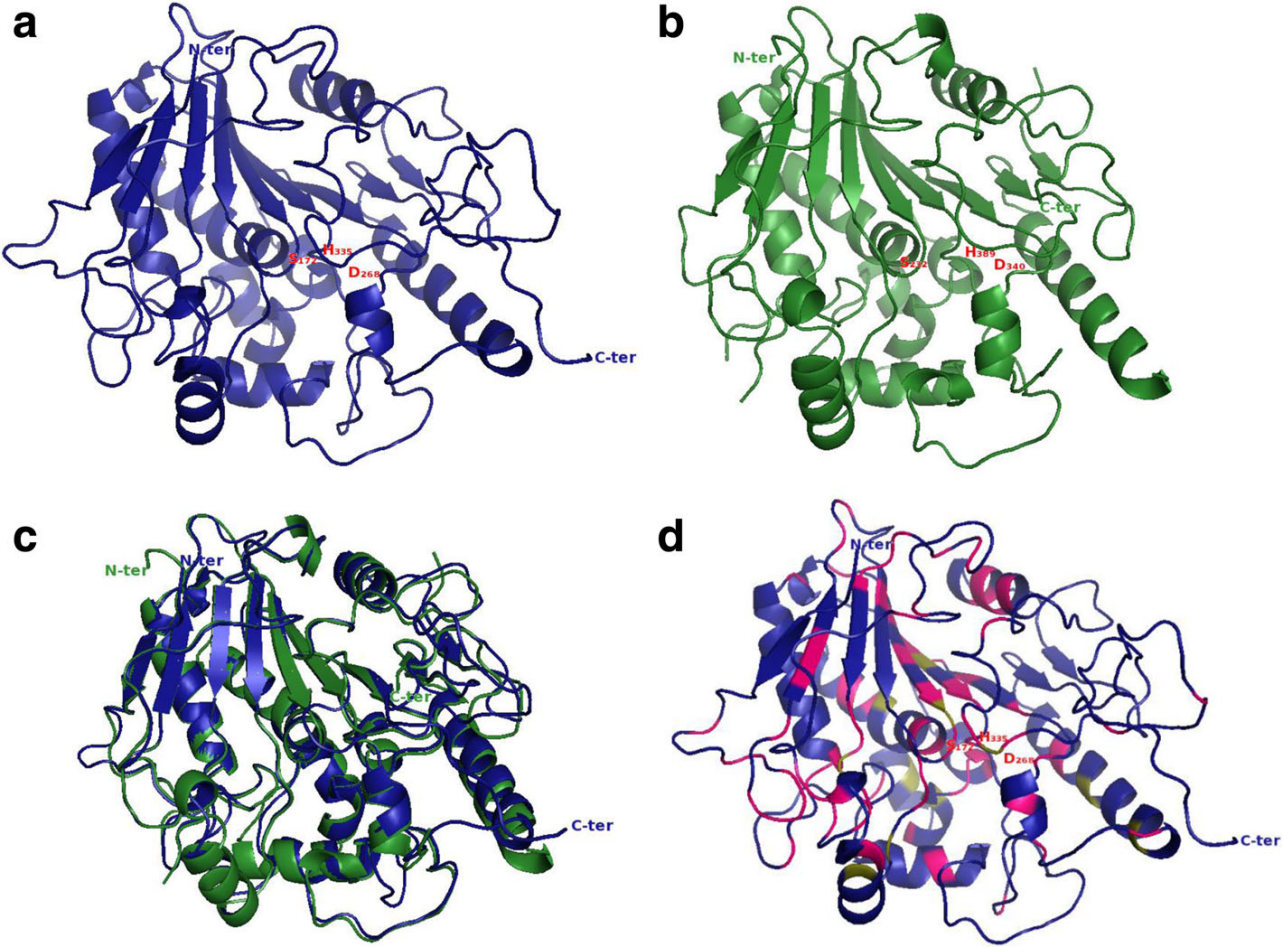
Model of AtPAE8 using a human Notum as template. **a** AtPAE8 model threaded into a human Notum (4UYU_A). **b** Structure of the human Notum (4UYU_A). **c** Structural superimposition between the modeled AtPAE8 structure (*blue*) and the human Notum (*green*). **d** Location of the conserved amino acid residues across the model. The conserved amino acid residues in all Arabidopsis PAEs are depicted in pink and the conserved cysteines are in olive. The putative catalytic triad is in *red*

The structure of AtPAE8 was therefore predicted using LOMETS, a platform of several algorithms [29]. All these servers found a consensus template, a human Notum that has a palmitoleoyl-protein carboxylesterase activity, (PDB code: 4UYU_A). Other good templates could be selected based on our criteria, which were templates from the same family with a similar function (Additional file 7). The hNotum is a monomeric protein that has sequence similarity to the CE13 family proteins [30]. The CE13 family, including plant PAEs, is classified as a SGNH protein family [31]. This superfamily consists of enzymes with diverse hydrolytic functions including lipases, esterases, arylesterases, acyltransferases and carbohydrate esterases [16]. It is further characterized by the presence of four conserved residues, Ser (S), Gly (G), Asn (N) and His (H), where S, H and also a conserved aspartic residue (D) constitute the catalytic triad [16, 17]. The hNotum is a palmitoleoyl-protein carboxylesterase that negatively regulates the Wnt signaling pathway in the animal kingdom [30]. Wnts are secreted signaling proteins that are palmitoylated. Notum removes the palmitoleate moiety from Wnt proteins thus modulating the Wnt signaling pathway [30, 32]. The hNotum, 4UYU, bears the S, D, H catalytic triad of α/β hydrolases [30]. AtPAE8 was threaded with the FUGUE algorithm using 4UYU_A as a template. Sequence structure comparison threaded with FUGUE gave an amino acid sequence identity of 28.4% (Additional file 7), and the threaded structure was consistent with the crystal structure of 4UYU (Fig. 3a, b). In addition, sequence comparison revealed a strong conservation of the catalytic triad (S, D, H), (Additional file 8). The AtPAE8 model preserved the 4UYU triad catalytic core domain (Fig. 3c, d). They shared the same fold since the TM-score was 0.9 and the RMSD value was 0.91 Å. In addition, the Ramachandran plot analysis displayed 86% of amino acid residues in favorable region (Additional files 9 and 10). AtPAE4 and AtPAE10 3D models were obtained in a similar manner. The best template was similar to that used for AtPAE8. However, when the proteins were threaded with FUGUE, AtPAE4 and AtPAE10 structures aligned with chain B instead of chain A. The difference between chain A and chain B is characterized by the absence of the first amino acid residue of the sequence used to generate the 3D structure (Leucine 85) and the presence of a valine residue instead of a leucine residue at position 436 of the hNotum protein sequence. Sequence structure comparison threaded with FUGUE gave an amino acid sequence identity of 30% and 27.5% for AtPAE4 and AtPAE10, respectively, which is in the same range as observed with AtPAE8. The threaded structures were consistent with the crystal structure of 4UYU (Additional file 9). The 3D model of each AtPAE from each clade finally adopted the same fold. The secondary structure matching based on the calculation of RMSD values and the best equivalent residues of two proteins (TM-score) was determined by TM-align [33]. When comparing the models, the best one was obtained with AtPAE8 (0.91 Å). In fact, the RMSD values obtained for AtPAE4 and AtPAE10 were slightly lower: 1.1 and 1.2 Å; respectively. In addition, the accuracy of the predicted models was assessed by Ramachandran plot and showed good proportions of residues in favored, and outlier regions, confirming the model validation (Additional files 9 and 10). The 3D structures of plant PAEs are now needed to confirm the current homology modeling.

Since the three 3D homology models could not provide a consistent explanation related to the classifications of AtPAEs, (Fig. 2a), we explored the primary structure of AtPAEs.

### Primary structure of AtPAEs

**Fig. 4:**
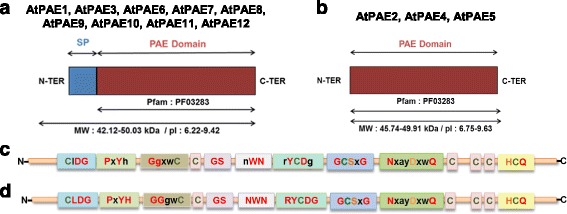
Schematic representation of the predicted domain structure of Arabidopsis PAEs. **a** PAEs with signal peptide (SP). **b** PAEs without SP. All the Arabidopsis PAE proteins display the Pfam domain (PF 032283), characteristic of PAE protein of the CE13 family. The characteristics of these domains are summarized in Additional file 3. **c** Plant PAE conserved motifs. **d** Arabidopsis PAE consensus sequences. The conserved amino acid residues are in red and the similar residues are in *black*. The catalytic triad, S, D, H, is in orange. Cysteine residues are in olive

When the protein sequence of each PAE was analyzed using the Pfam protein databases, the presence of a PAE domain (Pfam: PF03283) that corresponds to the catalytic part of the protein was revealed (Fig. 4a, b). Based on other computational tools including TargetP, ARAMEMNON, and TMHMM for detecting signal peptides and transmembrane domains, nine AtPAEs showed the presence of a signal peptide sequence that was between 20 and 29 amino acid residues in length (Fig. 4a, b, Additional file 4). A signal peptide could not be identified (*p*-value < 0.5) for three AtPAEs (AtPAE2, AtPAE4 and AtPAE5), (Fig. 4b). No transmembrane domain was identified for AtPAEs, indicating that AtPAEs are unlikely to be membrane proteins. A glycosylphosphatidylinositol anchor was predicted for AtPAE10 and AtPAE12 (Additional file 4). AtPAEs had a predicted molecular weight of 39–49 kDa (364–444 amino acids), (Additional file 4). The isoelectric point of the mature AtPAE forms appeared to be basic (8.6–9.4), except for AtPAE4, AtPAE5 and AtPAE6, which displayed a pI between 5.7 and 6.8, indicating a potential acidic pH of activity for these three AtPAEs. These data indicate that AtPAE4 and AtPAE5, which belong to clade 3, have some biochemical specificities that could be associated with a specific function.

To assess whether such differences between isoforms exist in other species, and to determine the putative differences between PAEs from monocots and dicots, the primary structure analysis of the rice PAEs, a grass species, was carried out. OsPAEs displayed similar features compared to AtPAE proteins (Additional file 4). OsPAEs had a predicted molecular weight of 41–51 kDa (374–450 amino acids). All rice PAEs had a predicted signal peptide. The isoelectric point of the mature OsPAE forms appeared more acidic (5.2–6.5).

### Identification of conserved motifs in plant PAEs

We then further investigated the presence of conserved specific motifs found in Arabidopsis and other plant PAEs.

Using members of each clade of Arabidopsis PAE, we used the MultAlin and WebLogo 3 server to identify putative specific motifs [34, 35]. The motifs ERqDTWfaxxS, TxxAKAVGrW and TSxRIxNKTIAE were specific to clades 1, 2 and 3, respectively (Fig. 2b). The motifs were located at the C-terminus of the protein, thus providing a signature for a given clade. Using the Pfam protein databases, the specific motif of each clade did not match with any known domain.

In an attempt to gain more insights into the structure-function of plant PAEs, a multiple sequence alignment of the 72 selected plant PAE protein sequences without their signal peptide was performed using the MUSCLE algorithm [36]. A consensus sequence was detected and inspected to identify the conserved motifs (Fig. 4c, Additional file 11). Inspection of this alignment revealed conserved sequence domains across plant PAEs (Fig. 4c). The conserved amino acids included ClDG and PxYh motifs at the N-terminus. Other conserved motifs, such as gGxwC, GS, nWN and rYCDg, were identified in the first region (N-terminus) of all plant PAE primary sequences. When the multiple sequence alignment was performed on the 12 AtPAEs, the conserved motifs were more specific (CLDG, PxYH, GGxwC, NWN and RYCDG), indicating highly conserved sites across AtPAEs (Fig. 4d). In addition, when a multiple sequence alignment was performed on the grass PAEs, the tryptophan within the motif GGxWC was highly conserved (Additional files 3 and 12). The putative function of these motifs is not yet known. However, based on the literature data and structure alignments performed with SGNH superfamily proteins with known structures, we attempted to assign some functions to them.

**Fig. 5:**
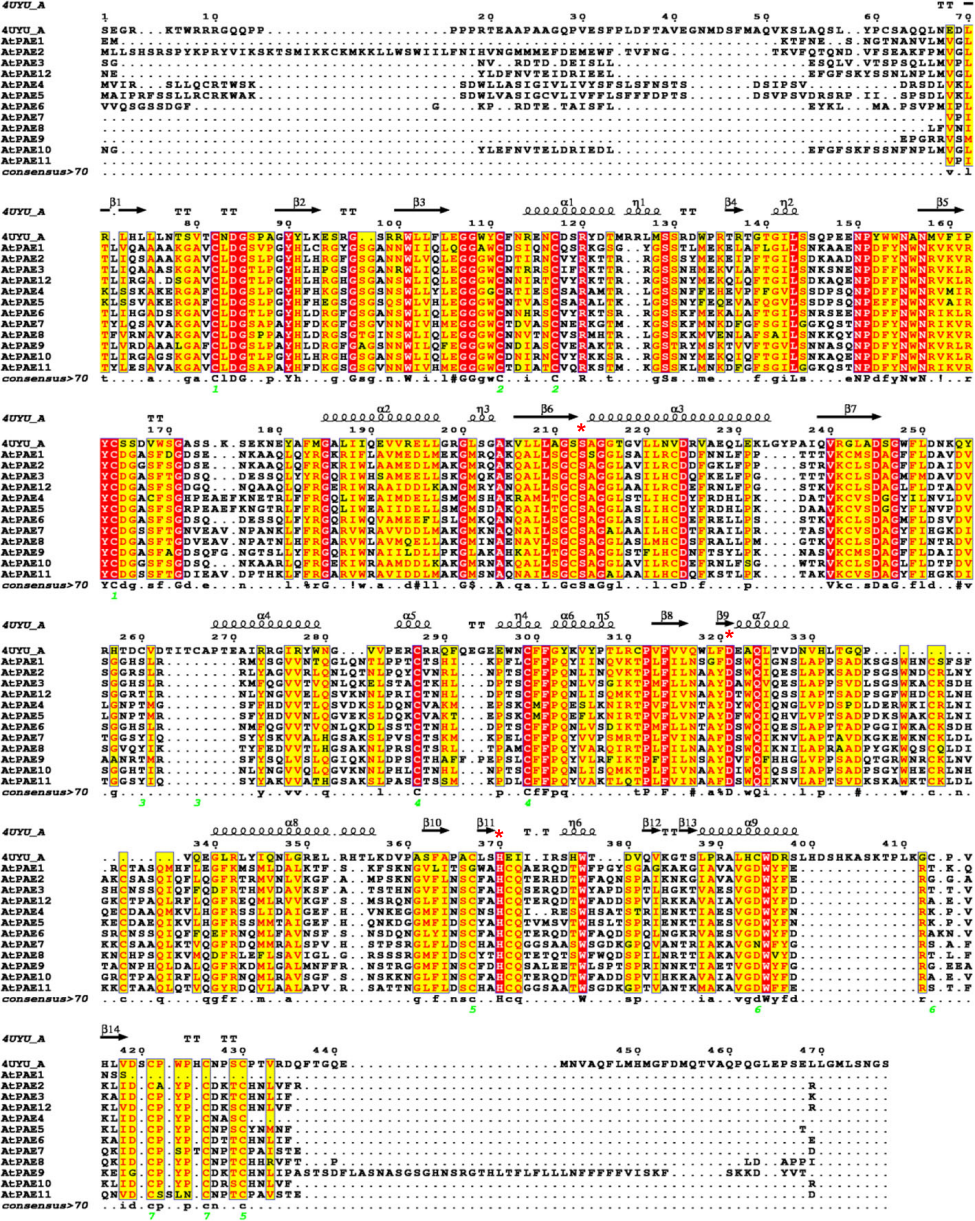
Structure sequence alignment of Arabidopsis PAEs with a human palmitoleoyl-protein carboxylesterase. The PAE sequence retrieved from Uniprot was structure-aligned with one human Notum (PDB code: 4UYU chain A) using Expresso [73] and rendered using ESPript3 [72]. Conserved residues are masked in red (absolutely conserved) or *yellow*. The catalytic triad in 4UYU is indicated by a *red star*. Secondary structure elements of 4UYU_A are shown at the top. α, β, n and T represent α-helix, β-strand, 3_10_ helix and β-turn, respectively

When all AtPAE proteins were structure-aligned with 4UYU, a superimposition of the active site (S, D, H) of 4UYU with identical amino acid residues of all the AtPAEs was observed, revealing a strong conservation of the catalytic site (Fig. 5). The catalytic triad S, D and H was located in the conserved GCSxG, NxayDxxQ and HCQ motifs found in AtPAE and all other plant kingdom PAEs.

These motifs could not be found in PAEs from the CE12 family (Additional file 13). Indeed, when we structure-aligned some characterized bacterial PAEs, including paeY and yxiM, and fungal rhamnogalacturonan acetylesterase (AN2528, rhgT, yesY) with a fungal RGAE (AacRGAE, PDB code: 1PP4) using Expresso from T-Coffee, we identified the conserved GDSX and the DxxH motifs typical of esterases from CE12, which are known to accommodate the catalytic triad (Additional file 13) [12, 28, 30, 37–39]. Members of this family use a highly nucleophilic serine residue as the key catalyst. We hypothesize that the GCSxG motif found in plant PAE is equivalent to the GDSX found in PAE from CE12. In addition, the 3D structure of a kiwi carboxylesterase (AeCXE1, PDB code: 2O7R), which is a SGNH protein like CE12 and CE13 family proteins, showed that the serine residues located in the GxSxG sequence covalently link the acyl group [37]. The presence of the serine residue in the conserved GCSxG motif across plant PAEs is probably the nucleophile in the active site.

The NxayDxwQ motif could be another important motif involved in the catalytic site. In the human Notum protein (PDB code: 4UYU_A), Asp340 and Gln343 contribute to the active site complex of this palmitoleate carboxylesterase [30]. When AtPAEs were structure-aligned with 4UYU, the D and Q amino acid residues of the NxayDxwQ motif co-aligned with D340xxQ343 of hNotum (Fig. 5 and Additional file 8). Furthermore, the 3D structure of *Sinorhizobium meliloti,* Sm23, carbohydrate esterase of family 3 (CE3) belonging, like CE13, to the SGNH superfamily, showed that an asparagine residue (N) contributes to stabilizing the active site geometry and is located close to the aspartic amino acid residue involved in the catalytic triad [40]. Hence, the asparagine residue found in the NxayDxwQ motif could also contribute to the stability of the active site. The last conserved domain at the C-terminus, the HCQ motif, is highly conserved among plant PAEs. The histidine residue is the only conserved histidine in the multiple sequence alignment performed on plant PAEs in our study. In the hNotum (PDB code: 4UYU) whose 3D structure has been characterized, histidine is another important amino acid of the catalytic triad that acts as the base to activate the catalytic Ser residue [30]. When AtPAEs were structure-aligned with 4UYU, the His residue in the HCQ motif co-aligned with the His residue involved in the catalytic site of hNotum (Fig. 5 and Additional file 8). This strongly suggests that the HCQ motif hosts the histidine residue of the active site, but this remains to be formally and experimentally demonstrated.

The presence of aromatic residues (W, Y) and charged amino acids (D) in these conserved motifs could contribute to the substrate binding pocket. There is no plant PAE for which the 3D structure has been determined with or without its substrate, but by homology with other pectin esterases, including tomato pectin methyl esterase whose 3D structure has been solved, the pectin binding site is expected to be lined by several aromatic residues including tyrosine, phenylalanine and tryptophan [41]. The tight hydrophobic interaction between the pectic polymer and the cluster of aromatic rings would keep the substrate bound to the enzyme [42, 43]. Since the AtPAE models adopt an α/β hydrolase fold while PMEs adopt a right-handed parallel β-helix structure [41, 44], we could not superimpose these two different structures to check common conserved amino acid residues, including aromatic residues found to be involved in the pectin binding site in plant PMEs.

Furthermore, when compared to some other carbohydrate acetylesterases, aromatic residues could also contribute to binding by working as anchor residues of the substrate. This has been shown in a carbohydrate acetylesterase, Sm23 (PDB code: 4TX1), from *Sinorhizobium meliloti 1021*, (CE3) in which phenylalanine residues are involved in the positioning of incoming substrate [45]. Similar results have been observed in the crystal structure of a bacterial acetylxylan esterase (*Bacillus pumilus*, BpAXE, PDB code: 3FVR) which is a carbohydrate esterase of family 7 (CE7), in which tyrosine residues accommodate the substrate [46].

In addition to the presence of aromatic residues within these conserved motifs, some conserved glycine residues were found, which could be oxyanion hole residues as shown in the carbohydrate acetylesterase Est24 from *Sinorhizobium meliloti* (PDB code: 5HOE) or in a kiwi (*Actinidia eriantha*) plant carboxylesterase (AeCXE1): the regions containing glycine residues are located on loops involved in substrate recognition [17, 37, 46]. It is known that glycine residues may provide the flexibility needed for enzyme active sites to change conformation [47]. When protein sequence alignment was performed between AtPAEs and AeCXE1 (PDB code: 2O7R), the glycine residues involved in the oxyanion hole of AeCXE1 were structure-aligned with the two glycine residues in the GGgwC motif, indicating a putative similar function of these two glycine residues in the conserved GGgwC motif of Arabidopsis PAEs (Additional file 14).

It should also be noted that a leucine, which is mostly conserved in the ClDG motif of plant PAEs, can be replaced by a methionine residue in some monocot PAEs. The CMDG motif and the highly conserved tryptophan found in the GGxWC motif could thus be together a signature of monocot PAEs, differentiating them from the dicot PAEs studied (Additional file 3b).

### The different functions of PAE in plants

In an attempt to identify some diversification in function among the 12 AtPAEs, we used data from eFP Browser databases to show the expression of AtPAEs in various biological conditions. The data, which correspond to distinct datasets, are normalized and can be compared.

### Plant growth and development

**Fig. 6:**
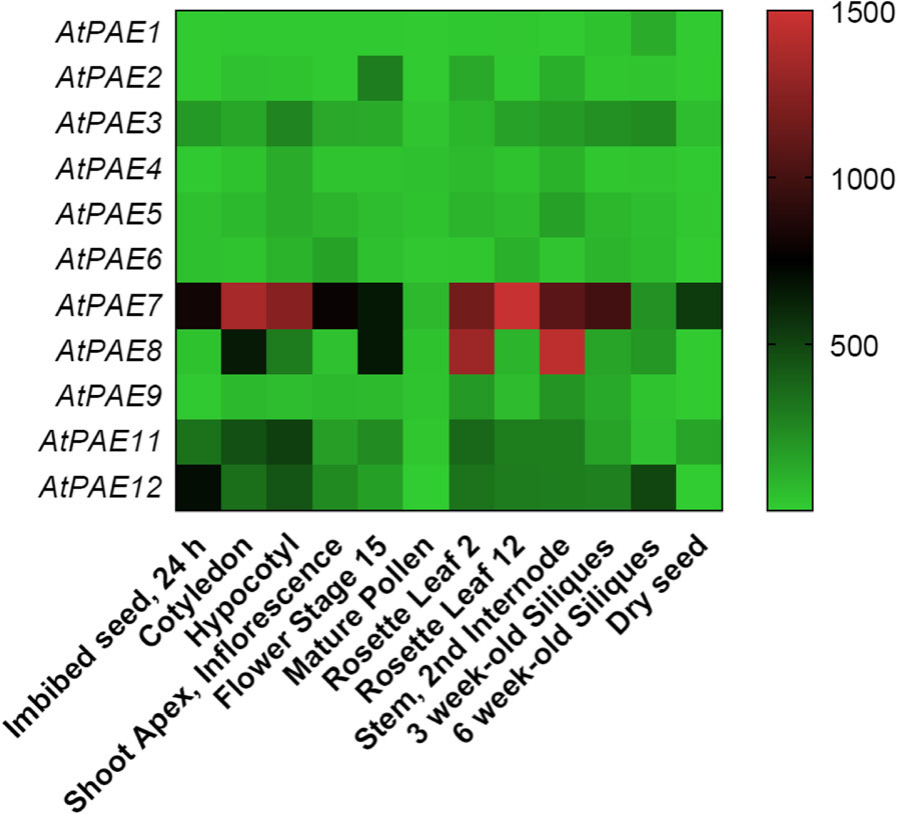
*PAE* gene expression pattern derived from eFP Browser database during development in *A. thaliana.* The expression profile of all *AtPAEs* during plant growth and development was retrieved from the eFP Browser database [79]. Heatmap generation was performed using GraphPad Prism 7.0 software (GraphPad Software, Inc.). The color scale below the heat map indicates expression values; green indicates low gene expression while *red* indicates high gene expression

**Fig. 7:**
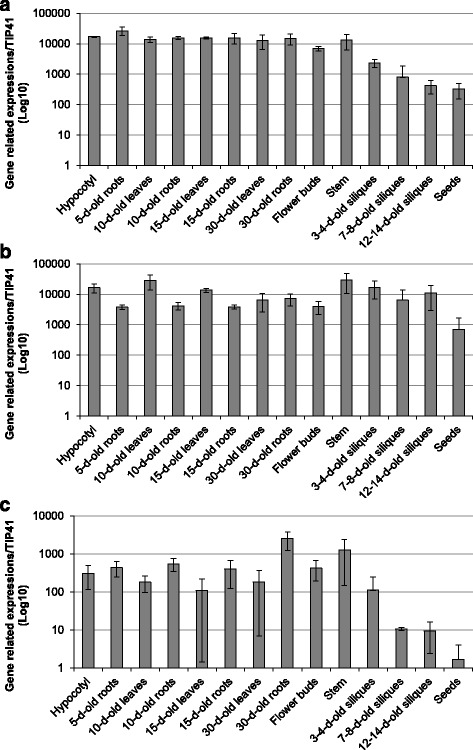
qRT-PCR analysis of the expression levels of *AtPAE4*, *AtPAE8* and *AtPAE10* during different developmental stages. Relative gene expression levels of *AtPAE4* **a**, *AtPAE8* **b** and *AtPAE10* **c** in various organs from Arabidopsis grown on soil were measured using stably expressed reference genes (*Clathrine* and *TIP41*) with similar results. Only the results obtained with *TIP41* are shown. Measurements were carried out in triplicate and values represent means ± SE of three biological replicates. Different letters indicate significantly different expression value at the 0.05 level with the Tukey’s test (Multiple comparisons of means). The first six stages of development are grown on MS medium plates and the others on soil

In the land plant species studied in our work, almost all families except Physcomitrella, which appears to have one single *PAE* gene, contained multiple *PAE* genes. This obviously questions the role of such abundance. In Arabidopsis, data-mining from the eFP Browser database revealed the diversity of *AtPAE* expression patterns. It is noticeable that the *AtPAE10* gene expression profile was not available in eFP Browser database. The expression of AtPAE genes from clade 2 including *AtPAE7* and *AtPAE8* appeared to be highly regulated during plant growth and development (Fig. 6). Some other *PAE* genes from clade 2, including *AtPAE8,* were expressed at specific stages (i.e. flower stage 15 and rosette leaf 12) which could suggest a more specific role in the control of the degree of acetylation of pectins (Fig. 6). *AtPAE11* which belongs to clade 2, was less expressed and mostly induced in the early stages of development (cotyledon and hypocotyl) while *AtPAE12* which belongs to clade 1 was more abundant in seeds (Fig. 6). In contrast, *AtPAE2*, another *AtPAE* from clade 1, seemed to be flower-specific (Fig. 6). The diversity of *AtPAE* gene expression profiles is in line with that observed for other pectin remodeling enzymes and regulators including PMEs, PMEIs, PLLs and PGs [48, 49]. To confirm these data, a quantification of *AtPAE4*, *AtPAE8* and *AtPAE10* gene expression was carried out by qRT-PCR with specific primers for each gene (Fig. 7). *AtPAE4*, *AtPAE8* and *AtPAE10* were expressed in all selected organs but with some variation in expression levels. *AtPAE4* and *AtPAE8* were more abundant in vegetative tissues, including leaves, roots, stem and hypocotyl compared to *AtPAE10*. In addition, *AtPAE8* was more expressed in siliques. *AtPAE4* was more abundant during the different stages of root development than *AtPAE8* and *AtPAE10*. These genes seem to be regulated differentially in the plant organs at the expression level during the plant development.

To assess a correlation between the abundance of transcripts (transcriptomic data) and the presence of the proteins (proteomic data), the cell wall proteome database WallProtDB was used. The WallProtDB database aims at collecting cell wall proteomic experimental data [50]. When using WallProtDB, AtPAE10 was present in roots, while no peptides were identified for AtPAE4 and AtPAE8. But other AtPAEs, including AtPAE2 and AtPAE5 were present in roots. For the other AtPAEs, the WallProtDB database showed that AtPAE7, AtPAE11 and AtPAE12 were present in both leaves and roots. AtPAE9 was found in etiolated hypocotyls and in the stem at late flowering stage, while AtPAE3 was found only in leaves [50]. All these data suggest that a number of PAEs play a key role in controlling the degree of acetylation with consequent effects on development.

### Abiotic stress

**Fig. 8:**
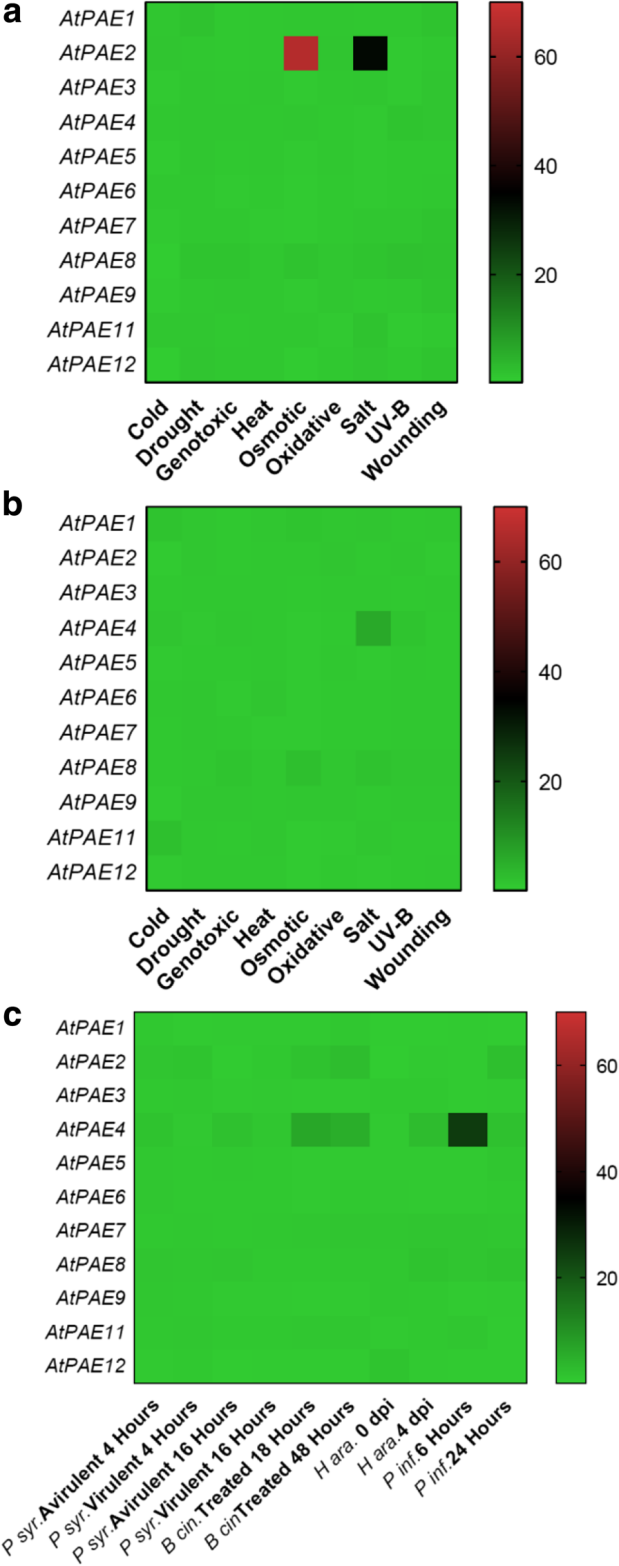
*PAE* gene expression profile derived from the eFP Browser database, in response to different types of abiotic stress in *A. thaliana.* The AtPAE gene expression data in response to abiotic stress from leaves **a**, roots **b** and to different pathogen infections **c** were retrieved from the eFP Browser database [79]. A Heatmap of these data was drawn using GraphPad Prism 7.0 software (GraphPad Software, Inc.). The color scale below the heat map indicates expression values; *green* indicates low gene expression while *red* indicates high gene expression. *(P syr: Pseudomonas syringae; B cin: Botrytis cinerea; H ara: Hyaloperonospora arabidopsis and P inf: Phytophthora infestans)*

In Arabidopsis, the data analysis from eFP Browser databases showed that some specific *AtPAE* genes are differentially regulated in response to diverse abiotic stress. For instance, in shoots, *AtPAE2* (clade 1) was highly induced in response to osmotic stress and to a certain extent to salt stress (Fig. 8a). In roots, *AtPAE4* expression (clade 3) was slightly over-expressed in response to salt stress as was *AtPAE8* (Fig. 8b). All these data indicate that the fine-tuning of the degree of pectin acetylation could be of importance in establishing abiotic stress tolerance mechanisms.

### Biotic stress

The potential contribution of pectin acetylesterase to controlling plant-pathogen interactions can be illustrated by the changes in *AtPAE* gene expression in response to a variety of biotic stresses (Fig. 8c). For instance, *AtPAE4* (clade 3) was more expressed in response to biotic stress, including biotrophic and necrotrophic fungi penetration including *Botrytis cinerea*, *Hyaloperonospora arabidopsis*, and *Phytophthora infestans* (Fig. 8c)*. AtPAE2*, was also induced in response to biotic stress but less compared to *AtPAE4* (Fig. 8c). On the basis of their expression profiles, AtPAE2 and AtPAE4 could play an important role in plant defense.

These data provide evidence that AtPAEs are likely to be involved in biotic stress tolerance and confirm the diversity of their roles in the biotic stress response.

## Discussion

In our study, using bioinformatic approaches, we explored the diversity of plant PAEs in terms of sequence, gene expression and protein structure. Focusing on Arabidopsis PAEs, we believe that we provide novel elements related to this small gene family.

### AtPAEs do not cluster in a similar manner in the phylogenetic tree of the plant kingdom PAEs and in the phylogenetic tree of AtPAEs

We used 72 plant PAEs from the 611 putative PAEs from the plant kingdom available in different databases. A set of criteria was used to select the *PAE* genes. The protein sequences without their signal peptides were retrieved to build a phylogenetic tree. The phylogenetic tree clustered them into five clades where one clade clustered two PAEs, from Moss and Spike moss. Another spike moss PAE (SemoePAE7) clustered with PAEs belonging mostly to monocots. This questions the diversity of PAE among lower plants and could indicate an early diversification of PAE sequences during evolution. In higher plants, we observed a general trend of PAEs with several family members. Some species, including most dicots, display a high number of PAEs while others, such as monocot species, have a low number of PAE members. Similar results have been found in other pectin remodeling enzymes including PMEs, which have 66 gene family members in Arabidopsis compared to rice or Brachypodium with lower numbers of 41 and 42 member genes, respectively [51]. However, the incomplete sequenced genome of some selected species and the incomplete annotation of some plant genomes may not represent the diversity in the plant *PAE* gene family.

The small set of PAEs in lower plants suggests that a given PAE can remove acetyl groups at multiple locations on pectic polysaccharides. Another hypothesis explaining the difference between lower and higher plants regarding the number of PAE genes could be related to their different acetylation status. For example, the degree of acetylation could be smaller in lower plants, which would require less PAE enzyme activity, while in higher plants, pectin de-acetylation appears more complex in terms of substrate specificity given the high number of PAEs for one given species. However, we cannot rule out that the diversification of PAEs might have developed with the increasing complexity of the pectin acetylation process in plants. Furthermore, the presence of tandem duplicated pairs of PAE genes in Arabidopsis, including *AtPAE4*/*AtPAE5* or *AtPAE7*/*AtPAE8*, or duplicated genes with distant genomic locations, including *AtPAE7*/*AtPAE11* or *AtPAE10*/*AtPAE12*, could contribute more quickly to the evolution of the role of acetylation in higher plants.

The tandem duplication phenomenon seems to be important in the gene family of pectin remodeling enzymes [51]. This is the case for pectin methylesterase (*PME*) and its inhibitor (*PMEI*) gene families in which *AtPME3* (*At3g14310*)/ *AtPME26* (*At3g14300*) and *AtPME2* (*At1g53830*)/*AtPME1* (*At1g53840*) are duplicated genes. Similarly, *AtPMEI4* (*At4g25250*) and *AtPMEI7* (*At4g25260*) are gene duplicated. Gene duplication could contribute to the expansion of pectin remodeling enzyme families, which could facilitate the targeting of pectic substrates in a diversity of developmental processes, and could lead to a better stress tolerance, as observed in Arabidopsis and tomato [51–53].

In contrast to other pectin remodeling enzymes including *PME or PG, AtPAE *genes display a high number of introns and exons. The function of this high number of introns (~11) in *AtPAE* genes has not yet been elucidated. It is known that intron-containing genes increase transcription more efficiently than non-intronic genes. They can also act as negative regulators through the generation of intronic microRNA [54]. Finally, they could regulate *PAE* expression in organs or tissues but this has to be demonstrated.

In the phylogenetic tree of plant PAEs, the twelve AtPAEs were clustered into four clades while when we generated a specific tree using only Arabidopsis PAEs, they were grouped into three clades. In the AtPAE phylogenetic tree, AtPAE4, AtPAE5 and AtPAE9 formed clade 3 but in the global phylogenetic tree, AtPAE4 and AtPAE5 were separated from AtPAE9. The difference was due to isoform AtPAE9, which forms a cluster mostly with species having a low number of *PAE* gene family members, probably due to an incomplete sequence annotation of their genomes. In addition, *AtPAE4* and *AtPAE5* are tandem duplicated genes and the pairwise sequence identity of AtPAE4/AtPAE5 is 80%, while the pairwise sequence identity of AtPAE4/AtPAE9 and AtPAE5/AtPAE9 is in the range of 46%. AtPAE9 might have been lost in some plant species while orthologs to AtPAE4 and AtPAE5 might have been more conserved across the plant kingdom. In a distinct phylogenetic tree analysis, it was observed, when analyzing a large number of species, that orthologs to the *AtPAE9* gene were lost in some species including *Arabidopsis lyrata*, *Solanum tuberosum*, *Sorghum bicolor* and *Eucalyptus grandis*. In parallel, it was shown that while orthologs of AtPAE8 were lost in other plant species, including *Capsella rubella*, *Zea mays* and *Setaria italica,* both genes (*AtPAE8* and *AtPAE9*) could not be lost simultaneously. This suggests that the proteins encoded by these two genes are very important for plant growth and development [21].

The phylogenetic tree of plant kingdom PAEs failed to distinguish specific clusters for dicot and monocot PAEs, which was previously observed in a study where all the *PAE* genes from some grasses, including maize, Sorghum, Brachypodium, rice, and two dicot species (poplar and Arabidopsis) were used to build the tree [21]. However, these specific clusters were not observed in another phylogenetic analysis [22]. The discrepancies could arise from the number of species used, the number of sequences, or the parameters used to generate the tree.

### 3D homology modeling of AtPAEs from each clade shows that proteins adopt a common α/β hydrolase fold

The 3D homology modeling of AtPAE4, AtPAE8 and AtPAE10, which belong to distinct clades, was performed. We could not find any plant PAE structure in the PDB database, but the 3D structure of a human Notum protein, which has sequence similarity to CE13 enzymes, was available. Since the 3D structure of a fungal PAE, AacRGAE, is available in the PDB database, we could hypothesize that 3D homology modeling of any AtPAE with that template could be performed. The 3D homology modeling of AtPAE failed when using AacRGAE while when using structure alignment of different PAEs from CE12, the conserved motifs involved in the active site were different from those found in plant PAEs from the CE13 family, indicating that PAEs from CE12 and CE13 do not adopt exactly the same structure. Our 3D models were performed using the human Notum as a template. Like all SGNH family proteins, its structure contains the catalytic triad S, D, H. The 3D homology models obtained with each selected AtPAE had a typical similar α/β hydrolase fold and showed the conserved S, D and H amino acid residues. Although the root mean square deviation value and the Ramachandran plot analysis were acceptable, the percentage of amino acid identity between the template and each AtPAE was too low to observe any particular differences between the 3D models. Finally, these models could not explain the potential diversity among AtPAEs.

### Primary structure of AtPAEs revealed conserved motifs and some specific motifs of each clade

The primary structure of AtPAEs revealed that they are unlikely to be membrane proteins and that most isoforms have a basic pI, as suggested in the early literature [17]. However, the predicted GPI anchor for AtPAE10 and AtPAE12, both from clade 2, could indicate a putative role in plant signaling and some interaction with the plasma membrane. The presence of such a predicted GPI anchor has been observed in other pectin-related genes, including PMEI [55]. The absence of a predicted signaling sequence for three AtPAEs, AtPAE2, AtPAE4 and AtPAE5, indicates that some Arabidopsis PAEs without a signal peptide could have a functional activity. Similar results have been observed with several PMEs from distinct plant species and particularly for AtPME31, which has no signaling sequence but is active and cannot be inhibited by the kiwi pectin methylesterase inhibitor, a strong PME inhibitor [56]. 3D homology modeling of AtPME31 revealed an external loop. The function and the mechanism by which AtPME31 could reach its substrate at the wall have not yet been elucidated [56]. The relevance of such soluble pectin remodeling enzymes remains to be clarified.

We identified a specific motif at the C-terminus for each clade of AtPAEs but the putative function of that motif has to be explored. Other common motifs in plant PAEs were identified and were shown to differ from those found in bacterial and fungal PAEs. The most highly conserved motif across the plant PAEs was the HCQ motif, which contains a histidine residue that could be part of the catalytic site. Other important motifs included GCSxG and NxayDxwQ, which contain other amino acid residues, including S and D, likely to be involved in the catalytic site. This was elucidated when we structure-aligned all the AtPAEs with the 3D structure of the hNotum protein and other esterases, including the 3D structure of a kiwi carboxylesterase (AeCXE1, PDB code: 2O7R), which showed that the serine residues located in the GxSxG sequence of AeCXE1 covalently linked the acyl group [37]. The serine residue in the conserved GCSxG motif across plant PAEs is probably the nucleophile donor to the substrate.

We identified other motifs in all AtPAE proteins, such as CLDG, PxYH, and RYCDG, containing aromatic residues (W, Y) and charged amino acids (D) that could contribute to the substrate binding pocket. The 3D structure of a tomato PME has been solved and has shown that some aromatic acid residues, including tyrosine, phenylalanine and tryptophan, could interact with the pectic substrate [41, 57]. The structure-alignment between AtPAEs and plant PMEs could not provide any evidence of the co-localization of important aromatic residues since AtPAEs adopt an α/β hydrolase fold while plant PMEs have a β-helix structure, (data not shown) [57]. Nevertheless, since these two enzyme families act on pectin, we could assume some common interaction mechanism between the protein and the substrate. In addition, the literature data on carbohydrate acetylesterases, including a bacterial acetylxylan esterase, shows that aromatic residues could also contribute to binding by working as anchor residues of the substrate [46].

However, this suggestion needs to be formally and experimentally demonstrated. The expression of *AtPAEs* could relate to a diversification of their function. We used the eFP Browser database to determine the expression pattern of *AtPAE* genes in various biological conditions. In addition, the cell wall proteomics database, WallProtDB, was used to identify the abundance of the different AtPAEs in various tissues.

AtPAE7, AtPAE8 and AtPAE11 from clade 2 appear to be specifically expressed during plant growth and development. The high transcript level of these genes encoding these proteins, compared to the other *AtPAEs* indicates that proteins encoded by them are essential in Arabidopsis growth and development. The presence of peptides from AtPAE7 and AtPAE11 in roots and leaves identified in the WallProtDB database is in agreement with the data from eFP Browser and our in-house data. No peptides corresponding to AtPAE8 could be identified in the WallProtDB database but peptides mapping AtPAE9 which belongs to the putative pair of AtPAEs (AtPAE8/AtPAE9) important for plant growth could be identified in leaves, inflorescence stem and hypocotyls. The tandem duplicated genes *AtPAE7* (*At4g19410*)/*AtPAE8* (*At4g19420*) and the pair *AtPAE7* (*At4g19410*)/*AtPAE11* (*At5g45280*) which displays 83% pairwise protein sequence identity, strengthen the hypothesis that these genes are essential to maintain growth and development in Arabidopsis. Our qRT-PCR data showed that *AtPAE4* is also broadly expressed during plant growth and development. The quantification of *AtPAE5* expression by qRT-PCR revealed that *AtPAE5* shared the same expression pattern as *AtPAE4* but *AtPAE5* is more abundant than *AtPAE4* (Additional file 15). But, when using the WallProtDB database, no peptides mapping AtPAE4 was found, while peptides assigned to AtPAE5 were detected in root tissue. The discrepancies between the gene expression pattern and the proteomic data suggest that AtPAE5 which belongs to the tandem duplicated *AtPAE4*/*AtPAE5* genes, seems to be essential for plant development. All these data, finally emphasizes that there is no obvious correlation between the abundance of transcripts and the presence of the proteins [58].

The role of PAEs in plant development has so far remained elusive. The overexpression of one poplar *PAE *gene (*PtPAE1*) in tobacco plants led to a severe phenotype in floral development and to reduced formation of pollen grains with consequent severe sterility [5]. In potato tuber, the overexpression of a mung bean *PAE* induced a 39% decrease in the degree of acetylation of the tuber cell wall material, which altered cell wall mechanical properties by increasing the stiffness of the potato tuber tissue [9]. In addition, analysis of T-DNA mutant lines for *AtPAE8*, *AtPAE9,* as well as the double mutant *atpae8/atpae9* showed shorter inflorescence stems for *atpae9* (10%) and the double mutant (21%) [20]. The acetate content in the pectic extract of the *atpae8* mutant and the double mutant *atpae8/atpae9* was 20% and 37% higher, respectively, compared to the wild type.

Data retrieved from eFP Browser showed that *AtPAE2* (clade 1) and *AtPAE4*, (clade 3) genes were mainly regulated in response to different abiotic and biotic stresses. The regulation depended on the stress and the tissues studied. The cell wall plays an important role in response to abiotic stress (for a review, see [57]), but the effect of cell wall acetylation in this context is far from being understood. It has been shown that levels of *O*-acetylation of xyloglucan and pectin are reduced in a water deficit environment and ozone stress, respectively [59, 60]. *PAE* genes are induced in response to aluminum stress in *Medicago truncatula,* and in *Populus tremuloides* grown in increased levels of atmospheric CO_2_ [61, 62]. During pathogen infection, the cell wall acts as a barrier involved in the plant defense mechanism. Cell wall degradation can produce oligogalacturonides (OG) with a signaling function [63]. Moreover, the accumulation of OG fragments, which are considered damage associated molecular patterns (DAMPs), can induce resistance in Arabidopsis and tobacco (*Nicotiana tabacum*) that overexpress a microbial polygalacturonase including an *Aspergillus niger* PGII [64]. In the cell wall, biotic stress can alter the expression of genes encoding homogalacturonan-modifying enzyme (HMGE), [49].

The degree of pectin acetylation appears to be a key feature of the biotic stress response, particularly in wheat (*Triticum aestivum*) in interaction with mildew. Treatment with acetylated OG before infection induced a decrease in fungal haustoria growth [65]. In Arabidopsis, when the pectin acetylation pathway was altered, especially in the reduced wall acetylation 2 mutant (rwa2) that displayed a 20% reduction in cell wall acetylation, an increased resistance to *Botrytis cinerea* was observed [66]. In another study, the overexpression of a fungal pectin acetylesterase (*Aspergillus nidulans*) in *Arabidopsis thaliana* resulted in a tolerance to *Botrytis cinerea* [63]. These findings show that the cell wall, and particularly the tuning of pectin acetylation, is important in the signaling pathway of plant defense against biotic stress.

## Conclusions

The present bioinformatic analysis provides new insights into the structure, expression and potential function of plant PAEs. The phylogenetic tree analysis divides plant PAEs into five clades and Arabidopsis PAEs into three clades. Plant kingdom PAEs share conserved motifs. The functional analysis of these motifs is required to understand the structure-function relationship of plant PAEs. Site-directed mutagenesis could be a useful tool to characterize and confirm the amino acid residues involved in the putative catalytic and pectin binding sites. Reverse genetics analysis and phenotyping will enable the precise role of Arabidopsis PAEs during plant and growth development and in response to environmental conditions to be determined. All these future data will provide evidence that the acetylation of pectin plays a role in the control of the mechanical properties of the cell wall.

## Methods

### Data resources

The 611 putative plant PAE sequences were retrieved from Phytozome (https://phytozome.jgi.doe.gov/pz/portal.html), PlantCAZyme (http://cys.bios.niu.edu/plantcazyme/) and Uniprot (http://www.uniprot.org/) databases.

The PAE sequences were chosen using different criteria, including extensive sequence annotation of the PAE protein of some genomes, availability of full-length protein sequence, model species as well as relevant literature on plant *PAE* gene function. PAE sequence annotation based on only ESTs were not selected. For *PAE* genes displaying alternative splicing, only one isoform was selected from the UniProt database.

### Protein primary sequence analysis

The presence of a signal peptide was predicted using TargetP [67]. ARAMEMNON consensus prediction was used to confirm the prediction of signal peptides [68]. The isoelectric point and molecular weight were predicted using the Compute pI/Mw computational tool [69]. The glycosylphosphatidylinositol anchor was predicted using the PredGPI tool [70].

### Phylogenetic analyses

The phylogenetic analyses of the amino acid sequence were performed using MEGA 7 [27]. To build the tree, multiple sequence alignment of protein sequences without their signal peptides was carried out according to [27]. To generate phylogenetic trees, the maximum likelihood method (MLM) and Neighbor-Joining method were used with 1000 bootstrap replicates. A BLOSUM60 was used to generate the plant PAE multiple sequence alignment while a BLOSUM80 was used to build the Arabidopsis PAE multiple sequence alignment. The evolutionary distances were built using the JTT matrix-based method [71].

### Protein alignment

Multiple sequence alignment of PAEs without their signal peptides incorporating primary structure information were aligned using MUSCLE [36]. Pairwise sequence identity and similarity were obtained from multiple sequence alignment carried out with MUSCLE [36] and further used with SIAS and multalin (http://imed.med.ucm.es/Tools/sias.html), [35]. The motif logo pictures were generated by WebLogo (http://weblogo.berkeley.edu/logo.cgi), [34, 35]. For the structural alignment of AtPAE*, the* proteins were structure-aligned with 4UYU using Expresso from T-Coffee [72]. The primary and secondary structure alignment of PAE was rendered using ESPript3 [73].

### 3D homology modeling

Because no 3D structure of plant PAEs was available in the Protein Data Bank [74], homologous sequences with known structures as templates were identified using LOMETS [29]. AtPAE8 without its signal peptide was modeled using the human Notum (PDB code: 4UYU_A) [30]. Other good templates, including 3FVR_A, 1L7A_A, and 1VLQ_A, were selected based on sequence identity, secondary structure comparison, protein family structure and good query coverage. Templates selected as target proteins for alignment of the AtPAE8 sequence were threaded with FUGUE [75]. AtPAE4 and AtPAE10 were modeled with chain_B of the 4UYU structure instead of chain A based on sequence-structure comparison using FUGUE. The tertiary structure was modeled with Modeller 9v16 [76], based on the sequence-structure alignment obtained from FUGUE. The constructed structural model was visualized and labeled in PyMol software [77]. All the predicted quality models were evaluated by a protein model quality assessment method, TM-align [33]. TM-align employs the secondary structure based on a structure matching algorithm [33]. The Ramachandran plot was calculated using MolProbity and the visualization was carried out by PyMOL [77, 78].

### AtPAEs expression profile from eFP Browser

The expression profile of all AtPAEs was retrieved from the eFP Browser database [79]. In this database, the expression level of the different genes was quantified using normalization methods for Arabidopsis plant development [80] and global stress [81]. These data were imported into GraphPad prism 7.0 software (GraphPad Software, Inc.). A Heat Map was drawn for gene expression data.

### RNA extraction and qRT-PCR

Arabidopsis hypocotyls, leaves and roots were collected from 5-day-, 10-d- and 15-day- old growth on plates with 1/2 MS medium. The other tissues were harvested from plants growing on soil. The samples were flash-frozen in liquid nitrogen and ground into powder. All samples were prepared from three independent biological replicates. Total RNAs were extracted from the tissue, using hot acidic phenol extraction [82]. The cDNA was generated with 3 μg of RNA by the SuperScript™ III enzyme and the SuperScript™ III First-Strand Synthesis SuperMix using the manufacturer’s protocol (Invitrogen; Cat. No. 18080–400). The cDNA diluted to 1/20 was used for semi-quantitative and qRT-PCR analyses. One primer pair for each gene including *AtPAE4*, *AtPAE8* and *AtPAE10* was used for this study (Additional file 16). The qRT-PCR was carried out with the LightCycler® 480 SYBR Green I Master (Roche, Indianapolis, IN, USA; Cat. No. 04887352001) in 384-well plates in the LightCycler® 480 Real-Time PCR System (Roche). The CT values for each sample (crossing threshold values are the number of PCR cycles required for the accumulated fluorescence signal to cross a threshold above the background) were acquired with the LightCycler® 480 software (Roche) using the second derivative maximum method. We used geNorm software to select the references genes. *TIP41* and Clathrin displayed the most stable expression during the development of various organs tested [83]. But *TIP41* gene was considered as the best reference gene according to geNorm [83]. TIP41 was used to calculate the relative expression of target genes, according to the method described by [84].

### Statistical analysis

Statistical analysis was performed using ANOVA and Tukey’s test. (*P < 0.05) by the statistical analysis software R (http://www.r-project.org).

## Additional files

Additional file 1: The number of *PAE* genes in the plant kingdom. 611 putative plant PAE proteins without their signal peptide were obtained from various sources including Phytozome, PlantCAZyme and Uniprot databases. (PDF 44.3 kb)

Additional file 2: The plant *PAE* genes used in this study. The 72 plant PAEs were selected from the 611 putative PAEs. (PDF 264 kb)

Additional file 3: Phylogenetic tree of grass PAEs along with AtPAEs and schematic representation of the predicted motifs found in the grass PAEs. (a) The evolutionary history was inferred by using the Maximum Likelihood method based on the JTT matrix-based model [71]. Evolutionary analyses were conducted in MEGA7 [27]. Each major clade is identified with a specific color. Clade 1 is in pink, clade 2 in purple, clade 3 in green and clade 4 in blue. (b) PAE conserved motifs in grass PAEs. (PDF 14.7 kb)

Additional file 4: Features of putative Arabidopsis and rice PAEs. The name, locus, accession number, length, MW, and pI are given. (PDF 57.7 kb)

Additional file 5: Pairwise sequence identity (a) and similarity (b) from multiple sequence alignment of AtPAE proteins without their signal peptide using Muscle [36]. Percentage identity and similarity of amino acid residues were determined using SIAS (http://imed.med.ucm.es/Tools/sias.html). (PDF 28.3 kb)

Additional file 6: 3D homology modeling of AtPAE8 with a rhamnogalacturonan acetylesterase from *Aspergillus aculeatus*. (a) AtPAE8 3D model threaded with an *Aac*RGAE (PDB code: 1DEO) using FUGUE [75]. (b) Structure of *Aac*RGAE [28]. (c) Pairwise structure alignment between AtPAE8 and *Aac*RGAE using T-coffee and rendered with ESPript3 [73]. (PDF 394 kb)

Additional file 7: Templates used for threading. (PDF 847 kb)

Additional file 8: Sequence-structure comparison between the amino acid sequence of AtPAE8 and a human palmitoleoyl-protein carboxylesterase (4UYU_A) threaded with FUGUE [75]. The catalytic triad in 4UYU is shown in dark red; other important amino acid residues involved in 4UYU activity are depicted in light red; amino acids involved in the substrate binding pocket are shown in blue. A cluster of 12 or 13 cysteine residues is in green [30]. The amino acid sequence of each protein does not contain the signal peptide. (PDF 48 kb)

Additional file 9: Structural evaluation of the 3D models of AtPAE4, AtPAE8 and AtPAE10 using Ramachandran plot analysis. Each model was compared to the template 4UYU (in dark green). The Ramachandran plot was visualized using Pymol [77]. The 3D models of AtPAE4, AtPAE8 and AtPAE10 are in red, blue and olive, respectively. The square and triangle symbols represent proline and glycine residues respectively. The other amino acid residues are depicted in circle. (XLSX 8.79 kb)

Additional file 10: The Ramachandran plot statistics of AtPAE4, AtPAE8 and AtPAE10 3D models. The validation for 3D structures of AtPAEs was provided using MolProbity [77]. (XLSX 70 kb)

Additional file 11: Multiple sequence alignment of plant PAEs. The PAE sequences retrieved from Uniprot were aligned using Muscle [36]. Conserved residues are marked in red (absolutely conserved). (XLSX 15.3 kb)

Additional file 12: Multiple sequence alignment of grass PAEs. The PAE sequences retrieved from Uniprot were aligned using Muscle [36]. Conserved residues are marked in red (absolutely conserved). (PDF 166 kb)

Additional file 13: Structure sequence alignment of bacterial and fungal PAEs with a fungal RGAE. The PAE sequences from some bacteria (paeY, yxiM) and fungi (yesY, rhgT, AN2528) were retrieved from Uniprot and structure-aligned with one fungal rhamnogalacturonan acetylesterase (PDB code: 1PP4) using Expresso and rendered using ESPript3 [72, 73]. Conserved residues are masked in red (absolutely conserved) or yellow. The catalytic triad in 1PP4 is indicated by a red star. Secondary structure elements of 1PP4 are shown at the top. α, β, n and T represent α-helix, β-strand, 3_10_ helix and β-turn, respectively. (XLSX 13.3 kb)

Additional file 14: Structure sequence alignment of AtPAEs with a carbohydrate acetylesterase from kiwi (AeCXE1, PDB code: 2O7R). The AtPAEs and the kiwi carbohydrate acetylesterase (AeCXE1) sequences without their signal peptide were retrieved from Uniprot and structure-aligned with AeCXE1 (PDB code: 2O7R) using Expresso [72] and rendered using ESPript3 [73]. The two conserved glycine residues involved in the active site are displayed with a blue star. α, β, n and T represent α-helix, β-strand, 3_10_ helix and β-turn, respectively. (PDF 9.88 kb)

Additional file 15: qRT-PCR analysis of the expression levels of *AtPAE5* during different developmental stages. Relative gene expression levels of *AtPAE5* in various organs of Arabidopsis grown on soil were measured using stably expressed reference genes (*Clathrine* and *TIP41*) with similar results. Only the results obtained with *TIP41* are shown. Measurements were carried out in triplicate and values represent means ± SE of three biological replicates. Different letters indicate significantly different expression at the 0.05 level with the Tukey’s test. (PDF 174 kb)

Additional file 16: Primer sequences designed from the cDNA sequence of *AtPAE4*, *AtPAE8* and *AtPAE10*. (PDF 87 kb)

## Abbreviations

PAE: Pectin acetylesterase
PME: Pectin methylesterase
PMEI: Pectin methylesterase inhibitor
PG: Polygalacturonase
PLL: Pectate lyase-like
