# Supplementary material for: Plant pectin acetylesterase structure and function: new insights from bioinformatic analysis
Source: BMC Genomics. 2017 Jun 8;18:456. doi: 10.1186/s12864-017-3833-0 (PMC5465549; doi:10.1186/s12864-017-3833-0)
Supplement: Supplementary file 1 — The number of PAE genes in the plant kingdom. 611 putative plant PAE proteins without their signal peptide were obtained from various sources including Phytozome, PlantCAZyme and Uniprot databases. (PDF 44.3 kb) [file 12864_2017_3833_MOESM1_ESM.pdf]

Additional file 1.

```
23   --LFVNITFVRNAVAKGAVCLDGSPPAYHLDRGSGTGINSWLIQLEGGGWCNNVTNCVSRMHT--RLGSS      AtPAE8
85   LNEDLRLHLLLN---TSVTCNDGSPAGYYLKESRGS--RRWLLFLEGGWYCFNRENCDSRYDTMRRLMSS      4UYU_A

89   KKMVENLAFSAILSNKKQYNPDFYNWNRVKVRYCDGASFTGDVEAVNPATNLHFRGARVWLAVMQELLAK      AtPAE8
150  RDWPRTRTGTGILSSQPEENPYWWNANMVFIPYC-----SSDVWSGASSKEYAFMGALIIQEVVRELLGR      4UYU_A

159  GMINAENAVLSGCSAGGLASLMHCDSFRALLPM----GTKVKCLSDAGFFLNTRDVSGVQYIKTY--FED      AtPAE8
219  GLSGAKVLLLAGSSAGGTGVLLNVDRVAEQLEKLGYPAIQVRGLADSGWFLDNKQYRHTDCVDTITCAPT      4UYU_A

223  VVTLHGSAK---NLPRSCTSRLTP---AMCFFPQYVARQIRTPLFILNAAYDSWQIKNILAPRAADPYGK      AtPAE8
289  EAIRRGIRYWNGVVPERCRRQFQEGEEWNCFFGYKVYPTLRSPVFVVQWLFDEAQLTV-------DNVHL      4UYU_A

287  WQSCQLDIKNCHPSQIKVMQDFRLEFLSAVIGLGRSSSRGMFIDSCYTHCQTETQTSWFWQDSPILNRTT      AtPAE8
357  QEGLRLYIQNLGRELRHTLKDVPASFAPA----------------CLSH---EIIIRSHWTDVQV-KGTS      4UYU_A

357  IAKAVGDWVYDRTLFQKID-CPYP-CNPTCHH----RVFTPLDAPPI----------------------*      AtPAE8
407  LPRALHCWDRSLCPVHLVDSCPWPHCNPSCPT-------------------------------------*      4UYU_A
```

Philippe *et al.*,
