# Supplementary figures and images for: Plant pectin acetylesterase structure and function: new insights from bioinformatic analysis
Source: BMC Genomics. 2017 Jun 8;18:456. doi: 10.1186/s12864-017-3833-0 (PMC5465549; doi:10.1186/s12864-017-3833-0)

Additional file 2.

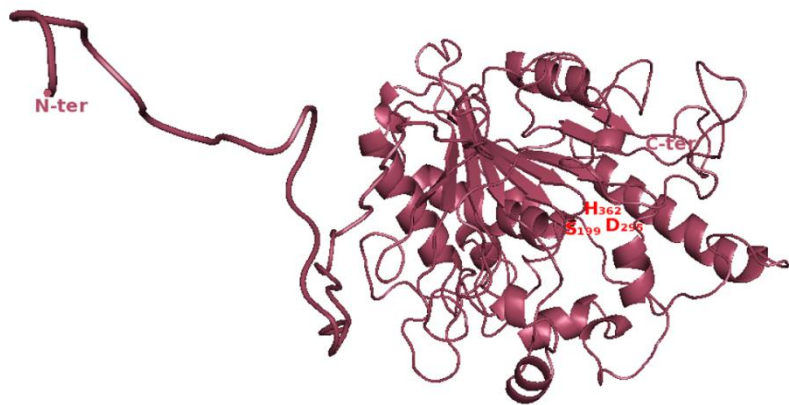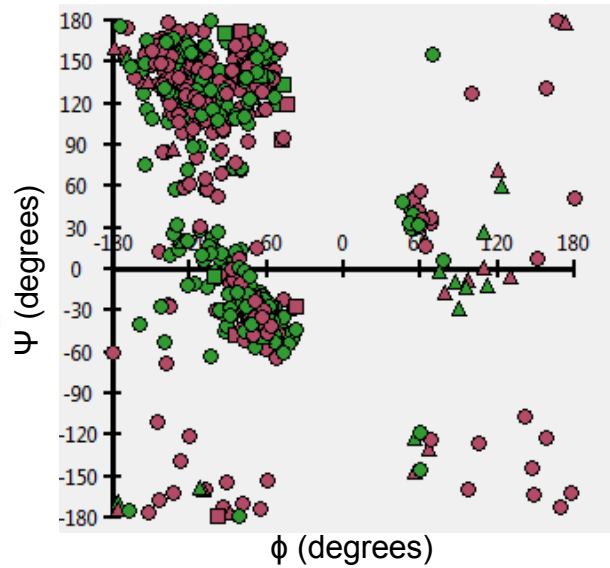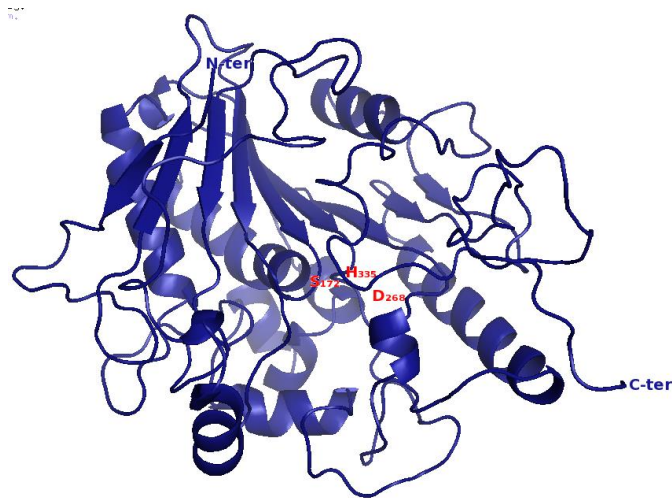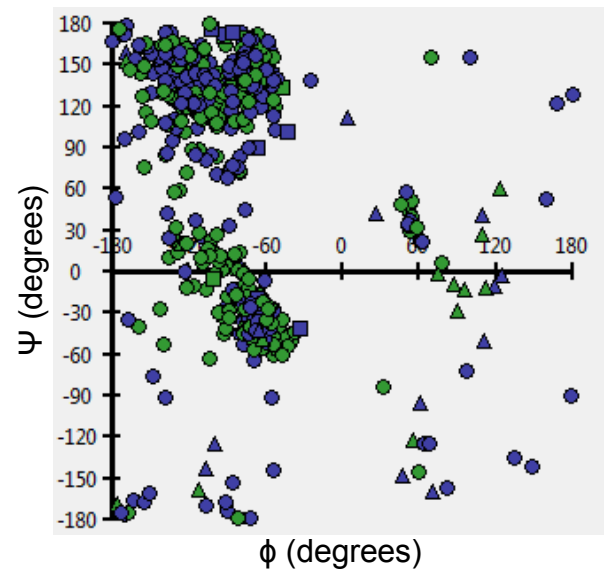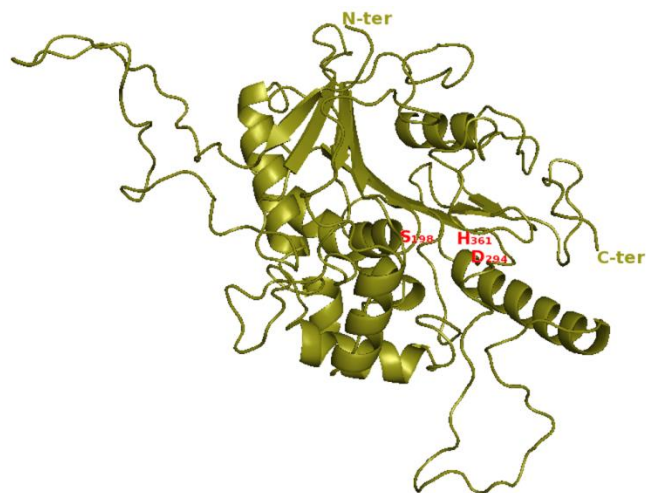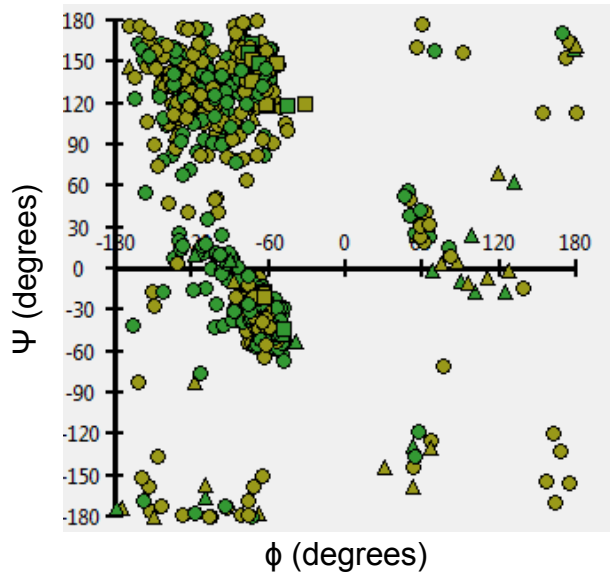

Supplement: Supplementary file 2 — The plant PAE genes used in this study. The 72 plant PAEs were selected from the 611 putative PAEs. (PDF 264 kb) [file 12864_2017_3833_MOESM2_ESM.pdf]

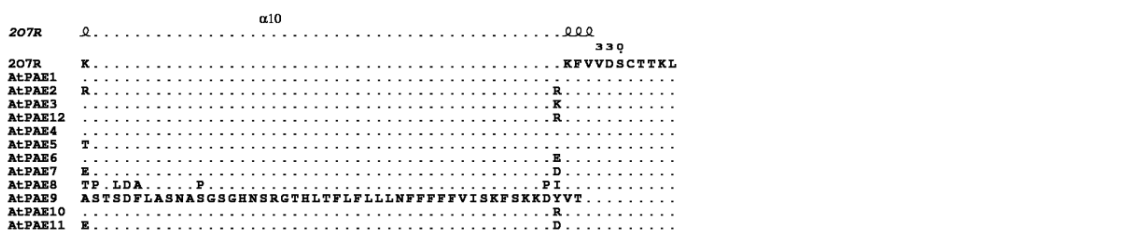

Supplement: Supplementary file 7 — Templates used for threading. (PDF 847 kb) [file 12864_2017_3833_MOESM7_ESM.pdf]

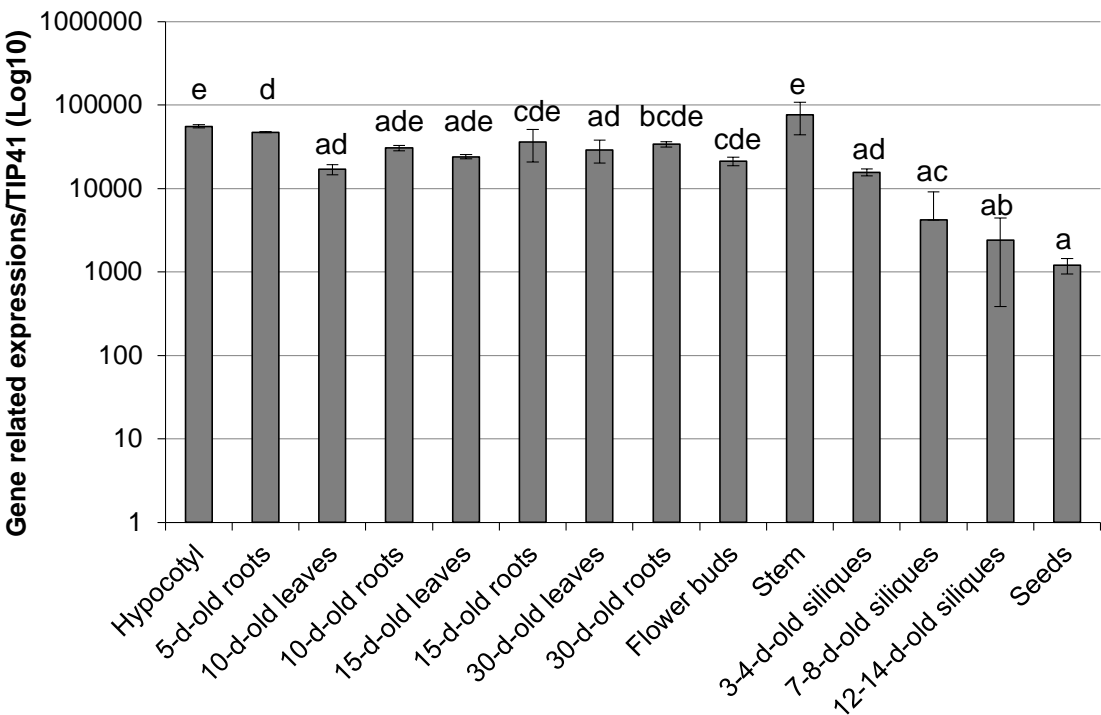

Supplement: Supplementary file 8 — Sequence-structure comparison between the amino acid sequence of AtPAE8 and a human palmitoleoyl-protein carboxylesterase (4UYU_A) threaded with FUGUE [75]. The catalytic triad in 4UYU is shown in dark red; other important amino acid residues involved in 4UYU activity are depicted in light red; amino acids involved in the substrate binding pocket are shown in blue. A cluster of 12 or 13 cysteine residues is in green [30]. The amino acid sequence of each protein does not contain the signal peptide. (PDF 48 kb) [file 12864_2017_3833_MOESM8_ESM.pdf]

a

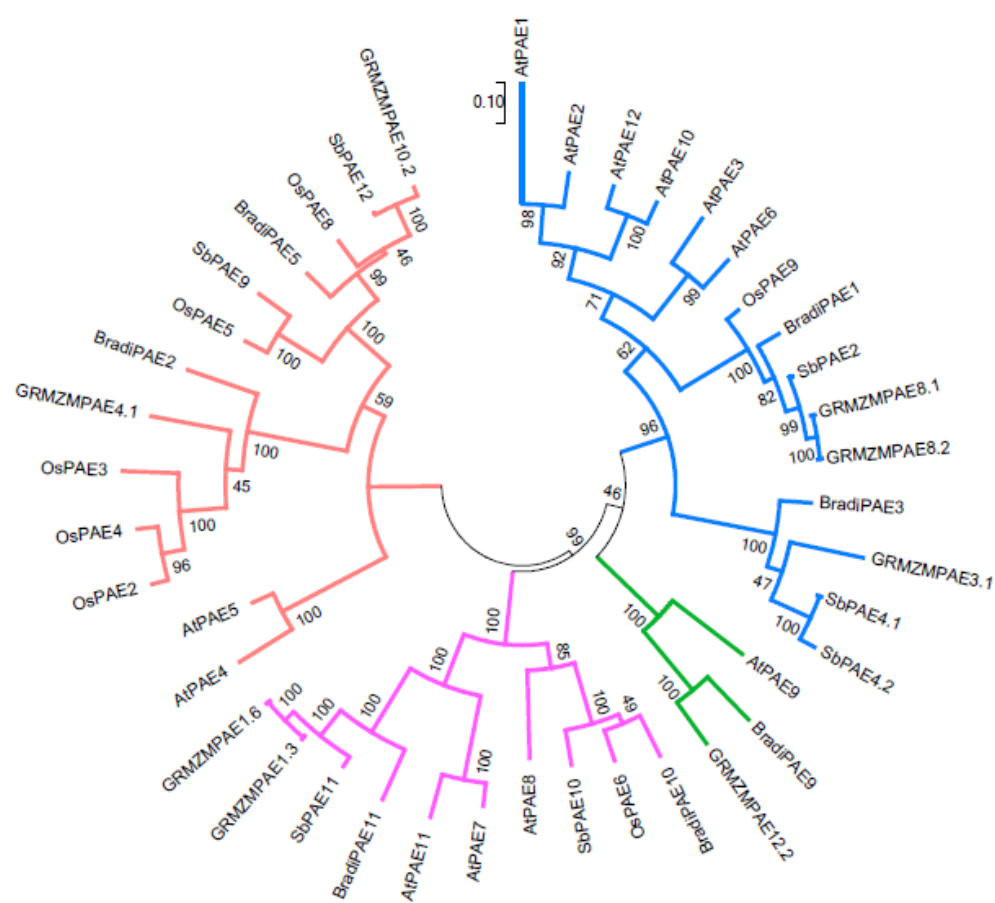

b

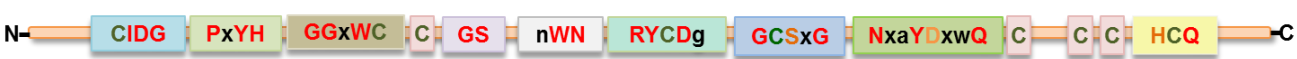

Supplement: Supplementary file 12 — Multiple sequence alignment of grass PAEs. The PAE sequences retrieved from Uniprot were aligned using Muscle [36]. Conserved residues are marked in red (absolutely conserved). (PDF 166 kb) [file 12864_2017_3833_MOESM12_ESM.pdf]

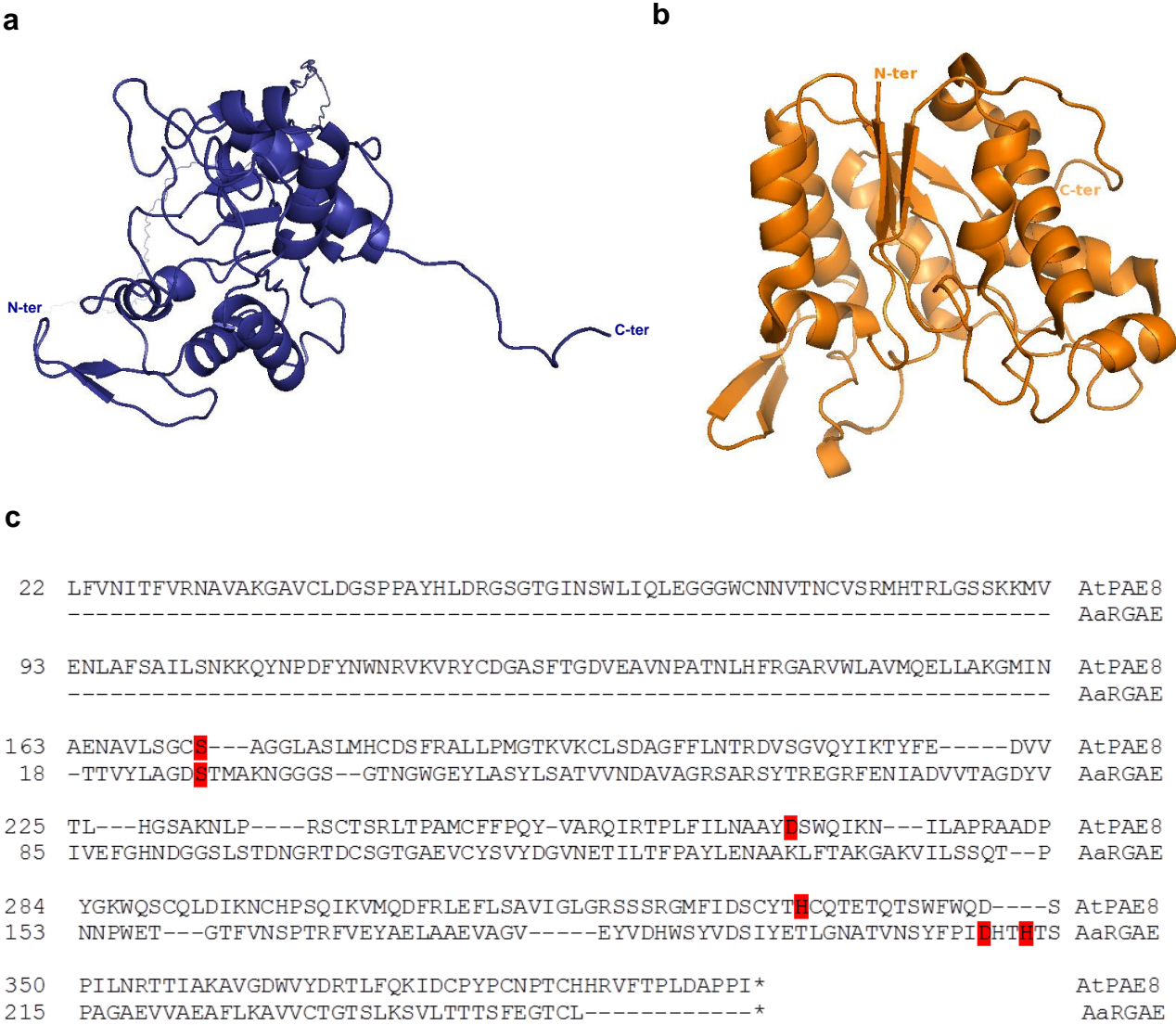

Supplement: Supplementary file 15 — qRT-PCR analysis of the expression levels of AtPAE5 during different developmental stages. Relative gene expression levels of AtPAE5 in various organs of Arabidopsis grown on soil were measured using stably expressed reference genes (Clathrine and TIP41) with similar results. Only the results obtained with TIP41 are shown. Measurements were carried out in triplicate and values represent means ± SE of three biological replicates. Different letters indicate significantly different expression at the 0.05 level with the Tukey’s test. (PDF 174 kb) [file 12864_2017_3833_MOESM15_ESM.pdf]
