# Supplementary material for: Plant pectin acetylesterase structure and function: new insights from bioinformatic analysis
Source: BMC Genomics. 2017 Jun 8;18:456. doi: 10.1186/s12864-017-3833-0 (PMC5465549; doi:10.1186/s12864-017-3833-0)
Supplement: Supplementary file 3 — Phylogenetic tree of grass PAEs along with AtPAEs and schematic representation of the predicted motifs found in the grass PAEs. (a) The evolutionary history was inferred by using the Maximum Likelihood method based on the JTT matrix-based model [71]. Evolutionary analyses were conducted in MEGA7 [27]. Each major clade is identified with a specific color. Clade 1 is in pink, clade 2 in purple, clade 3 in green and clade 4 in blue. (b) PAE conserved motifs in grass PAEs. (PDF 14.7 kb) [file 12864_2017_3833_MOESM3_ESM.pdf]

Additional file 3.

	Protein geometry	Number of residues in percentage (%)	Percentage (%) expected
**AtPAE4**	Ramachandran favored	87.71	>98
	Ramachandran outliers	6.14	<0.05
	Favored rotamers	89.42	>98
	Poor rotamers	3.9	<0.3
**AtPAE8**	Ramachandran favored	86.33	>98
	Ramachandran outliers	5.09	<0.05
	Favored rotamers	89.06	>98
	Poor rotamers	3.44	<0.3
**AtPAE10**	Ramachandran favored	87.56	>98
	Ramachandran outliers	4.57	<0.05
	Favored rotamers	85.33	>98
	Poor rotamers	5.39	<0.3
