# Supplementary material for: Plant pectin acetylesterase structure and function: new insights from bioinformatic analysis
Source: BMC Genomics. 2017 Jun 8;18:456. doi: 10.1186/s12864-017-3833-0 (PMC5465549; doi:10.1186/s12864-017-3833-0)
Supplement: Supplementary file 4 — Features of putative Arabidopsis and rice PAEs. The name, locus, accession number, length, MW, and pI are given. (PDF 57.7 kb) [file 12864_2017_3833_MOESM4_ESM.pdf]

Additional file 4.

SemoePAE7	....
PhpatPAE1	..........YEGVQRLVKLLPTDGFYSGAKHPTNVLTPISSAQSEVIPRQEIPAGSDAS
SemoePAE6	....................SAFELTPDVPPIQHHH........................
BradiPAE11	....
SbPAE11	....
GRMZMPAE1.3	....
GRMZMPAE1.6	....
MedtrPAE6	....
BradiPAE10	....
SbPAE10	....
OsPAE6	....
MedtrPAE12	....
LasatPAE1	....
AtPAE8	....
TheccPAE4	....
PotriPAE4	....
RicomPAE1	....
LichiPAE1	....
MedtrPAE11	....
VradiPAE1	....
AtPAE7	....
AtPAE11	....
TheccPAE5	....
PotriPAE3	....
PotriPAE2	....
BradiPAE9	....
GRMZMPAE12.2	....
LusPAE2	....
AtPAE9	....
PotriPAE8	....
TheccPAE3	....
RicomPAE6	....
TheccPAE2.2	....
TheccPAE2.1	....
GRMZMPAE3.1	.................ASPAVEDELRGGGGAGGPT........................
BradiPAE3	....................AVNEQANGGGRRRRRS........................
SbPAE4.1	.................AADEEMNSSSNRGSRSRRR....................RRSR
SbPAE4.2	.................AADEEMNSSSNRGSRSRRR....................RRSR
BradiPAE1	..................SEQWSNETQVYATNGNS.........................
OsPAE9	..................SEPWLNQTQVYSTNANS.........................
GRMZMPAE8.1	..................SEPWWNETQVYATTANSG........................
GRMZMPAE8.2	..................SEPWWNETQVYATTANSG........................
SbPAE2	..................SEPWWNETQVYTTTANSG........................
AtPAE1	..................EMKTFNESNGT...............................
AtPAE2	MLLSHSRSPYKPRYVIKSKTSMIKKCKMKKLLWSWIILFNIHVNGMMMEFDEMEWFTVFN
MedtrPAE5	.................VDVELKGLENGNVTDMQYV........................
AtPAE3	..................SGNVRDTDDEISLLESQL........................
AtPAE6	.............VVQSGSSDGFGKPRDTETAISFL........................
AlPAE8	.............VIRSGSSDGFGKPIDTDTAISLL........................
RicomPAE5	..................NGLELNETEPEILYTGVA........................
AtPAE12	.................NEYLDFNVTEIDRIEELEF........................
AtPAE10	.................NGYLEFNVTELDRIEDLEF........................
MedtrPAE10	.................QQHHFFNETEELFLLEAHE........................
MedtrPAE1	.................NAYYHINETELSILEAHEA........................
MedtrPAE2	...........................GSDTTLSFS........................
MedtrPAE9.1	..................QTQNLSPRYILENDNDVV........................
MedtrPAE9.2	..................QTQNLSPRYILENDNDVV........................
TheccPAE1	...................EDAVEELKAKESLLSYL........................
RicomPAE4	.................GSVDVILKSEVMLPFLESG........................
BradiPAE2	....
GRMZMPAE4.1	....
OsPAE3	....
OsPAE2	....
OsPAE4	....
AtPAE4	...........MVIRSLLQCRTWSKSDWLLASIGIV..................LIVYSF
AtPAE5	........MAIPRFSSLLRCRKWAKSDWLVASIGCV...........LIVFFLSFFFDPT
OsPAE5	....
SbPAE9	....
SbPAE12	....
GRMZMPAE10.2	....
BradiPAE5	....
OsPAE8	....

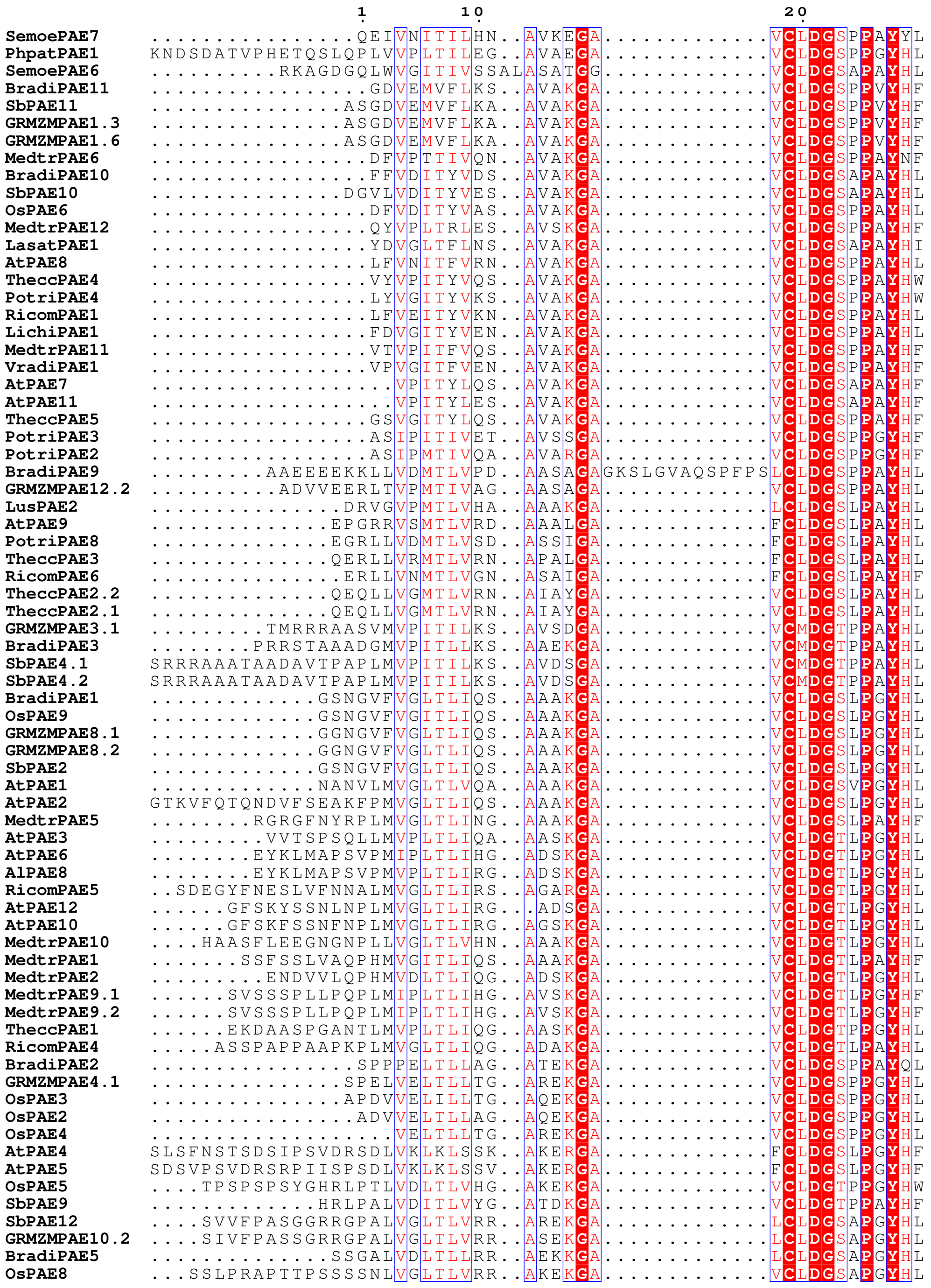
1 10 20
SemoePAE7 ................QEIVNITILHN..AVKEGA.............VCLDGSPPAYYL
PhpatPAE1 KNDSDATVPHETQSLQPLVVPLTILEG..AVAEGA.............VCLDGSPPGYHL
SemoePAE6 ..........RKAGDGQLWVGITIVSSALASATGG.............VCLDGSAPAYHL
BradiPAE11 .................GDVEMVFLKS..AVAKGA.............VCLDGSPPVYHF
SbPAE11 ...............ASGDVEMVFLKA..AVAKGA.............VCLDGSPPVYHF
GRMZMPAE1.3 ...............ASGDVEMVFLKA..AVAKGA.............VCLDGSPPVYHF
GRMZMPAE1.6 ...............ASGDVEMVFLKA..AVAKGA.............VCLDGSPPVYHF
MedtrPAE6 .................DFVPTTIVQN..AVAKGA.............VCLDGSPPAYNF
BradiPAE10 .................FFVDITYVDS..AVAKGA.............VCLDGSAPAYHL
SbPAE10 ...............DGVLVDITYVES..AVAKGA.............VCLDGSAPAYHL
OsPAE6 .................DFVDITYVAS..AVAKGA.............VCLDGSPPAYHL
MedtrPAE12 .................QYVPLTRLES..AVSKGA.............VCLDGSPPAYHF
LasatPAE1 .................YDVGLTFLNS..AVAKGA.............VCLDGSAPAYHI
AtPAE8 .................LFVNITFVRN..AVAKGA.............VCLDGSPPAYHL
TheccPAE4 .................VYVPITYVQS..AVAKGA.............VCLDGSPPAYHW
PotriPAE4 .................LYVGITYVKS..AVAKGA.............VCLDGSPPAYHW
RicomPAE1 .................LFVEITYVKN..AVAKGA.............VCLDGSPPAYHL
LichiPAE1 .................FDVGITYVEN..AVAKGA.............VCLDGSPPAYHL
MedtrPAE11 .................VTVPITFVQS..AVAKGA.............VCLDGSPPAYHF
VradiPAE1 .................VPVGITFVEN..AVAKGA.............VCLDGSPPAYHF
AtPAE7 ...................VPITYLQS..AVAKGA.............VCLDGSAPAYHF
AtPAE11 ...................VPITYLES..AVAKGA.............VCLDGSAPAYHF
TheccPAE5 .................GSVGITYLQS..AVAKGA.............VCLDGSPPAYHF
PotriPAE3 .................ASIPITIVET..AVSSGA.............VCLDGSPPGYHF
PotriPAE2 .................ASIPMTIVQA..AVARGA.............VCLDGSPPGYHF
BradiPAE9 .........AAEEEEKKLLVDMTLVPD..AASAGAGKSLGVAQSPFPSLCLDGSPPAYHL
GRMZMPAE12.2 ..........ADVVEERLTVPMTIVAG..AASAGA.............VCLDGSPPAYHL
LusPAE2 ...............DRVGVPMTLVHA..AAAKGA.............LCLDGSLPAYHL
AtPAE9 ..............EPGRRVSMTLVRD..AAALGA.............FCLDGSLPAYHL
PotriPAE8 ..............EGRLLVDMTLVSD..ASSIGA.............FCLDGSLPAYHL
TheccPAE3 ..............QERLLVRMTLVRN..APALGA.............FCLDGSLPAYHL
RicomPAE6 ...............ERLLVNMTLVGN..ASAIGA.............FCLDGSLPAYHF
TheccPAE2.2 ..............QEQLLVGMTLVRN..AIAYGA.............VCLDGSLPAYHL
TheccPAE2.1 ..............QEQLLVGMTLVRN..AIAYGA.............VCLDGSLPAYHL
GRMZMPAE3.1 .........TMRRRAASVMVPITILKS..AVSDGA.............VCMDGTPPAYHL
BradiPAE3 ........PRRSTAAADGMVPITLLKS..AAEKGA.............VCMDGTPPAYHL
SbPAE4.1 SRRRAAATAADAVTPAPLMVPITILKS..AVDSGA.............VCMDGTPPAYHL
SbPAE4.2 SRRRAAATAADAVTPAPLMVPITILKS..AVDSGA.............VCMDGTPPAYHL
BradiPAE1 .............GSNGVFVGLTLIQS..AAAKGA.............VCLDGSLPGYHL
OsPAE9 .............GSNGVFVGITLIQS..AAAKGA.............VCLDGSLPGYHL
GRMZMPAE8.1 .............GGNGVFVGLTLIQS..AAAKGA.............VCLDGSLPGYHL
GRMZMPAE8.2 .............GGNGVFVGLTLIQS..AAAKGA.............VCLDGSLPGYHL
SbPAE2 .............GSNGVFVGLTLIQS..AAAKGA.............VCLDGSLPGYHL
AtPAE1 .............NANVLMVGLTLVQA..AAAKGA.............VCLDGSVPGYHL
AtPAE2 GTKVFQTQNDVFSEAKFPMVGLTLIQS..AAAKGA.............VCLDGSLPGYHL
MedtrPAE5 ........RGRGFNYRPLMVGLTLING..AAAKGA.............VCLDGSLPAYHF
AtPAE3 .........VVTSPSQLLMVPLTLIQA..AASKGA.............VCLDGTLPGYHL
AtPAE6 ........EYKLMAPSVPMIPLTLIHG..ADSKGA.............VCLDGTLPGYHL
AlPAE8 ........EYKLMAPSVPMVPLTLIRG..ADSKGA.............VCLDGTLPGYHL
RicomPAE5 ..SDEGYFNESLVFNNALMVGLTLIRS..AGARGA.............VCLDGTLPGYHL
AtPAE12 ......GFSKYSSNLNPLMVGLTLIRG...ADSGA.............VCLDGTLPGYHL
AtPAE10 ......GFSKFSSNFNPLMVGLTLIRG..AGSKGA.............VCLDGTLPGYHL
MedtrPAE10 ....HAASFLEEGNGNPLLVGLTLVHN..AAAKGA.............VCLDGTLPGYHL
MedtrPAE1 .......SSFSSLVAQPHMVGITLIQS..AAAKGA.............VCLDGTLPAYHF
MedtrPAE2 .........ENDVVLQPHMVDLTLIQG..ADSKGA.............VCLDGTLPGYHL
MedtrPAE9.1 ......SVSSSPLLPQPLMIPLTLIHG..AVSKGA.............VCLDGTLPGYHF
MedtrPAE9.2 ......SVSSSPLLPQPLMIPLTLIHG..AVSKGA.............VCLDGTLPGYHF
TheccPAE1 ......EKDAASPGANTLMVPLTLIQG..AASKGA.............VCLDGTPPGYHL
RicomPAE4 .....ASSPAPPAAPKPLMVGLTLIQG..ADAKGA.............VCLDGTLPAYHL
BradiPAE2 ................SPPPELTLLAG..ATEKGA.............VCLDGSPPAYQL
GRMZMPAE4.1 ...............SPELVELTLLTG..AREKGA.............VCLDGSPPGYHL
OsPAE3 ...............APDVVELILLTG..AQEKGA.............VCLDGSPPGYHL
OsPAE2 ................ADVVELTLLAG..AQEKGA.............VCLDGSPPGYHL
OsPAE4 ...................VELTLLTG..AREKGA.............VCLDGSPPGYHL
AtPAE4 SLSFNSTSDSIPSVDRSDLVKLKLSSK..AKERGA.............FCLDGSLPGYHF
AtPAE5 SDSVPSVDRSRPIISPSDLVKLKLSSV..AKERGA.............FCLDGSLPGYHF
OsPAE5 ....TPSPSPSYGHRLPTLVDLTLVHG..AKEKGA.............VCLDGTPPGYHW
SbPAE9 .............HRLPALVDITLVYG..ATDKGA.............VCLDGTPPAYHF
SbPAE12 ....SVVFPASGGRRGPALVGLTLVRR..AREKGA.............LCLDGSAPGYHL
GRMZMPAE10.2 ....SIVFPASSGRRGPALVGLTLVRR..ASEKGA.............LCLDGSAPGYHL
BradiPAE5 ..............SSGALVDLTLLRR..AEKKGA.............LCLDGSAPGYHL
OsPAE8 ...SSLPRAPTTPSSSSNLVGLTLVRR..AKEKGA.............VCLDGSAPGYHL

```
             30        40        50        60        70        80
SemoePAE7    RR...RNSPNWLLFLRGGVCYGDSK.ERSCLSRSTSELGSSQQMSEQISLNFGILSISK
PhpatPAE1    HEGNGGNARNWVLFLEEGAWCES....EAACKVRARAHLGSSKWMNDR.TFE.GILSNSE
SemoePAE6    LKGSGSGANSWHLHLEGGAWCES....IEKCVERASTNLGSSSKMETSIPFT.GLLNNNY
BradiPAE11   SPGSGSGANNWVVHMEGGGWCKT....SEECSIRKGNFRGSSKYM.KPLSFS.GILGGSD
SbPAE11      SPGSGSGADNWV....GGGWCRN....PDECAVRKGNFRGSSKFM.RPLSFS.GILGGSQ
GRMZMPAE1.3  SPGSGSGANNWVVHMEGGGWCRN....PDECAVRKGNFRGSSKFM.KPLSFS.GILGGNQ
GRMZMPAE1.6  SPGSGSGANNWVVHMEGGGWCRN....PDECAVRKGNFRGSSKFM.KPLSFS.GILGGNQ
MedtrPAE6    DKGYGKGSNSWLVHMEGGGWCHN....VTNCLGRMTTRLGSSKQMNKTLAFS.GILNDKK
BradiPAE10   ARGFGSGVDSWLVHFEGGGWCSN....VTTCLERKNTRLGSSKEMAKQVAFS.GILSNTP
SbPAE10      ARGSGSGENSWLVHFEGGGWCNN....VTTCLQRKRTRLGSSKEMATQIAFS.GILSDTP
OsPAE6       ARGFGSGVNSWLVHFEGGGWCSN....VTTCLQRKRTRLGSSKQMAKQIAFS.GILSNTP
MedtrPAE12   DQGHDEGANNWIVHIEGGGWCHN....VTYCLYRRDSRLGSSHEMEEQTYFS.GYLSDNQ
LasatPAE1    DKGFGAGIDNWLVFFEGGGWCNN....ATNCLTRRDTRLGSSKKMLTTETFS.GMFHNKA
AtPAE8       DRGSGTGINSWLIQLEGGGWCNN....VTNCVSRMHTRLGSSKKMVENLAFS.AILSNKK
TheccPAE4    DRGYGTGINSWLIQLEGGGWCNN....VTSCLVRKNTHLGSSKRMVKQIPFS.GILNNKH
PotriPAE4    DKGFGTGINSWLIHFEGGGWCNN....VTSCLSRKKTHLGSSKLMGQQIAFS.GIMNNKR
RicomPAE1    DKGFGTGINSWLVHFEGGGWCNN....ITTCLSRKNTRLGSSKQMGPQIAFS.GIMSNRR
LichiPAE1    DKGFGAGINNWLVHIEGGGWCNN....VTTCLSRKNTRLGSSKKMVKVVAFS.GMLSNKQ
MedtrPAE11   DKGFEAGIDNWIVHFEGGGWCNN....ATTCLDRIDTRLGSSKKMDKTLSFS.GFFSSGK
VradiPAE1    HKGSGAGINNWIVHFEGGGWCNN....VTSCLSRRDTRLGSSKKMDTQLSFS.GFFSNSK
AtPAE7       DKGFGSGVNNWIVHMEGGGWCTD....VASCNERKGTMKGSSKFMNKDFGFS.GILGGKQ
AtPAE11      DKGSGSGVNNWIVHMEGGGWCTD....IATCVQRKSTMKGSSKLMNKDFGFS.GILGGKQ
TheccPAE5    DPGSGAGVNNWLVHMEGGGWCED....VERCLSRRNTDLGSSEQMVKQFGFS.GLLSSQQ
PotriPAE3    DKGSGSGIDNWLVHMEGGGWCES....VESCVSRRDTFRGSSSKMEKTMGFS.GILGSKQ
PotriPAE2    EKGSGSGINNWLVHMEGGGWCES....VESCVSRRDTYKGSSLKMEKTMGFS.GILGSKQ
BradiPAE9    DRGAGSGA.GWLLQFEGGGWCND....ARSCAERAGTRRGSTRLMNKLEVFS.GVLSDDP
GRMZMPAE12.2 HGGSGAGARSWLLQFEGGGWCND....VRSCAERAGTRRGSTRLMAKAESFS.GILSNRP
LusPAE2      QRGFGAGANNWLLQFEGGGWCNT....VDSCWERAKTRRGSTSLMVKLENFS.GILSNNA
AtPAE9       DRGFGAGSNNWILQFEGGGWCND....IASCVERAKTRRGSTRYMSKTVVFT.GVLSNNA
PotriPAE8    HRGFGAGASNWLLQFEGGGWCND....IQSCLDRAKTKHGSSLYMNKLEDFN.GILSNDA
TheccPAE3    HRGFGNGANNWILFFEGGGWCGH....REQCLDRAKTSYGSTNHMNKWAIFS.GILSNNA
RicomPAE6    HRGSGTGARNWLLQFEGGGWCND....LQSCLERAKTRRGSTRYMNKLETFS.GILSNNA
TheccPAE2.2  HRGFGAGANNWILQFEGGGWCND....IPSCLERANTRRGSTRYMNKFEVFS.GILSDND
TheccPAE2.1  HRGFGAGANNWILQFEGGGWCND....IPSCLERANTRRGSTRYMNKFEVFS.GILSDND
GRMZMPAE3.1  DPGSGAGSRSWIVNLEGGAWCNS....AKTCRLTRSSGRGSSDHMDKEIPFT.GIMSSSR
BradiPAE3    DPGSGAGNNSWIVNLEGGGWCNN....ARTCKFRTRTRHGSSDYMERHITFS.GIMSASP
SbPAE4.1     HPGSGAGNNSWIVNLEGGGWCNN....VRACQFRKASRRGSSDLMEKEIPFG.GIMSSSP
SbPAE4.2     HPGSGAGNNSWIVNLEGGGWCNN....VRACQFRKASRRGSSDLMEKEIPFG.GIMSSSP
BradiPAE1    HRGFGSGSKNWLVNLEGGGWCND....VKSCVFRKSSRRGSSNHMEKQLQFT.GIMSNRP
OsPAE9       HRGFGSGANSWLVNLEGGGWCND....VKSCVFRKSSRRGSSNHMESQLQFT.GIMSNRP
GRMZMPAE8.1  HRGFGSGANSWLVNLEGGGWCND....RSSCVFRKGSRRGSSNHMERQLQFT.GILSNKP
GRMZMPAE8.2  HRGFGSGANSWLVNLEGGGWCND....RSSCVFRKGSRRGSSNHMERQLQFT.GILSNKP
SbPAE2       HRGFGSGANSWLVNLEGGGWCND....VSSCVFRKGSRRGSSNHMERQLQFT.GIMSNRP
AtPAE1       CRGYGSGANNWIIQLQGGAWCDS....IQNCQSRKGSGYGSSTLMEKELAFL.GLLSNKA
AtPAE2       HRGFGSGANNWLVQLEGGGWCDT....IRNCVYRKTTRRGSSSYMEKEIPFT.GILSDKA
MedtrPAE5    HRGYGSGSNSWLIHLEGGGWCGT....VRNCIYSKKTRHGSSYFMEKQIPFI.GILSNKA
AtPAE3       HPGSGSGANRWLIQLEGGGWCNT....RRSCIFRKTTRRGSSNHMEKVLAFT.GILSNKS
AtPAE6       DRGFGSGANSWLIQLEGGGWCNN....HRSCVYRKTSRRGSSKFMEKALAFT.GILSNRS
AlPAE8       DRGFGSGANSWLIQLEGGGWCNN....HRSCVYRKTSRRGSSKFMEKALAFT.GILSNKS
RicomPAE5    HRGYGSGANSWLIQLEGGGWCNN....IRNCVYRKKTRRGSSKYMEKQLAFT.GILSNKP
AtPAE12      HRGHGSGANSWLIQLEGGGWCNN....IRTCVYRKTTRRGSSNYMEKQLQFT.GILSDKA
AtPAE10      HRGHGSGANSWLIQLEGGGWCDN....IRNCVYRKKSRRGSSNYMEKQIQFT.GILSNKA
MedtrPAE10   HRGYGSGADSWLVNLEGGGWCNN....VRSCVYRKKTRRGSSLYMEKEIPFT.GILSNKP
MedtrPAE1    DHGYGSGANSWLVNLEGGGWCNN....RRTCVYRKTTRRGSSKFMEKAIPFT.......
MedtrPAE2    DRGFESGANSWLIHLEGGGWCDT....VRNCVYRKKTHRGSSKYMENQIPFT.GILSNKP
MedtrPAE9.1  HPGSGSGANSWLIQLEGGGWCNT....IRSCVFRKTTRRGSSKYMEKQLPFT.GILSNKA
MedtrPAE9.2  HPGSGSGANSWLIQLEGGGWCNT....IRSCVFRKTTRRGSSKYMEKQLPFT.GILSNKA
TheccPAE1    DRGFGSGSNSWLIHLEGGGWCNN....IRTCVFRKTTRRGSSKFMAKQTNFT.GILSNKA
RicomPAE4    HRGSGSGQNSWLIQLEGGGWCNT....IRSCVYRKTTRRGSSKFMEKVLPFT.GILSNKP
BradiPAE2    DRGFGSGRYNWLVYLEGGGWCDT....IESCSKHKKSGLGSSNLIE.AVQLP.GIFSNDH
GRMZMPAE4.1  QRGFGSGSHSWIVYLQGGAWCSSNTTDTETCSERKMTAYGSSKLM.GAVTFD.GIFRNQQ
OsPAE3       QRGFGSGEHSWLIYLEGGEWCDT....IESCSNRKTTELGSSKLMEAQ.EFE.GILSNNQ
OsPAE2       QRGFGSGEHSWLVFLEGGAWCNS....IESCSRRKMGVYGSSKFM.KAAEFN.GILSNDQ
OsPAE4       QRGFGSGEHSWFIHLQGGAWCNT....IEDCSKRKMSAYGSSKFM.RAVEFN.GILSNDQ
AtPAE4       HKGSGSGSNSWLLYLEGGGGCRT....IESCSARAMTRLGSSNFFEHEVPFF.GVLSSDP
AtPAE5       HEGSGSGSQSWLVHLEGGGWCNT....VASCSARALTKLGSSNYFEQEVAFQ.GVLSSDP
OsPAE5       LPGFGDGSNKWLLHLEGGSWCRN....RTSCDHRKKTSLGSSAYMETRVEFV.GILSDDK
SbPAE9       LPGFGDGSHNWLLHLEGGSWCRS....FESCARRKKTNLGSSAHMDTRAEFV.GILSDDQ
SbPAE12      QRGSGSGSQSWLIHLEGGGWCRN....LKSCASRQRSMLGSSRYMEGQVEFT.GILSDDR
GRMZMPAE10.2 QGGSGSGSRSWLIHLEGGGWCRN....LKSCASRQRSMLGSSRYMEGQVEFT.GILSDDK
BradiPAE5    QRGSGSGSQSWLIHLEGGGWCRN....LKSCASRQKSMLGSSRYMERQVEFA.GILSDDE
OsPAE8       QRGSGTGSQNWLLHLEGGGWCRN....LRSCASRQKSVLGSSQYMECQIEFA.GILSNDK
```

```
                     90        100       110       120       130       140
SemoePAE7     KNNPDFWNWN.HVEITYCDGGSYLGDVEKPVQVFDTETNKTRYLYYRGRKIWNYTIRNLL
PhpatPAE1     EVNPDFYNWN.RVFVRYCDGASFSGNSS......LPTKTEGNALHYRGESIWNFVIDDLL
SemoePAE6     NVNPDFYNWN.HVYVRYCDGSSFNSDVA.....NPYKTSSGQTLYFRGRKAFKAIIDDLK
BradiPAE11    KFNPDFYNWN.RVKVRYCDGSSFTGDVE........AVETSTNLHYRGNRVWQAIIQDLL
SbPAE11       KSNPDFYNWN.RIKVRYCDGSSFTGDVE........AVDTAKNLHYRGFRVWRAIMDDLL
GRMZMPAE1.3   KSNPDFYNWN.RVKIRYCDGSSFTGDVE........AVDTAKDLRYRGFRVWRAVIDDLL
GRMZMPAE1.6   KSNPDFYNWN.RVKIRYCDGSSFTGDVE........AVDTAKDLRYRGFRVWRAVIDDLL
MedtrPAE6     QFNPDFYNWN.RIKIRYCDGSSFTGDVE........AVDPVTKLHFRGARIFEAVMEELL
BradiPAE10    DHNPDFYNWN.KVRVRYCDGSSFTGDKE........EVDPTTKLHYRGARVWQAVMEDLL
SbPAE10       DGNPDFYNWN.KVKVRYCDGSSFTGDVE........EVDPTTKLHFRGARIWQAVMEDLL
OsPAE6        DYNPDFYNWN.KVKVRYCDGSSFTGDVE........KVDPATKLHYRGARVWQAVMDDLL
MedtrPAE12    QYNPDFYNWN.RVKVRYCDGSSFTGDVE........EVDPTTKLYYRGARIFSAVMEELL
LasatPAE1     KYNPDFYDWN.RIKVRYCDGASFTGDVE........AVDPNTKLYYRGARIFRAVVDDLL
AtPAE8        QYNPDFYNWN.RVKVRYCDGASFTGDVE........AVNPATNLHFRGARVWLAVMQELL
TheccPAE4     QFNPDFYNWN.RVKVRYCDGSSFTGDVA........AVNPVTNLHFRGARVWLAVMEDLL
PotriPAE4     PFNPDFYNWN.RVKIRYCDGSSFTGDVQ........AVNPATNLHFRGARIWLAVIEDLL
RicomPAE1     RYNPDFYNWN.RIKVRYCDGSSFTGDVQ........AVNPATNLHFRGARIWLAVIEELL
LichiPAE1     KFNPDFYNWN.RIKVRYCDGASFTGDVE........AVNPATNLHFRGARVFLAIIEDLL
MedtrPAE11    KFNPDFYNWN.RIKVRYCDGSSFTGDVE........AVDPKTNLHYRGGRIFVAVIEDLL
VradiPAE1     KFNPDFYDWN.RIKVRYCDGSSFTGDVE........AVDPATNLHFRGSRVFAAVVDDLL
AtPAE7        STNPDFYNWN.RIKVRYCDGSSFTGNVE........AVNPANKLFFRGARVWRAVVDDLM
AtPAE11       STNPDFYNWN.RIKVRYCDGSSFTGDIE........AVDPTHKLFFRGARVWRAVIDDLM
TheccPAE5     KSNPDFYNWN.RIKVRYCDGSSFTGDVE..........APVNNLFFRGNRIWEAIIEDLL
PotriPAE3     AANPDFYNWN.RIKVRYCDGSSFTGDVE........AVDPE.KLYFRGERVWQAVIDELL
PotriPAE2     AANPDFYNWN.RIKIRYCDGSSFTGDVE........AVDPKTKLYFRGERIWQAVIDDLL
BradiPAE9     AKNPDFYNWN.RVKLRYCDGGSFAGDSE........FINGTSIIYMRGQRIWDAIITDLF
GRMZMPAE12.2  AMNPDFYNWN.RVKLRYCDGGSFMGDSA.......VYINSSSVLYFSGQRIWDAIVADLL
LusPAE2       SLNPDFYNWN.RVKLRYCDGASFTGDSK........IVNGSSVLYFRGQRIWDAIITDLL
AtPAE9        SQNPDFYNWN.KVRLRYCDGASFAGDSQ........FGNGTSLLYFRGQRIWNAIILDLL
PotriPAE8     SLNPDFYNWN.RVKLRYCDGGSFSGDAK........FDNGTSVLYFRGQKIWEAMILDLL
TheccPAE3     SLNPDFYNWN.RVRLRYCDGASFAGDGK........FANGTSLLYFRGQKIWEAIILDLL
RicomPAE6     SLNPDFYNWN.RVKLRYCDGASFAGDAK........FDNGTSVLYFRGQRIWQAIIRDLL
TheccPAE2.2   SLNPDFYNWN.RVKLRYCDGASFSGDSK........FDNGTSLLYFRGQKIWEAIIRDLL
TheccPAE2.1   SLNPDFYNWN.RVKLRYCDGASFSGDSK........FDNGTSLLYFRGQKIWEAIIRDLL
GRMZMPAE3.1   AVNPDFYNWN.RVKVRYCDGGSFAGEAF..........DKDTGIYFRGQRIWNAVIRHLL
BradiPAE3     ASNPDFYSWN.RVKIRYCDSASFAGDNF..........DKGTGLYFRGQRIWDAAIQHLL
SbPAE4.1      VDNPDFYKWN.RVKIRYCDGASFAGEGF..........DKENGFYFRGQRIWDATVRHLL
SbPAE4.2      VDNPDFYKWN.RVKIRYCDGASFAGEGF..........DKENGFYFRGQRIWDATVRHLL
BradiPAE1     EENPDFYNWN.RVKVRYCDGGSFTGDGA..........DAASGLYFRGQRIWQAAMDDLM
OsPAE9        EENPDFYNWN.RVKVRYCDGGSFTGDGA..........DASAGLYFRGQRIWQAAMDDLM
GRMZMPAE8.1   EENPDFYNWN.RVKVRYCDGGSFTGDGS..........DAAAGLYFRGQRIWQAAMDDLM
GRMZMPAE8.2   EENPDFYNWN.RVKVRYCDGGSFTGDGS..........DAAAGLYFRGQRIWQAAMDDLM
SbPAE2        DENPDFYNWN.RVKVRYCDGGSFTGDGS..........DAAAGLYFRGQRIWQAAMDDLM
AtPAE1        AENPDFYNWN.KVKVRYCDGASFDGDSE..........NKAAQLQYRGKRIFLAVMEDLM
AtPAE2        ADNPDFYNWN.RVKVRYCDGGSFSGDSE..........NKAAQLQFRGKRIWLAAMEDLM
MedtrPAE5     AENPDFFNWN.RVKIRYCDGASFSGDSQ..........NEAARLYFRGQRIWQAAMEDLM
AtPAE3        NENPDFFNWN.RVKLRYCDGASFTGDSQ..........DESSQLYYRGQRIWHSAMEELL
AtPAE6        EENPDFFNWN.RIKLRYCDGASFSGDSQ..........DESSQLFYRGQRIWQVAMEEFL
AlPAE8        EENPDFFNWN.RIKLRYCDGASFSGDSQ..........DESSQLFYRGQRIWQVAMEEFL
RicomPAE5     QENPDFFNWN.RVKLRYCDGASFSGDNE..........NKAAQLQFRGQRIWLAAMQDLM
AtPAE12       QENPDFFNWN.RVKLRYCDGASFSGDGQ..........NQAAQLQFRGERIWRAAIDDLK
AtPAE10       QENPDFFNWN.RVKLRYCDGASFSGDSQ..........NKAARLQFRGEKIWRAAMDDLK
MedtrPAE10    EENPDFFNWN.RAKLRYCDGGSFAGDGE..........DQDAELQFRGQRIWAAAMEDLI
MedtrPAE1     ........WNIKVKIRYCDGASFTGDSE..........DKAAQLQFRGQRIWLAAVEDLM
MedtrPAE2     EENPDFFNWN.RVKLRYCDGASFAGDSE..........NEAAGLQFRGQKIWLAAIEELL
MedtrPAE9.1   EQNPDFFNWN.RVKVRYCDGASFSGDSQ..........NEAAQLQFRGQKIWLAAMEELM
MedtrPAE9.2   EQNPDFFNWN.RVKVRYCDGASFSGDSQ..........NEAAQLQFRGQKIWLAAMEELM
TheccPAE1     EENPDFFNWN.RVKLRYCDGASFAGEGQ..........NEANQLYFRGQRIWSAAMEELM
RicomPAE4     DENPDFFNWN.RVKLRYCDGASFSGDYH..........DEAAQLYFRGQRIWSAAMENLM
BradiPAE2     RQNSDFYNWN.KVFIRYCDGASFSGDAE.......GEDQDGTKLFFRGLRIWEAVIDELM
GRMZMPAE4.1   PQNPDFYNWN.KVYVRYCDGASFSGDAE.......GEAQDGTKLFFRGSRIWDAVVDELM
OsPAE3        TVNSDFYNWN.KVVIRYCDGASFSGNAE.......AQDQDGSTLHFRGLRIWQAVLDELM
OsPAE2        QLNSDFYNWN.KVAIRYCDGASFSGDAE.......AQDKDGSTLHFRGLRIWEAVVDELM
OsPAE4        QLNSDFYNWN.RVFIRYCDGASFSGDGE.......AQDQDGSTLHFRGLRIWEAVINELM
AtPAE4        SQNPDFFNWN.RVMIRYCDGACFSGHPE.......AEFKNETRLFFRGQLIWEAIMDELL
AtPAE5        SQNPEFFNWN.KVAIRYCDGASFSGRPE.......AEFKNGTRLFFRGQLIWEAIIDELL
OsPAE5        AQNPDFYNWN.KVKIRYCDGASFTGDVQ.......DEHQYGATFFFRGQRIWEAVMAELL
SbPAE9        SQNPDFYNWN.KVKIRYCDGASFSGHVQ.......DEVKNGTGFFFRGQRIWEAVMAELL
SbPAE12       SQNPDFYNWN.KVKIRYCDGASFSGNVK.......DELQNGTRFFFRGQRIWEAVMNELV
GRMZMPAE10.2  SQNPDFYNWN.KVKIRYCDGASFSGDVK.......DELQNGTRFFFRGQRIWEAVMNELV
BradiPAE5     AQNPDFYDWN.KVKIRYCDGASFSGNVK.......DEFQNGTKFFFRGQRIWKAVMDELL
OsPAE8        FQNPDFYNWN.KVKIRYCDGASFSGNVK.......NELQNGTKFFFRGQRIWEAVMSELL
```

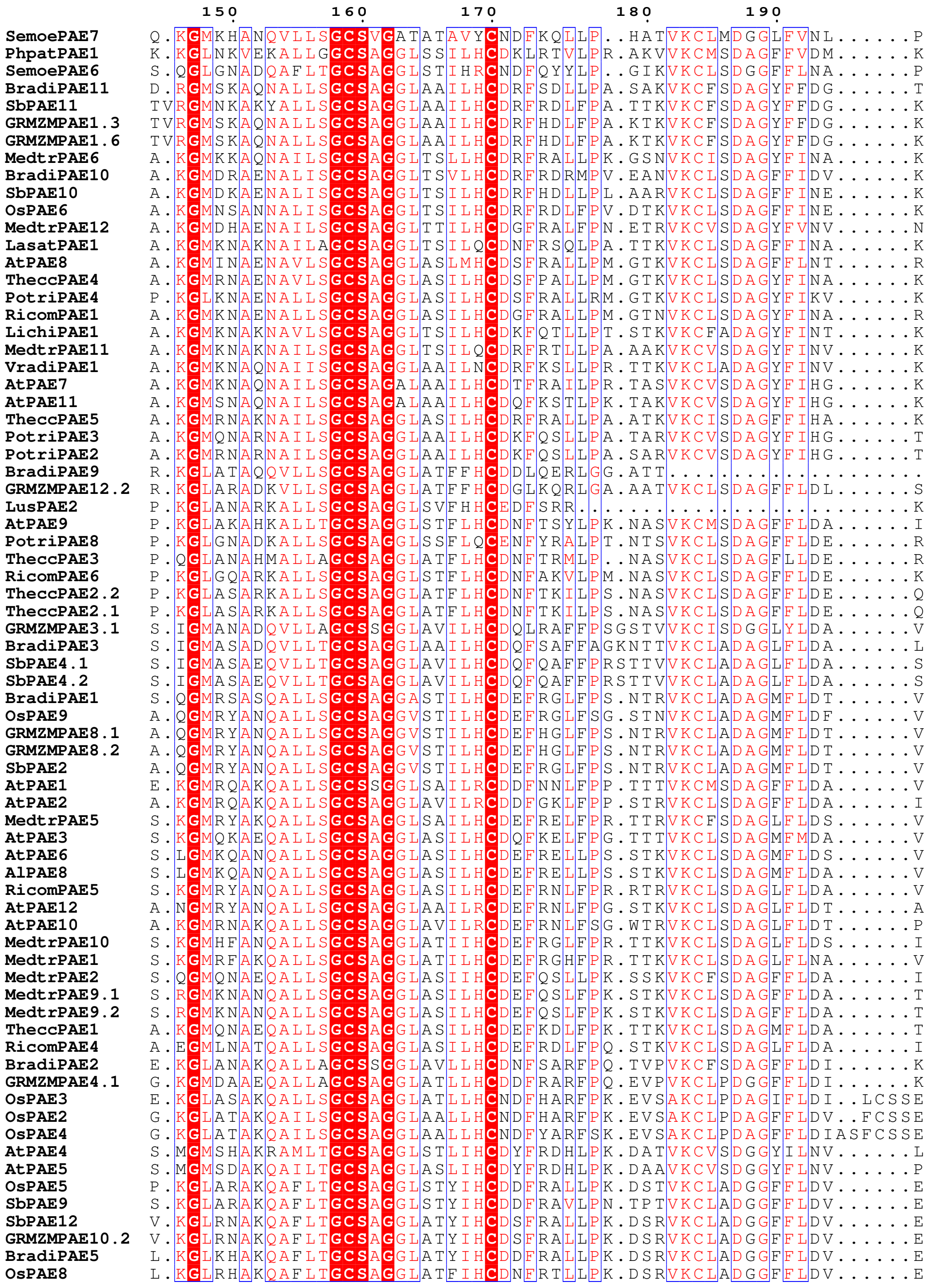

150 160 170 180 190
SemoePAE7 Q.KGMKHANQVLLSGCSVGATATAVYCNDFKQLLP..HATVKCLMDGGLFVNL......P
PhpatPAE1 K.KGLNKVEKALLGGCSAGGLSSILHCDKLRTVLPR.AKVVKCMSDAGFFVDM......K
SemoePAE6 S.QGLGNADQAFLTGCSAGGLSTIHRCNDFQYYLP..GIKVKCLSDGGFFLNA......P
BradiPAE11 D.RGMSKAQNALLSGCSAGGLAAILHCDRFSDLLPA.SAKVKCFSDAGYFFDG......T
SbPAE11 TVRGMNKAKYALLSGCSAGGLAAILHCDRFRDLFPA.TTKVKCFSDAGYFFDG......K
GRMZMPAE1.3 TVRGMSKAQNALLSGCSAGGLAAILHCDRFHDLFPA.KTKVKCFSDAGYFFDG......K
GRMZMPAE1.6 TVRGMSKAQNALLSGCSAGGLAAILHCDRFHDLFPA.KTKVKCFSDAGYFFDG......K
MedtrPAE6 A.KGMKKAQNAILSGCSAGGLTSLLHCDRFRALLPK.GSNVKCISDAGYFINA......K
BradiPAE10 A.KGMDRAENALISGCSAGGLTSVLHCDRFRDRMPV.EANVKCLSDAGFFIDV......K
SbPAE10 A.KGMDKAENALISGCSAGGLTSILHCDRFHDLLPL.AARVKCLSDAGFFINE......K
OsPAE6 A.KGMNSANNALISGCSAGGLTSILHCDRFRDLFPV.DTKVKCLSDAGFFINE......K
MedtrPAE12 A.KGMDHAENAILSGCSAGGLTTILHCDGFRALFPN.ETRVKCVSDAGYFVNV......N
LasatPAE1 A.KGMKNAKNAILAGCSAGGLTSILQCDNFRSQLPA.TTKVKCLSDAGFFINA......K
AtPAE8 A.KGMINAENAVLSGCSAGGLASLMHCDSFRALLPM.GTKVKCLSDAGFFLNT......R
TheccPAE4 A.KGMRNAENAVLSGCSAGGLASILHCDSFPALLPM.GTKVKCLSDAGYFINA......K
PotriPAE4 P.KGLKNAENALLSGCSAGGLASILHCDSFRALLRM.GTKVKCLSDAGYFIKV......K
RicomPAE1 A.KGMKNAENALLSGCSAGGLASILHCDGFRALLPM.GTNVKCLSDAGYFINA......R
LichiPAE1 A.KGMKNAKNAVLSGCSAGGLTSILHCDKFQTLLPT.STKVKCFADAGYFINT......K
MedtrPAE11 A.KGMKNAKNAILSGCSAGGLTSILQCDRFRTLLPA.AAKVKCVSDAGYFINV......K
VradiPAE1 A.KGMKNAQNAIISGCSAGGLAAILNCDRFKSLLPR.TTKVKCLADAGYFINV......K
AtPAE7 A.KGMKNAQNAILSGCSAGALAAILHCDTFRAILPR.TASVKCVSDAGYFIHG......K
AtPAE11 A.KGMSNAQNAILSGCSAGALAAILHCDQFKSTLPK.TAKVKCVSDAGYFIHG......K
TheccPAE5 A.KGMRNAKNAILSGCSAGGLASILHCDRFRALLPA.ATKVKCISDAGFFIHA......K
PotriPAE3 A.KGMQNARNAILSGCSAGGLAAILHCDKFQSLLPA.TARVKCVSDAGYFIHG......T
PotriPAE2 A.KGMRNARNAILSGCSAGGLAAILHCDKFQSLLPA.SARVKCVSDAGYFIHG......T
BradiPAE9 R.KGLATAQQVLLSGCSAGGLATFFHCDDLQERLGG.ATT......................
GRMZMPAE12.2 R.KGLARADKVLLSGCSAGGLATFFHCDGLKQRLGA.AATVKCLSDAGFFLDL......S
LusPAE2 P.KGLANARKALLSGCSAGGLSVFHHCEDFSRR.........................K
AtPAE9 P.KGLAKAHKALLTGCSAGGLSTFLHCDNFTSYLPK.NASVKCMSDAGFFLDA......I
PotriPAE8 P.KGLGNADKALLSGCSAGGLSSFLQCENFYRALPT.NTSVKCLSDAGFFLDE......R
TheccPAE3 P.QGLANAHMALLAGCSAGGLATFLHCDNFTRMLP..NASVKCLSDAGFLLDE......R
RicomPAE6 P.KGLGQARKALLSGCSAGGLSTFLHCDNFAKVLPM.NASVKCLSDAGFFLDE......K
TheccPAE2.2 P.KGLASARKALLSGCSAGGLATFLHCDNFTKILPS.NASVKCLSDAGFFLDE......Q
TheccPAE2.1 P.KGLASARKALLSGCSAGGLATFLHCDNFTKILPS.NASVKCLSDAGFFLDE......Q
GRMZMPAE3.1 S.IGMANADQVLLAGCSSGGLAVILHCDQLRAFFPSGSTVVKCISDGGLYLDA......V
BradiPAE3 S.IGMASADQVLLTGCSAGGLAAILHCDQFSAFFAGKNTTVKCLADAGLFLDA......L
SbPAE4.1 S.IGMASAEQVLLTGCSAGGLAVILHCDQFQAFFPRSTTVVKCLADAGLFLDA......S
SbPAE4.2 S.IGMASAEQVLLTGCSAGGLAVILHCDQFQAFFPRSTTVVKCLADAGLFLDA......S
BradiPAE1 S.QGMRSASQALLSGCSAGGASTILHCDEFRGLFPS.NTRVKCLADAGMFLDT......V
OsPAE9 A.QGMRYANQALLSGCSAGGVSTILHCDEFRGLFSG.STNVKCLADAGMFLDF......V
GRMZMPAE8.1 A.QGMRYANQALLSGCSAGGVSTILHCDEFHGLFPS.NTRVKCLADAGMFLDT......V
GRMZMPAE8.2 A.QGMRYANQALLSGCSAGGVSTILHCDEFHGLFPS.NTRVKCLADAGMFLDT......V
SbPAE2 A.QGMRYANQALLSGCSAGGVSTILHCDEFRGLFPS.NTRVKCLADAGMFLDT......V
AtPAE1 E.KGMRQAKQALLSGCSSGGLSAILRCDDFNNLFPP.TTTVKCMSDAGFFLDA......V
AtPAE2 A.KGMRQAKQALLSGCSAGGLAVILRCDDFGKLFPP.STRVKCLSDAGFFLDA......I
MedtrPAE5 S.KGMRYAKQALLSGCSAGGLSAILHCDEFRELFPR.TTRVKCFSDAGLFLDS......V
AtPAE3 S.KGMQKAEQALLSGCSAGGLASILHCDQFKELFPG.TTTVKCLSDAGMFMDA......V
AtPAE6 S.LGMKQANQALLSGCSAGGLASILHCDEFRELLPS.STKVKCLSDAGMFLDS......V
AlPAE8 S.LGMKQANQALLSGCSAGGLASILHCDEFRELLPS.STKVKCLSDAGMFLDA......V
RicomPAE5 S.KGMRYANQALLSGCSAGGLASILHCDEFRNLFPR.RTRVKCLSDAGLFLDA......V
AtPAE12 A.NGMRYANQALLSGCSAGGLAAILRCDEFRNLFPG.STKVKCLSDAGLFLDT......A
AtPAE10 A.KGMRNAKQALLSGCSAGGLAVILRCDEFRNLFSG.WTRVKCLSDAGLFLDT......P
MedtrPAE10 S.KGMHFANQALLSGCSAGGLATIIHCDEFRGLFPR.TTKVKCLSDAGLFLDS......I
MedtrPAE1 S.KGMRFAKQALLSGCSAGGLATILHCDEFRGHFPR.TTKVKCLSDAGLFLNA......V
MedtrPAE2 S.QGMQNAEQALLSGCSAGGLASIIHCDEFQSLLPK.SSKVKCFSDAGFFLDA......I
MedtrPAE9.1 S.RGMKNANQALLSGCSAGGLASILHCDEFQSLFPK.STKVKCLSDAGFFLDA......T
MedtrPAE9.2 S.RGMKNANQALLSGCSAGGLASILHCDEFQSLFPK.STKVKCLSDAGFFLDA......T
TheccPAE1 A.KGMQNAEQALLSGCSAGGLASILHCDEFKDLFPK.TTKVKCLSDAGMFLDA......T
RicomPAE4 A.EGMLNATQALLSGCSAGGLASILHCDEFRDLFPQ.STKVKCLSDAGLFLDA......I
BradiPAE2 E.KGLANAKQALLAGCSSGGLAVLLHCDNFSARFPQ.TVPVKCFSDAGFFLDI......K
GRMZMPAE4.1 G.KGMDAAEQALLAGCSAGGLATLLHCDDFRARFPQ.EVPVKCLPDGGFFLDI......K
OsPAE3 E.KGLASAKQALLSGCSAGGLATLLHCNDFHARFPK.EVSAKCLPDAGIFLDI..LCSSE
OsPAE2 G.KGLATAKQAILSGCSAGGLAALLHCNDFHARFPK.EVSAKCLPDAGFFLDV..FCSSE
OsPAE4 G.KGLATAKQAILSGCSAGGLAALLHCNDFYARFSK.EVSAKCLPDAGFFLDIASFCSSE
AtPAE4 S.MGMSHAKRAMLTGCSAGGLSTLIHCDYFRDHLPK.DATVKCVSDGGYILNV......L
AtPAE5 S.MGMSDAKQAILTGCSAGGLASLIHCDYFRDHLPK.DAAVKCVSDGGYFLNV......P
OsPAE5 P.KGLARAKQAFLTGCSAGGLSTYIHCDDFRALLPK.DSTVKCLADGGFFLDV......E
SbPAE9 S.KGLARAKQAFLTGCSAGGLSTYIHCDDFRAVLPN.TPTVKCLADGGFFLDV......E
SbPAE12 V.KGLRNAKQAFLTGCSAGGLATYIHCDSFRALLPK.DSRVKCLADGGFFLDV......E
GRMZMPAE10.2 V.KGLRNAKQAFLTGCSAGGLATYIHCDSFRALLPK.DSRVKCLADGGFFLDV......E
BradiPAE5 L.KGLKHAKQAFLTGCSAGGLATYIHCDDFRALLPK.DSRVKCLADGGFFLDV......E
OsPAE8 L.KGLRHAKQAFLTGCSAGGLATFIHCDNFRTLLPK.DSRVKCLADGGFFLDV......E

```
                      200       210       220       230
                        .         .         .         .
SemoePAE7       DITGNYSLQSIFDITVREHNITLGIERNYVPTN....AAYK...................
PhpatPAE1       TYKGENKIQTYFKNVVDLHNVSGTLPEYCTETR....NSVE...................
SemoePAE6       DTSGNYALYSFYNGVVNTHSLKDTLPSSCISSK....DATQ...................
BradiPAE11      DITGNNYVRKSYKDIVNLHGSAKSLPSSCT.SK..R.SPEL...................
SbPAE11         DISGNYYARSIYKNVVNLHGSAKNLPASCT.SK....QPEL...................
GRMZMPAE1.3     DISGNFYARSIYKSVVNLHGSAKNLPASCT.SKPKQ.SPEL...................
GRMZMPAE1.6     DISGNFYARSIYKSVVNLHGSAKNLPASCT.SKPKQ.SPEL...................
MedtrPAE6       DISGAPHFEEYFNQIVTLHGSVKNLPGSCT.SK..L.KPEL...................
BradiPAE10      DIAGEKHAADFFNDVVTTHGSAKNLPSSCT.SK..L.PPGM...................
SbPAE10         DVAGVGYIAAFFNDVVTTHGSANNLPPSCT.SM..L.PPGM...................
OsPAE6          DIAGVEYIVAFFNGVATTHGSAKNLPSACT.SR..L.SPGM...................
MedtrPAE12      DISGDHYIEDYYSQVVATHGSEKSLPSSCT.SM..L.SPGL...................
LasatPAE1       TIIGQSHIEGFYADVVRTHGSAKVLSPACL.AK..M.SPGL...................
AtPAE8          DVSGVQYIKTYFEDVVTLHGSAKNLPRSCT.SR..L.TPAM...................
TheccPAE4       DVSGGHYIEAFFNQLVATHGSAKNLLPSCT.SR..M.RPGL...................
PotriPAE4       DVSGAPHVQTYFNEIVTLHGSAKNLPLSCT.SV..L.KPSF...................
RicomPAE1       DVSGAAHIQTYFNEVVSLHGSAKNLPLSCT.SR..L.KPAL...................
LichiPAE1       DVSGAQHIEAFYNEVVATHGSAKNLPASCT.SR..L.KPGL...................
MedtrPAE11      AVSGASHIEQFYSQVVQTHGSAKNLPSSCT.SR..L.SPGL...................
VradiPAE1       DVSGAQRIEEFYSQVVQTHGSAKNLPASCT.SR..L.RPGL...................
AtPAE7          DITGGSYIQSYYSKVVALHGSAKSLPVSCT.SK..M.KPEL...................
AtPAE11         DITGGSYIQSYYAKVVATHGSAKSLPASCT.SS..M.KPDL...................
TheccPAE5       DVSGGQHIENFYSQVAKLHGSVKSLPASCT.SRMSM.KPEL...................
PotriPAE3       DISGGSRIESFFGQVVKTHGSAKNLPASCT.SK..M.NPEL...................
PotriPAE2       DISGGSRIESFFGQVVKTHGSAKHLPASCT.SK..T.RPEL...................
BradiPAE9       ...................GAQKNLNKECLNSM..L.YPYQ...................
GRMZMPAE12.2    DISGSNTIRQFFSSLVSLQGIQKNLNMDCL.SSTSTDNAYL...................
LusPAE2         DVASNYTMRAFFEDLVTLQ.........................................
AtPAE9          DVAANRTMRSFYSQLVSLQGIQKNLDPSCTHAF..FPEPSL...................
PotriPAE8       DITLNYTMRTFFENLVSLQGIEKNLDKNCT.SF..LDNPKL...................
TheccPAE3       DVSLNHTVRSIFEDIVSLQGVEQNLDPKCT.KS..MNYPKL...................
RicomPAE6       DVTLNHTIRLFYENLVTLQGVEKNLNKNCT.SF..FNNPKL...................
TheccPAE2.2     DISLNYTMRSFYHDLVALQGIEENLNPNCT.MS..INNQKL...................
TheccPAE2.1     DISLNYTMRSFYHDLVALQGIEENLNPNCT.MS..INNQKL...................
GRMZMPAE3.1     DVSGGRSLRSYFGDIVAMQGIAQNLPPACT.AR..L.DATS...................
BradiPAE3       DVSGGRSLRSYYGEIVAMQEVARNLPPSCT.GH..L.DATS...................
SbPAE4.1        DVSGGRSLRSYYSDIVAMQGVAPNLPPACT.AR..L.DTTS...................
SbPAE4.2        DVSGGRSLRSYYSDIVAMQGVAPNLPPACT.AR..L.DTTS...................
BradiPAE1       DVAGRREMREFFNGIVRLQGSGRSLPRSCT.SR..M.DKTS...................
OsPAE9          DVSGQREMRDFFNGIVRLQGSGRSLPRSCT.SR..M.DKTS...................
GRMZMPAE8.1     DVSGRREMRSFFNGIVRLQGSGRSLPRSCT.SH..M.DKTS...................
GRMZMPAE8.2     DVSGRREMRSFFNGIVRLQGSGRSLPRSCT.SH..M.DKTS...................
SbPAE2          DVSGRREMRSFFNGIVRLQGSGRSLPRSCT.AR..M.DKTS...................
AtPAE1          DVSGGHSLRRMYSGVVNTQGLQNTLPPTCT.SH..I.KPFL...................
AtPAE2          DVSGGRSLRRLYAGVVRLQNLQTNLPQYCV.NR..L.NPTS...................
MedtrPAE5       DVSGRRSLRNLFGSVVTLQGAHKSLPRSCT.NH..L.NPIL...................
AtPAE3          DVSGGHSLRKMFQGVVTVQNLQKELSTACT.KH..L.DPTS...................
AtPAE6          DVSGGHSLRNMFQGVVTVQNLQKDLSSTCT.NH..L.DPTS...................
AlPAE8          DVSGGHSLRNMFQGVVTVQNLQKDLSSTCT.NH..L.DPTS...................
RicomPAE5       DVSGGRTLRNMYSGVVGLQGVQNNLPRICT.NH..L.DPTS...................
AtPAE12         DVSGGRTIRNLYNGVVELQSVKNNLPRICT.NH..L.DPTS...................
AtPAE10         DVSGGHTIRNLYNGVVQLQGVKNNLPHLCT.NH..L.NPTS...................
MedtrPAE10      DISGERTLRNMYNGVVGMQEAQKNLPQICT.NH..L.DPTS...................
MedtrPAE1       DVAGGHTLRNFFNGVVTLQGAQKNLPRVCT.NH..L.DPTS...................
MedtrPAE2       DISGGRTLRNMFGGVVQLQEVQKNLPKSCL.NK..M.DPTS...................
MedtrPAE9.1     DVFGGHTLRNLFGGVVNLQEVQKNLPKSCL.NH..L.DPTS...................
MedtrPAE9.2     DVFGGHTLRNLFGGVVNLQEVQKNLPKSCL.NH..L.DPTS...................
TheccPAE1       NVAGGHSLRDMYGGVVTLQGVQKNLPNTCT.SQ..M.DPTS...................
RicomPAE4       DVSGNRTLRNMYEGVVSLQKVQKNLPSTCT.SR..L.DPTS...................
BradiPAE2       DISGERFIRSVFSGVVHLQNVRKVLPKDCL.AK..K.EPTD...................
GRMZMPAE4.1     DLSGERHMRSVFSGVVQLQNVSGVLPKRCL.AK..K.DPAEARSCSLDLYDIFASPFNML
OsPAE3          DLSGKRLMWSVFNGTVQLQNVSEVLPKDCL.AK..K.VRTE...................
OsPAE2          DLSGERHMWSVFNGTVHLQNVREVLSKDCL.TK..K.DPTE...................
OsPAE4          DLSGERHMWSVFNGTVHLQNVTQVLSKDCL.AK..K.DPTE...................
AtPAE4          DVLGNPTMGSFFHDVVTLQSVDKSLDQNCV.AK..M.EPSK...................
AtPAE5          DVLGNPTMRSFYHDVVNLQGVEKSLDQKCV.AK..T.EPSK...................
OsPAE5          DISGRRYMRGFYNDVARQQDLRKRFPG.CS.SD..M.EPGQ...................
SbPAE9          DISGRRYMRGFYNDVARLQDVHKRFPH.CS.SD..M.EPGQ...................
SbPAE12         DISGRRTMQSFYSDVVRLQGLRERFSH.CN.SN..M.EAGQ...................
GRMZMPAE10.2    DISGRRTMHSFYSDIVRLQGLRERFSH.CN.SN..M.DAGQ...................
BradiPAE5       DISKQRTLRAFYSEVVRLQDLKRRFLH.CS.SS..E.DPGQ...................
OsPAE8          DISGQRTMRAFYNDVVRLQDLRGRFPH.CG.PN..M.DLGQ...................
```

```
                                   240       250       260       270
SemoePAE7      ............QLFPPYILPSIKQPMFLLNSAYDTWQIRNTLLYPTAE....WRPCVLN
PhpatPAE1      ............CFFPQYLISEMKTPLFVVNGAYDWWQMDNIVAP...DPLGEWDDCKNN
SemoePAE6      ............CFFPQNMQNYVGPPLFFVNGAYDFWQLENV....KRLSRDQYSSC.VD
BradiPAE11     ............CFFPQYVIPTLRTPLFILNAAYDTWQIRNILAPNAADPKKTWAKCKLD
SbPAE11        ............CMFPQYVVPTMRTPLFILNAAYDSWQVKNVLAPSPADPKKTWAQCKLD
GRMZMPAE1.3    ............CMFPQYVVPTMRTPLFILNAAYDSWQVKNVLAPSPADPKKTWAQCKLD
GRMZMPAE1.6    ............CMFPQYVVPTMRTPLFILNAAYDSWQVKNVLAPSPADPKKTWAQCKLD
MedtrPAE6      ............CFFPQNFVSQITTPIFVVNSPIDSYQIKNILAPDADD....WKNCKLD
BradiPAE10     ............CLFPQNEVKQIQTPLFILNAAYDSWQVRNILVPGGSDPH..WRSCKHD
SbPAE10        ............CFFPKNEVKQIHTPLFILNAAYDSWQVRNILVPGVADPHGKWHSCKHD
OsPAE6         ............CFFPQNEVKQIQTPLFILNAAYDSWQVRNILVPGFADPHGKWHSCKHD
MedtrPAE12     ............CFFPQYMASSIQTPIFIVNAAYDSWQIKNILAPGDADPDGQWRSCKTN
LasatPAE1      ............CFFPQNMVQFIKTPIFLINAAYDSWQVKNILAPGVADRKGTWRECKLD
AtPAE8         ............CFFPQYVARQIRTPLFILNAAYDSWQIKNILAPRAADPYGKWQSCQLD
TheccPAE4      ............CFFPEYMAQQIQTPLFFINAAYDSWQIRNILAPGIADPRGYWESCKLD
PotriPAE4      ............CFFPQYVAPQVRTPLFILNAAYDSWQIRNILAPSIADPLGVWKSCKLD
RicomPAE1      ............CFFPQYLVQQIRTPLFILNAAYDSWQIKNILAPGVADPRGSWNSCKLD
LichiPAE1      ............CFFPQYMARQIQTPLFIINAAYDSWQIKNILAPGVADPHGTWHSCKLD
MedtrPAE11     ............CFFPQNVAAQIKTPIFFVNAAYDSWQIKNILAPGVADPHGTWRNCKLD
VradiPAE1      ............CFFPQNVVSQISTPIFFVNAAYDSWQIKNILAPGAADPHGTWRECKLD
AtPAE7         ............CFFPQYVVPSMRTPLFVINAAFDSWQIKNVLAPTAVDKGKEWKNCKLD
AtPAE11        ............CFFPQYVAKTLQTPLFVINAAFDSWQIKNVLAPTSVDKSKAWKTCKLD
TheccPAE5      ............CFFPQYVVQTMQTPIFFINAAYDSWQIKNILAPTAADSGKAWKSCKLD
PotriPAE3      ............CFFPQYVAQTMRTPLFIINSAYDSWQIKNILAPTAVDSKKEWKDCKLD
PotriPAE2      ............CFFPQYVAQAMRTPLFIINSAYDSWQIKNILAPTAVDSKKEWKNCKLD
BradiPAE9      ............CFFPQYALQNIRTPYFILNSAYDVYQFHHTFVPPSCDPRGQWSRCKSD
GRMZMPAE12.2   ............CFFPQFALANIRTPFFILNSAYDVYQFHHILVPPSSDPGGHWSRCKSD
LusPAE2        ............CFFPQYALRYITTPFFLLNSAYDVYQINHILVPPSADVHGSWRGCKTR
AtPAE9         ............CFFPQYVLRFIKTPFFILNSAYDVFQFHHGLVPPSADQTGRWNRCKLN
PotriPAE8      ............CMFPQYFLNYMTTPFFILNTAYDVYQFHHALVPPSADMNGDWKRCKLS
TheccPAE3      ............CFFPQYALKYITTPFFVLNSAYDVVQFHRFLVPPSADVEGKWKHCKHD
RicomPAE6      ............CIFPQYALRFITTPFFILNSAYDVYQVNHILVPPSADLPGLWKNCKLN
TheccPAE2.2    ............CFFPQYALKYITTPYFILNSAYDVFQFHHSLVPPSADLHGRWNRCKLN
TheccPAE2.1    ............CFFPQYALKYITTPYFILNSAYDVFQFHHSLVPPSADLHGRWNRCKLN
GRMZMPAE3.1    ............CFFPQNIIDGVKTPLFLLNAAYDFIQIVLSLAPDRADPSGAWRACKSN
BradiPAE3      ............CFFPQNVIDSIKTPIFLLNAAYDAWQIEESLAPNRADPSGAWRACKYN
SbPAE4.1       ............CFFPQNVIDGINTPIFLLNAAYDVWQIQESLAPTGADPSGAWRACKSN
SbPAE4.2       ............CFFPQNVIDGINTPIFLLNAAYDVWQIQESLAPTGADPSGAWRACKSN
BradiPAE1      ............CFFPQNVLPNIQTPTFILNTAYDVWQLQQSVAPKTADPQGLWQRCKQN
OsPAE9         ............CFFPQNVVPNIQTPTFILNTAYDVWQLQQSVAPKRADPQGLWRGCRMN
GRMZMPAE8.1    ............CFFPQNVLPTIRTPTFVLNTAYDVWQLQQSVAPRTADPQGLWSKCRTN
GRMZMPAE8.2    ............CFFPQNVLPTIRTPTFVLNTAYDVWQLQQSVAPRTADPQGLWSKCRTN
SbPAE2         ............CFFPQNVLPNIQTPTFVLNTAYDVWQLQQSVAPRTADPQGLWSKCRTN
AtPAE1         ............CFFPQYIINQVKTPLFILNSGFDSWQIGNSLAPPSADKSGSWHNCSFS
AtPAE2         ............CFFPQNLINQVKTPLFILNAAYDSWQIQESLAPKSADPSGSWNDCRLN
MedtrPAE5      ............CFFPQHLIASVRTPLFLLNAAYDTWQIQASLAPPSADYHWNWYDCRKN
AtPAE3         ............CFFPQNLVSGIKTPMFLLNAAYDAWQVQESLAPPSVDLSGSWKACKSD
AtPAE6         ............CFFPQNLVSDIKTPMFLLNTAYDSWQIQESLAPPTADPGGIWKACKSD
AlPAE8         ............CFFPQNLVSDIKTPMFLLNTAYDSWQIQESLAPPTADPGGIWKACKSD
RicomPAE5      ............CFFPQNIIGNVKTPLFILNAAYDSWQIQSSLAPPSADPHGYWNECRKN
AtPAE12        ............CFFPQNLISQMKTPLFIVNAAYDTWQIQSSIAPTSADPSGFWHDCRLN
AtPAE10        ............CFFPQNLISQMKTPLFIVNAAYDIWQIQSSIAPPSADPSGYWHECRLN
MedtrPAE10     ............CFFPQNLIASVRTPLFLLNTAYDSWQIQSSLAPPSADPHGYWHECRLN
MedtrPAE1      ............CFFPENLIASVRTPLFILNTAYDSWQIQSSLAPSSADPHGNWRECRLN
MedtrPAE2      ............CFFPQNVVEHVETPLFLLNAAYDVWQIRASLAPSTADPLGSWNDCKSN
MedtrPAE9.1    ............CFFPQNLIDHVQTPLFLLNAAYDAWQFQESLAPHSADPHGSWNNCKSN
MedtrPAE9.2    ............CFFPQNLIDHVQTPLFLLNAAYDAWQFQESLAPHSADPHGSWNNCKSN
TheccPAE1      ............CFFPQNLVANIRTPLFLLNAAYDAWQVQESLIPSSADPHGSWRECKMD
RicomPAE4      ............CFFPQNLIANIKTPLFILNAAYDTWQVQASLAPPTADPQGSWNECKQN
BradiPAE2      ............CFFPAEVIKSINTPTFILNSGYDSWQIQNVLVPDETSPEKSWLTCKAN
GRMZMPAE4.1    SCQRFVFLDLRQCFFPAELIKSISTPTFIVNSEYDSWQIANVVAPDGSYTGDAWTSCRDN
OsPAE3         ............CFLATELVKSITAPTLIVNSAYDSWQIRDTLAPVGSYPGQSWLNCTND
OsPAE2         ............CFFPAELVKSITAPTLILNSAYDSWQIRNALAPDGSFPGQSWSSCKTD
OsPAE4         ............CFFPAELVKSITAPTLILNSAYDSWQIQNALAPDGSFPGQSWSSCKTD
AtPAE4         ............CMFPQESLKNIRTPVFLVNTAYDYWQIQNGLVPDSPDLDERWKICRLN
AtPAE5         ............CMFPQEFLKNIRTPVFLVNPAYDFWQIQHVLVPTSADPDKSWAKCRLN
OsPAE5         ............CFFPQEVAKGITTPMFILNPAYDVWQVEHVLTPDGSDPQNLWQDCRMD
SbPAE9         ............CFFPQEVAKSITTPMFILNPAYDVWQVEHVLSPEGSDPQNLWQNCRTD
SbPAE12        ............CLFPREVVKHIVNPVFVLNPAYDAWQVQHALAPEASDPQHSWLDCRLD
GRMZMPAE10.2   ............CFFPREVVKHIVNPVFVLNPAYDAWQVQHALAPEASDPQHSWLDCRLD
BradiPAE5      ............CFFPREVVKAIHTPVFVLNPAYDAWQVQHVLAPEASDPKHSWLDCRLD
OsPAE8         ............CFFPSEVVKDIITPVFVLNPAYDAWQVQHVLSPVASDPQHSWLECRLD
```

```
                 280       290       300           310       320
SemoePAE7        SSSCHPRQLQILQGFRSSFLTNISPAF...EKEKWGFFINSCFHHCQGDV.........
PhpatPAE1        AISCTNAQLEIIQGYRKELLEAL.KPI..QNSKKHGMFVDGCFHHCQASYDAFWSGP...
SemoePAE6        HSAC..PNVNVLQGFRQSMLDAL.SIS..RSRGSSGMFIDSCFSHCQVQGDDKW......
BradiPAE11       IKSCSSSQLVTLQNFRKDFLAAL.PQP..GQSPSLGIFIDSCFAHCQSGAQDTWIGE...
SbPAE11          IKSCSPSQLTTLQNFRTDFLAAL......PKTPSVGMFIDSCNAHCQSGSQDTWLAD...
GRMZMPAE1.3      IKSCSASQLTTLQNFRTDFLAAL......PKTQSVGMFIDSCNAHCQSGSQDTWLAD...
GRMZMPAE1.6      IKSCSASQLTTLQNFRTDFLAAL......PKTQSVGMFIDSCNAHCQSGSQDTWLAD...
MedtrPAE6        ITKCTPAQLNQVQGFRKEFLRAL.API..GNSSSVGTFIDSCYLHCQTELQEFWLFN...
BradiPAE10       INQCSEKQLKTLQGFRDDFLKAL.EEQ..GSSSSRGLFINSCFAHCQSEIQEIWFAP...
SbPAE10          IGQCSASQLRVLQGFRGDFLKEV.SEQ..ANSDSRGLFINSCFVHCQSESQELWFSS...
OsPAE6           IDQCPASQLQILQGFRDDFLKAL.KEQ..GTPSTRGLFINSCFVHCQSETQETWFAS...
MedtrPAE12       LNNCSPEQLNIMQDYRTQFLEAL.SPI..SNSPSNGMFIDSCYVHCQTEPQETWFKS...
LasatPAE1        ITKCSSAQLNVLQGYRLEFLKAL.NGFGNGNSPSRGMFINSCYSHCQTGIQETWLRN...
AtPAE8           IKNCHPSQIKVMQDFRLEFLSAV.IGL..GRSSSRGMFIDSCYTHCQTETQTSWFWQ...
TheccPAE4        IKNCLPSQIKTMQDFRLQFLVAL.LRL..GKSSSRGMFIDSCYAHCQTEMQGLWLMP...
PotriPAE4        INNCSPLQLKSMQDFRLQFLNAL.NKS..TNSSSRGLYIDSCYAHCQTETQEKWFME...
RicomPAE1        INNCSPIQLKTMQDFRMRFLSAL.YRS..ANSSSKGLYIDSCYAHCQTETQETWFMA...
LichiPAE1        INNCSPNQLQTMQSFRLQFLDAL.SGL..GNSTSKGLFIDSCYAHCQTEMQETWLRD...
MedtrPAE11       IKSCSANQLSTMQGFRTEFLKAI.SVV..SNSPSKGMFIDGCYSHCQTGMQETWMRT...
VradiPAE1        IKKCSSNQLSAMQVFRTDFLRAF.GAV..GNSPSKGHFIDGCYAHCQTGTQETWLRN...
AtPAE7           LKKCSAAQLKTVQGFRDQMMRAL.SPV..HSTPSRGLFLDSCHAHCQGGSAASWSGD...
AtPAE11          LKKCTAAQLQTVQGYRDQVLAAL.APV..RSATTNGLFLDSCHAHCQGGSAATWSGD...
TheccPAE5        LKKCTPGQLKIIQDFRAQFLSAL.TGA..GNSSSRGMFIDSCYAHCQAGKQITWSSN...
PotriPAE3        LKKCSASQLQTVQDYRTQFLKAVDTGL..GSSSTRGLWIDSCYAHCQSGSVSTWLND...
PotriPAE2        LKKCSATQLQTVQNYRTQFLKAVNIGL..GSSSSRGLWINSCYAHCQSGSVSTWLAD...
BradiPAE9        PAACSTSQIATLQGLRNAMLTAL.NLF..EGDSKVGMFINSCFAHCQSELQDTWFAP...
GRMZMPAE12.2     PGGCNATQIATLQGLRSGMLTSL.RQF..KSKPEAGMFINSCFAHCQSELQDTWFAP...
LusPAE2          ISGCTPTQIEDLQGLRIEMLKASLAFY..RGVDMNGMFINSCFSHCQSEFQPTWFDL...
AtPAE9           VTACNPHQLDALQGFRKDMLGALMNFF..RNSTRGGMFINSCFDHCQSALEETWLSP...
PotriPAE8        IASCTPQQLDILQGW.GDMLAALSSFL..KDSTSGGMFINSCFAHCQSETQETWFAL...
TheccPAE3        PAACSADQIAILQGFRRDMLEALNFFL..KDSHRGGTFINSCFAHCQSDAQESWFAV...
RicomPAE6        TADCSETQIGVLQGFRRDMLVALRIFY..KYSNSVGMFINSCFAHCQSESQDTWFAV...
TheccPAE2.2      PAACNASQINVLQGLRQDMLTALYSFY..KNSSRGGMFINSCFAHCQSESQDTWFAV...
TheccPAE2.1      PAACNASQINVLQGLRQDMLTALYSFY..KNSSRGGMFINSCFAHCQSESQDTWFAV...
GRMZMPAE3.1      RTACSASQMSFLQDFRDQMVASV.KGF..SGSRSNGVFLSSCFAHCQSEQLGTWNTKP..
BradiPAE3        RSACDASQIKFLQSFRDQMVASV.KAF..SGSRSNGLFINSCFAHCQSELPATWNDAP..
SbPAE4.1         HSACDASQMKFLQDFRDQMVASVNNGF..AGSRSNGLFINSCFAHCQSELPMTWSDNAAG
SbPAE4.2         HSACDASQMKFLQDFRDQMVASVNNGF..AGSRSNGLFINSCFAHCQSELPMTWSDNAAG
BradiPAE1        HAFCSGNQLQFLNGFRNEMLDAV.KGF..SGSRQNGVFINSCFAHCQSERQDTWYSN...
OsPAE9           HASCNSNQLQFLQGFRNQMLDAV.RGF..SGARQNGLFINSCFAHCQSERQDTWYAG...
GRMZMPAE8.1      HAFCNSNQLQFLQGFRNQMLDAV.RGF..SASRQNGLFINSCFAHCQSERQDTWYAN...
GRMZMPAE8.2      HAFCNSNQLQFLQGFRNQMLDAV.RGF..SASRQNGLFINSCFAHCQSERQDTWYAN...
SbPAE2           HAFCNSNQLQFLQGFRNQMLDAV.KGF..SASRRNGLFINSCFAHCQSERQDTWYAN...
AtPAE1           F.RCTASQMHFLEGFKMSMLDAL.KTF..SKFSKNGVLITSGWAHCQAERQDTWFPG...
AtPAE2           YAKCSASQIQFLQGFRTRMVNLV.KGF..AMPSKNGVFLNSCFAHCQTERHDTWFAQ...
MedtrPAE5        YARCSSPQIQYLQGFRNQMLRVT.RRF..SRSRQNGLFINSCFAHCQSERQDTWHAR...
AtPAE3           HSHCNSSQIQFFQDFRTHMVDAV.KSF..ATSTHNGVFINSCFAHCQSERQDTWYAP...
AtPAE6           HSRCNSSQIQFFQEFRNQMLFAV.NSF..SNSDQNGLYINSCFAHCQTERQDTWFAQ...
AlPAE8           HSRCNSSQIQFFEEFRTQMVLAV.NSF..SNSDQNGLYINSCFAHCQTERQDTWFAQ...
RicomPAE5        HAKCSAPQIQFLQGFRNQMLRAI.RGF..SMSKQNGLFINSCFAHCQSERQDTWFAD...
AtPAE12          HGKCTPAQLRFLQGFREQMLRVV.KGF..SMSRQNGLFINSCFAHCQTERQDTWFAD...
AtPAE10          HGRCTPAQIRFLQGFRNQMLRAV.SGF..SNSKKNGLFINSCFAHCQTERQDTWFAD...
MedtrPAE10       HAKCTRPQIKFLQGFRTHMLNSI.KDF..SRSNKNGLFINSCFAHCQTERQDTWFSD...
MedtrPAE1        HNKCSGSQIQFLQGFRNHMVNVV.RGF..SRSSQNGLFINSCFAHCQSERQDTWFAD...
MedtrPAE2        NANCNSSQIQLLQDFRNQMLDDV.KDF..SRSSQTGLFINSCFAHCQSERQETWFAD...
MedtrPAE9.1      HANCNSSQIQILQNFRNQMLNDI.KGF..STTSQSGLFINSCFAHCQSERQDTWFAD...
MedtrPAE9.2      HANCNSSQIQILQNFRNQMLNDI.KGF..STTSQSGLFINSCFAHCQSERQDTWFAD...
TheccPAE1        RSHCNSTQMQFLQDFRSQMLNAI.NVF..SKSNQNGLFINSCFAHCQSERQDTWYEN...
RicomPAE4        HAQCNSSQIQFLQDFRNQMLDAI.NVF..SMTTQNGLFINSCFSHCQSERQDTWFAT...
BradiPAE2        IRECNPTQIEALHGFRETLVNDL.KVV..QDKEDWGLFIDSCFTHCQTPFRISWDSP...
GRMZMPAE4.1      IRNCSSEQMDVLHGFRAELIREL.KVA..EGEREWGLFVDSCFTHCQTQSSDWWHSP...
OsPAE3           IGNCNSTQMEVLNGFRKKFVDGV.KVV..KDKKDWGLFIDSCFMHCQTKYSISWSSQ...
OsPAE2           IRNCSSTQIQVFNGFRNKFVDDV.EIV..KDKKDWGLFIDSCFTHCQTPFNISWSSQ...
OsPAE4           IRNCSSTQIQVFNGFRNKFVDDI.EVV..KDKKDWGLFIDSCFTHCQTPFDISWNSQ...
AtPAE4           IQECDAAQMKVLHGFRSSLIDAI.GEF..HVNKEGGMFINSCNSHCQ..IRESWHSA...
AtPAE5           IKECDAEQIKVLHGFRSSMMTAI.GEF..HQNKDGGMFIDSCYAHCQTVMSVTWHSL...
OsPAE5           ITKCNTKQLEILQGFRKSLLDAI.SEF..KKKRGWGMFIDSCFIHCQSMKSLAWHSP...
SbPAE9           ITKCSSKQLEVLQGFRKALLDAI.NEF..KKRRDWGMFIDSCFIHCQSMKALAWHSP...
SbPAE12          ISKCGSEQLEILQGFRKELHDAI.SEV..KQKRDWGFYINSCFVHCQSLNSLTWHSP...
GRMZMPAE10.2     ISKCSPKQLGILQGFRKELHDAI.SEA..KQKRGWGFYINSCFVHCQSLNSLTWHSP...
BradiPAE5        ISKCNPNQLKILQGFREELHVAM.SEL..KQKKDWGIFIDSCFVHCQSLNSLTWHSP...
OsPAE8           ISKCDSNQLEILQGFRKKLHDTI.SEL..KHKKDWGFFIDSCFIHCQSLNSLTWHSP...
```

```
                          330          340
SemoePAE7     ..STVRVNNQTILEAIGNWMYER.....................................
PhpatPAE1     ..HAPHVKGKTASQALGDWYFER.....................................
SemoePAE6     ..NNPKVNGLSTAKTVGDWYFGR.....................................
BradiPAE11    ..GSPSIQKMRIGKAVGDWFYNR.....................................
SbPAE11       ..GSPTVNKTQIGKAVGDWYFDR.....................................
GRMZMPAE1.3   ..GSPTVNKTQIGKAVGDWYYDR.....................................
GRMZMPAE1.6   ..GSPTVNKTQIGKAVGDWYYDR.....................................
MedtrPAE6     ..GSPLLANTTIAKSVADWFYDR.....................................
BradiPAE10    ..DSPVLGNKKIANAIGDWFYDR.....................................
SbPAE10       ..DSPKLGNTTIANAVGDWFFGR.....................................
OsPAE6        ..GSPMLETKTIADAVGDWFYDR.....................................
MedtrPAE12    ..DSPMVGNKTVAKAVGDWFYER.....................................
LasatPAE1     ..DSPLLGNTTIAKAVGDWYYER.....................................
AtPAE8        ..DSPILNRTTIAKAVGDWVYDR.....................................
TheccPAE4     ..DSPLLNKTSIAKAVGDWFYDR.....................................
PotriPAE4     ..DSPVLGKKKIAKAVGDWFYDR.....................................
RicomPAE1     ..DSPVLSKTKIAKAVGDWFYDR.....................................
LichiPAE1     ..DSPVLGKTTIAKAVGDWYYDR.....................................
MedtrPAE11    ..DSPVLAKTTIAKAVGDWYYDR.....................................
VradiPAE1     ..DSPVLGSTTIAKAVGDWYYDR.....................................
AtPAE7        ..KGPQVANTRIAKAVGNWFYGR.....................................
AtPAE11       ..KGPTVANTKMAKAVGDWFFER.....................................
TheccPAE5     ..NSPAVGNTKIAKAVGDWYYER.....................................
PotriPAE3     ..KSPEVGDTKMGKAVGDWFYDR.....................................
PotriPAE2     ..KSPVVGNVKIGKAVGDWFYDR.....................................
BradiPAE9     ..NSPSLHNKTIAELVGDWYFER.....................................
GRMZMPAE12.2  ..NSPSIDNKKIAEVVGDWYFER.....................................
LusPAE2       ..NSPMIQNKTIAEAVGDWYFGRKVAMFPPFTMRHITPSVHLANQLAARGHRVSLLLLTR
AtPAE9        ..TSPRINNKTIAETVGDWYFGR.....................................
PotriPAE8     ..GSPSIQDKTIAGAVGDWYFSR.....................................
TheccPAE3     ..NSPRIHNKTIAEVVGDWYFTR.....................................
RicomPAE6     ..DSPRIHNKTIAETVGDWYFSR.....................................
TheccPAE2.2   ..DSPRIHNKRLLQ..............................................
TheccPAE2.1   ..DSPRIHNKTIAEAVGDWYFSR.....................................
GRMZMPAE3.1   .GGSPTIQNKGISKSVGDWYFDR.....................................
BradiPAE3     ..GSPAVQNKGIAKSVGDWYFGR.....................................
SbPAE4.1      GGASPAIQSRGIAKSVGDWYFGR.....................................
SbPAE4.2      GGASPAIQSRVTCNAAIHIY........................................
BradiPAE1     ..NSPRLGNRRIAEAVGDWFFER.....................................
OsPAE9        ..DSPRLGNKRIAEAVGDWFFDR.....................................
GRMZMPAE8.1   ..NSPRLGNKKIADAVGDWFFER.....................................
GRMZMPAE8.2   ..NSPRLGNKKIADAVGDWFFER.....................................
SbPAE2        ..NSPRLGNKKIADAVGDWFFER.....................................
AtPAE1        ..YSGAGKAKGIAVAVGDWYFER.....................................
AtPAE2        ..NSPAIKNKGIAVAVGDWYFER.....................................
MedtrPAE5     ..GSPHIGNKGIADSVGNWFFDR.....................................
AtPAE3        ..DSPTLHGKTVAESVGDWYFDR.....................................
AtPAE6        ..DSPQLNGKRVAESVGDWYFDR.....................................
AlPAE8        ..DSPQLNGKRVAESVGDWYFDR.....................................
RicomPAE5     ..DSPVIGNKAVAIAVGDWYFDR.....................................
AtPAE12       ..DSPVIRKKAVAIAVGDWYFDR.....................................
AtPAE10       ..DSPVIHKKAVAIAVGDWYFDR.....................................
MedtrPAE10    ..NSPVIRNKVIALAVGDWYFDR.....................................
MedtrPAE1     ..NSPVIGNKAIALAVGDWYFDR.....................................
MedtrPAE2     ..DSPLIDDKPIAVAVGDWYFDR.....................................
MedtrPAE9.1   ..DSPLLNNMPIAVAIGNWFFDR.....................................
MedtrPAE9.2   ..DSPLLNNMPIAVAIGNWFFDR.....................................
TheccPAE1     ..DSPRIGNKAVAVSVGDWFFDR.....................................
RicomPAE4     ..DSPVIRDKRISQSVGDWYFDR.....................................
BradiPAE2     ..ISPRLQNKSIAEAVGDWHFGR.....................................
GRMZMPAE4.1   ..TSPRLGNQTVAEAVGDWYFGR.....................................
OsPAE3        ..FSPVLGNMTIAKAVGDWYFER.....................................
OsPAE2        ..ASPVLGSKTVAEAVGDWYFER.....................................
OsPAE4        ..ASPVLGNKIVAEAIGDWYFER.....................................
AtPAE4        ..TSTRIENKTIAESVGDWYFNR.....................................
AtPAE5        ..TSPRIENKTIAESVGDWYFNR.....................................
OsPAE5        ..SASRINNKTVAEAVGDWFFDR.....................................
SbPAE9        ..SAARINNKTAAEAVGDWFFDR.....................................
SbPAE12       ..TSPRVNNKSIAEAVGDWFFDR.....................................
GRMZMPAE10.2  ..TSPRVNNKSIAEAVGDWFFDR.....................................
BradiPAE5     ..SSPRVSNKTIAEAVGDWFFDR.....................................
OsPAE8        ..SSLRVNNKTIAEAVGDWFFDR.....................................
```

```
                                                      350         360
                                                        .           .
SemoePAE7      .............................QKK..VILVDFLSWP.NNPTCV.........
PhpatPAE1      .............................D.TTASSVID.CAYP.CNPTCP.........
SemoePAE6      .............................SSS..SIHID.CAYP.CNPTCV.........
BradiPAE11     .............................H.V..SQLID.CPYP.CNPTCK.........
SbPAE11        .............................E.V..SRQID.CPYP.CNPTCK.........
GRMZMPAE1.3    .............................E.V..PRQID.CPYP.CNPTCK.........
GRMZMPAE1.6    .............................E.V..PRQID.CPYP.CNPTCK.........
MedtrPAE6      .............................R.P..FHQID.CPYP.CNPTCH.........
BradiPAE10     .............................S.P..FQEID.CPYP.CDSSCH.........
SbPAE10        .............................S.S..FQKID.CPYP.CDSTCH.........
OsPAE6         .............................N.P..FQKID.CPYP.CDSTCH.........
MedtrPAE12     .............................S.P..SREID.CTYP.CNPTCQ.........
LasatPAE1      .............................N.T..FQKID.CAYP.CDKTCH.........
AtPAE8         .............................T.L..FQKID.CPYP.CNPTCH.........
TheccPAE4      .............................N.P..FQKID.CAYP.CNPTCH.........
PotriPAE4      .............................N.P..FQKID.CPYP.CNPSCQ.........
RicomPAE1      .............................I.P..FQKID.CPYP.CNPTCS.........
LichiPAE1      .............................S.P..FQKID.CPYP.CNPTCH.........
MedtrPAE11     .............................S.T..FQQID.CPYP.CNPTCH.........
VradiPAE1      .............................K.P..FKQID.CAYP.CNPTCH.........
AtPAE7         .............................S.A..FQKID.CPSPTCNPTCP.........
AtPAE11        .............................S.T..FQNVD.CSSLNCNPTCP.........
TheccPAE5      .............................S.P..VQKID.CPYP.CNPTCQ.........
PotriPAE3      .............................S.A..MEKID.CPYS.CNPTCV.........
PotriPAE2      .............................S.A..FEKID.CAYP.CNPTCV.........
BradiPAE9      .............................G.A..AQEID.CAYP.CDLTCH.........
GRMZMPAE12.2   .............................G.A..AVEID.CAYP.CDSTCR.........
LusPAE2        TILRVTNLNTHPNLVTFHPITVPHVEGLPQ.G..AELIG.AEVP.PHLTCHIFTAIDATE
AtPAE9         .............................GEE..AKEIG.CPYP.CDKTCH.........
PotriPAE8      .............................N.I..SKLID.CAYP.CDASCH.........
TheccPAE3      .............................R.E..AKEID.CEYP.CDTTCH.........
RicomPAE6      .............................N.R..SKEID.CPYP.CDDTCH.........
TheccPAE2.2    ...................................................K.........
TheccPAE2.1    .............................R.V..TKEID.CPYP.CDTTCH.........
GRMZMPAE3.1    .............................A.E..VKAVD.CRYP.CDNTCH.........
BradiPAE3      .............................A.E..VKAID.CPYP.CDNTCR.........
SbPAE4.1       .............................A.Q..VKAID.CPYP.CDRTCR.........
SbPAE4.2       .............................T.H..IKAIK.....................
BradiPAE1      .............................G.D..AKYTD.CTYP.CDGTCH.........
OsPAE9         .............................A.D..AKYTD.CAYP.CDGTCH.........
GRMZMPAE8.1    .............................G.N..AKYTD.CPYP.CDGTCH.........
GRMZMPAE8.2    .............................G.N..AKYTD.CPYP.CDGTCH.........
SbPAE2         .............................G.D..AKYTD.CPYP.CDGTCH.........
AtPAE1         .............................T...............KQNSS..........
AtPAE2         .............................G.G..AKLID.CAYP.CDKTCH.........
MedtrPAE5      .............................V.G..VQAIG.CPYP.CDKTCH.........
AtPAE3         .............................T.T..VKAID.CPYP.CDKTCH.........
AtPAE6         .............................AKN..VKAID.CPYP.CDTTCH.........
AlPAE8         .............................ANN..VKAID.CPYP.CDTTCH.........
RicomPAE5      .............................S.G..VKLID.CPYP.CDTPAT.........
AtPAE12        .............................A.E..VKLVD.CPYP.CDKSCH.........
AtPAE10        .............................A.E..VKLID.CPYP.CDRSCH.........
MedtrPAE10     .............................E.G..VKVID.CPYP.CDNTCH.........
MedtrPAE1      .............................A.A..VKDID.CPYP.CDNTCH.........
MedtrPAE2      .............................E.V..VKAID.CPYP.CDNSCH.........
MedtrPAE9.1    .............................Q.V..VKAID.CAYP.CDNTCH.........
MedtrPAE9.2    .............................Q.V..VKAID.CAYP.CDNTCH.........
TheccPAE1      .............................T.A..VKAID.CAYP.CDSTCH.........
RicomPAE4      .............................V.D..VKAID.CAYP.CDSSCH.........
BradiPAE2      .............................S.RSGVKQID.CEYP.CNPTCS.........
GRMZMPAE4.1    .............................R.RV.VKQVD.CEYP.CNPTCS.........
OsPAE3         .............................SKT..VKEID.CEYP.CNPTCK.........
OsPAE2         .............................SYE..VKEID.CEYP.CNPTCS.........
OsPAE4         .............................SYE..VKEID.CEYP.CNPTCS.........
AtPAE4         .............................K.P..VKLID.CPYP.CNASC..........
AtPAE5         .............................K.P..VKLID.CPYP.CNPSCY.........
OsPAE5         .............................R.E..VKEID.CEYP.CNPTCF.........
SbPAE9         .............................R.E..VKEID.CEYP.CNPTCY.........
SbPAE12        .............................R.E..VKEID.CEYP.CNPTCH.........
GRMZMPAE10.2   .............................R.E..VKEID.CEYP.CNPTCH.........
BradiPAE5      .............................R.E..VKELD.CEYP.CNPTCH.........
OsPAE8         .............................R.E..VKEID.CEYP.CNPTCH.........
```

```
                                                                370
SemoePAE7       ................................................TYFEDTWL....
PhpatPAE1       ...............................................PKTSGRLSVGRPS
SemoePAE6       ...............................................TRLD.........
BradiPAE11      ...............................................NREED........
SbPAE11         ...............................................NREDD........
GRMZMPAE1.3     ...............................................NRDDD........
GRMZMPAE1.6     ...............................................NRDDD........
MedtrPAE6       ...............................................THVNQDAEL....
BradiPAE10      .................................................VFKNSSET...
SbPAE10         ...............................................NGIYEDSSQA...
OsPAE6          ...............................................NRIYDDPSEA...
MedtrPAE12      ...............................................NRVWESATASYPA
LasatPAE1       ...............................................NRVFE........
AtPAE8          ...............................................HRVFTPLDAPPI.
TheccPAE4       ...............................................NRIYEQPAHHYPN
PotriPAE4       ...............................................NSGLAPPDNPEV.
RicomPAE1       ...............................................NNGLNPRDNPEV.
LichiPAE1       ...............................................NRVFDPDDHPGV.
MedtrPAE11      ...............................................NRVFE........
VradiPAE1       ...............................................NRIFDQNERPDV.
AtPAE7          ...............................................AISTED.......
AtPAE11         ...............................................AVSTED.......
TheccPAE5       ...............................................NTDSESQLPDI..
PotriPAE3       ...............................................SVDSES.......
PotriPAE2       ...............................................SIDSES.......
BradiPAE9       ...............................................NIIPTGYPFDRVK
GRMZMPAE12.2    ...............................................NLIPIDKNGFAGA
LusPAE2         EQVRNIIGHVKPDFFLFDSAPWIPRAGRMLGFVSVSYSPVSAIWTALRMVPSAKIVKGMS
AtPAE9          ...............................................NLIPASTSDFLAS
PotriPAE8       ...............................................NIIAQVS......
TheccPAE3       ...............................................NLMPVPQISLMIH
RicomPAE6       ...............................................NLIPTARDDRRFL
TheccPAE2.2     ...............................................QLVIGTLAEE...
TheccPAE2.1     ...............................................NLIPSPQGLR...
GRMZMPAE3.1     ...............................................HII..........
BradiPAE3       ...............................................HII..........
SbPAE4.1        ...............................................NII..........
SbPAE4.2        ............................................................
BradiPAE1       ...............................................HLVFRGRGL....
OsPAE9          ...............................................HLTFRGDY.....
GRMZMPAE8.1     ...............................................HLVFRGDH.....
GRMZMPAE8.2     ...............................................HLVFRGDH.....
SbPAE2          ...............................................HLVFRGDH.....
AtPAE1          ............................................................
AtPAE2          ...............................................NLVFRR.......
MedtrPAE5       ...............................................NLVFN........
AtPAE3          ...............................................NLIFK........
AtPAE6          ...............................................NLIFE........
AlPAE8          ...............................................NLIFE........
RicomPAE5       ...............................................IWVFR........
AtPAE12         ...............................................NLVFR........
AtPAE10         ...............................................NLVFR........
MedtrPAE10      ...............................................HLVFS........
MedtrPAE1       ...............................................HLVFR........
MedtrPAE2       ...............................................NLVFRRV......
MedtrPAE9.1     ...............................................NLVFK........
MedtrPAE9.2     ...............................................NLVFNVGKSAMVD
TheccPAE1       ...............................................DLVFNDVTSTITI
RicomPAE4       ...............................................NLVFK........
BradiPAE2       ...............................................TQLPS........
GRMZMPAE4.1     ...............................................IQ...........
OsPAE3          .................................................LTG........
OsPAE2          ...............................................SQLPT........
OsPAE4          ...............................................SQLPK........
AtPAE4          ............................................................
AtPAE5          ...............................................NMNFT........
OsPAE5          ...............................................NVVLEQPYQEG..
SbPAE9          ...............................................NVVLDQPYKED..
SbPAE12         ...............................................NLVFAKAFKI...
GRMZMPAE10.2    ...............................................NLVFARPFKI...
BradiPAE5       ...............................................NLVFDKPFKG...
OsPAE8          ...............................................NLVFAKPFKA...
```

```
SemoePAE7      ............................................................
PhpatPAE1      FRNWSKRQ....................................................
SemoePAE6      ............................................................
BradiPAE11     ............................................................
SbPAE11        ............................................................
GRMZMPAE1.3    ............................................................
GRMZMPAE1.6    ............................................................
MedtrPAE6      ............................................................
BradiPAE10     ............................................................
SbPAE10        ............................................................
OsPAE6         ............................................................
MedtrPAE12     MYTRVY......................................................
LasatPAE1      ............................................................
AtPAE8         ............................................................
TheccPAE4      L...........................................................
PotriPAE4      ............................................................
RicomPAE1      ............................................................
LichiPAE1      ............................................................
MedtrPAE11     ............................................................
VradiPAE1      ............................................................
AtPAE7         ............................................................
AtPAE11        ............................................................
TheccPAE5      ............................................................
PotriPAE3      ............................................................
PotriPAE2      ............................................................
BradiPAE9      EIPEF.......................................................
GRMZMPAE12.2   ............................................................
LusPAE2        DEELGRAPPGYPSSVVVPRRDEIAAVRVFAMEFGTVSLYERIVSVIREGEVMAMRSCREL
AtPAE9         NASGSGHNSRGTHLTFLFLLLNFFFFFVISKFSKKDYVT.....................
PotriPAE8      ............................................................
TheccPAE3      K...........................................................
RicomPAE6      SNASDNDLDSCDDCPKGHEEKNSGFIKPMHGTGMALWLLFLFLLV...............
TheccPAE2.2    ............................................................
TheccPAE2.1    ............................................................
GRMZMPAE3.1    ............................................................
BradiPAE3      ............................................................
SbPAE4.1       ............................................................
SbPAE4.2       ............................................................
BradiPAE1      ............................................................
OsPAE9         ............................................................
GRMZMPAE8.1    ............................................................
GRMZMPAE8.2    ............................................................
SbPAE2         ............................................................
AtPAE1         ............................................................
AtPAE2         ............................................................
MedtrPAE5      ............................................................
AtPAE3         ............................................................
AtPAE6         ............................................................
AlPAE8         ............................................................
RicomPAE5      ............................................................
AtPAE12        ............................................................
AtPAE10        ............................................................
MedtrPAE10     ............................................................
MedtrPAE1      ............................................................
MedtrPAE2      ............................................................
MedtrPAE9.1    ............................................................
MedtrPAE9.2    SQSTSDDIPTSMQYSRSTRLTFSAGLFVLSALLPITCS......................
TheccPAE1      SQSTRLTFTLLNLLSALLITLTCSKCTMEFQFGQ..........................
RicomPAE4      ............................................................
BradiPAE2      ............................................................
GRMZMPAE4.1    ............................................................
OsPAE3         ............................................................
OsPAE2         ............................................................
OsPAE4         ............................................................
AtPAE4         ............................................................
AtPAE5         ............................................................
OsPAE5         ............................................................
SbPAE9         ............................................................
SbPAE12        ............................................................
GRMZMPAE10.2   ............................................................
BradiPAE5      ............................................................
OsPAE8         ............................................................
```

**SemoePAE7**	....
**PhpatPAE1**	....
**SemoePAE6**	....
**BradiPAE11**	....
**SbPAE11**	....
**GRMZMPAE1.3**	....
**GRMZMPAE1.6**	....
**MedtrPAE6**	....
**BradiPAE10**	....
**SbPAE10**	....
**OsPAE6**	....
**MedtrPAE12**	....
**LasatPAE1**	....
**AtPAE8**	....
**TheccPAE4**	....
**PotriPAE4**	....
**RicomPAE1**	....
**LichiPAE1**	....
**MedtrPAE11**	....
**VradiPAE1**	....
**AtPAE7**	....
**AtPAE11**	....
**TheccPAE5**	....
**PotriPAE3**	....
**PotriPAE2**	....
**BradiPAE9**	....
**GRMZMPAE12.2**	....
**LusPAE2**	EGKYVDYLEEQYGKRVLLTGPSLPKPDGLGVDEELGSCLSKFEPNSVVYCAFGREFVLHK
**AtPAE9**	....
**PotriPAE8**	....
**TheccPAE3**	....
**RicomPAE6**	....
**TheccPAE2.2**	....
**TheccPAE2.1**	....
**GRMZMPAE3.1**	....
**BradiPAE3**	....
**SbPAE4.1**	....
**SbPAE4.2**	....
**BradiPAE1**	....
**OsPAE9**	....
**GRMZMPAE8.1**	....
**GRMZMPAE8.2**	....
**SbPAE2**	....
**AtPAE1**	....
**AtPAE2**	....
**MedtrPAE5**	....
**AtPAE3**	....
**AtPAE6**	....
**AlPAE8**	....
**RicomPAE5**	....
**AtPAE12**	....
**AtPAE10**	....
**MedtrPAE10**	....
**MedtrPAE1**	....
**MedtrPAE2**	....
**MedtrPAE9.1**	....
**MedtrPAE9.2**	....
**TheccPAE1**	....
**RicomPAE4**	....
**BradiPAE2**	....
**GRMZMPAE4.1**	....
**OsPAE3**	....
**OsPAE2**	....
**OsPAE4**	....
**AtPAE4**	....
**AtPAE5**	....
**OsPAE5**	....
**SbPAE9**	....
**SbPAE12**	....
**GRMZMPAE10.2**	....
**BradiPAE5**	....
**OsPAE8**	....

```
SemoePAE7       ............................................................
PhpatPAE1       ............................................................
SemoePAE6       ............................................................
BradiPAE11      ............................................................
SbPAE11         ............................................................
GRMZMPAE1.3     ............................................................
GRMZMPAE1.6     ............................................................
MedtrPAE6       ............................................................
BradiPAE10      ............................................................
SbPAE10         ............................................................
OsPAE6          ............................................................
MedtrPAE12      ............................................................
LasatPAE1       ............................................................
AtPAE8          ............................................................
TheccPAE4       ............................................................
PotriPAE4       ............................................................
RicomPAE1       ............................................................
LichiPAE1       ............................................................
MedtrPAE11      ............................................................
VradiPAE1       ............................................................
AtPAE7          ............................................................
AtPAE11         ............................................................
TheccPAE5       ............................................................
PotriPAE3       ............................................................
PotriPAE2       ............................................................
BradiPAE9       ............................................................
GRMZMPAE12.2    ............................................................
LusPAE2         DQFQELLSDCQILLVPNITDQIVSTMFMAKELKVALEVDKDENGWISKEKVCKAIGAVMD
AtPAE9          ............................................................
PotriPAE8       ............................................................
TheccPAE3       ............................................................
RicomPAE6       ............................................................
TheccPAE2.2     ............................................................
TheccPAE2.1     ............................................................
GRMZMPAE3.1     ............................................................
BradiPAE3       ............................................................
SbPAE4.1        ............................................................
SbPAE4.2        ............................................................
BradiPAE1       ............................................................
OsPAE9          ............................................................
GRMZMPAE8.1     ............................................................
GRMZMPAE8.2     ............................................................
SbPAE2          ............................................................
AtPAE1          ............................................................
AtPAE2          ............................................................
MedtrPAE5       ............................................................
AtPAE3          ............................................................
AtPAE6          ............................................................
AlPAE8          ............................................................
RicomPAE5       ............................................................
AtPAE12         ............................................................
AtPAE10         ............................................................
MedtrPAE10      ............................................................
MedtrPAE1       ............................................................
MedtrPAE2       ............................................................
MedtrPAE9.1     ............................................................
MedtrPAE9.2     ............................................................
TheccPAE1       ............................................................
RicomPAE4       ............................................................
BradiPAE2       ............................................................
GRMZMPAE4.1     ............................................................
OsPAE3          ............................................................
OsPAE2          ............................................................
OsPAE4          ............................................................
AtPAE4          ............................................................
AtPAE5          ............................................................
OsPAE5          ............................................................
SbPAE9          ............................................................
SbPAE12         ............................................................
GRMZMPAE10.2    ............................................................
BradiPAE5       ............................................................
OsPAE8          ............................................................
```

```
SemoePAE7       ...........................
PhpatPAE1       ...........................
SemoePAE6       ...........................
BradiPAE11      ...........................
SbPAE11         ...........................
GRMZMPAE1.3     ...........................
GRMZMPAE1.6     ...........................
MedtrPAE6       ...........................
BradiPAE10      ...........................
SbPAE10         ...........................
OsPAE6          ...........................
MedtrPAE12      ...........................
LasatPAE1       ...........................
AtPAE8          ...........................
TheccPAE4       ...........................
PotriPAE4       ...........................
RicomPAE1       ...........................
LichiPAE1       ...........................
MedtrPAE11      ...........................
VradiPAE1       ...........................
AtPAE7          ...........................
AtPAE11         ...........................
TheccPAE5       ...........................
PotriPAE3       ...........................
PotriPAE2       ...........................
BradiPAE9       ...........................
GRMZMPAE12.2    ...........................
LusPAE2         EESEVGKEVTMNHLKWGEVLGDDRFFG
AtPAE9          ...........................
PotriPAE8       ...........................
TheccPAE3       ...........................
RicomPAE6       ...........................
TheccPAE2.2     ...........................
TheccPAE2.1     ...........................
GRMZMPAE3.1     ...........................
BradiPAE3       ...........................
SbPAE4.1        ...........................
SbPAE4.2        ...........................
BradiPAE1       ...........................
OsPAE9          ...........................
GRMZMPAE8.1     ...........................
GRMZMPAE8.2     ...........................
SbPAE2          ...........................
AtPAE1          ...........................
AtPAE2          ...........................
MedtrPAE5       ...........................
AtPAE3          ...........................
AtPAE6          ...........................
AlPAE8          ...........................
RicomPAE5       ...........................
AtPAE12         ...........................
AtPAE10         ...........................
MedtrPAE10      ...........................
MedtrPAE1       ...........................
MedtrPAE2       ...........................
MedtrPAE9.1     ...........................
MedtrPAE9.2     ...........................
TheccPAE1       ...........................
RicomPAE4       ...........................
BradiPAE2       ...........................
GRMZMPAE4.1     ...........................
OsPAE3          ...........................
OsPAE2          ...........................
OsPAE4          ...........................
AtPAE4          ...........................
AtPAE5          ...........................
OsPAE5          ...........................
SbPAE9          ...........................
SbPAE12         ...........................
GRMZMPAE10.2    ...........................
BradiPAE5       ...........................
OsPAE8          ...........................
```
