# Supplementary material for: Plant pectin acetylesterase structure and function: new insights from bioinformatic analysis
Source: BMC Genomics. 2017 Jun 8;18:456. doi: 10.1186/s12864-017-3833-0 (PMC5465549; doi:10.1186/s12864-017-3833-0)
Supplement: Supplementary file 5 — Pairwise sequence identity (a) and similarity (b) from multiple sequence alignment of AtPAE proteins without their signal peptide using Muscle [36]. Percentage identity and similarity of amino acid residues were determined using SIAS (http://imed.med.ucm.es/Tools/sias.html). (PDF 28.3 kb) [file 12864_2017_3833_MOESM5_ESM.pdf]

Additional file 5.

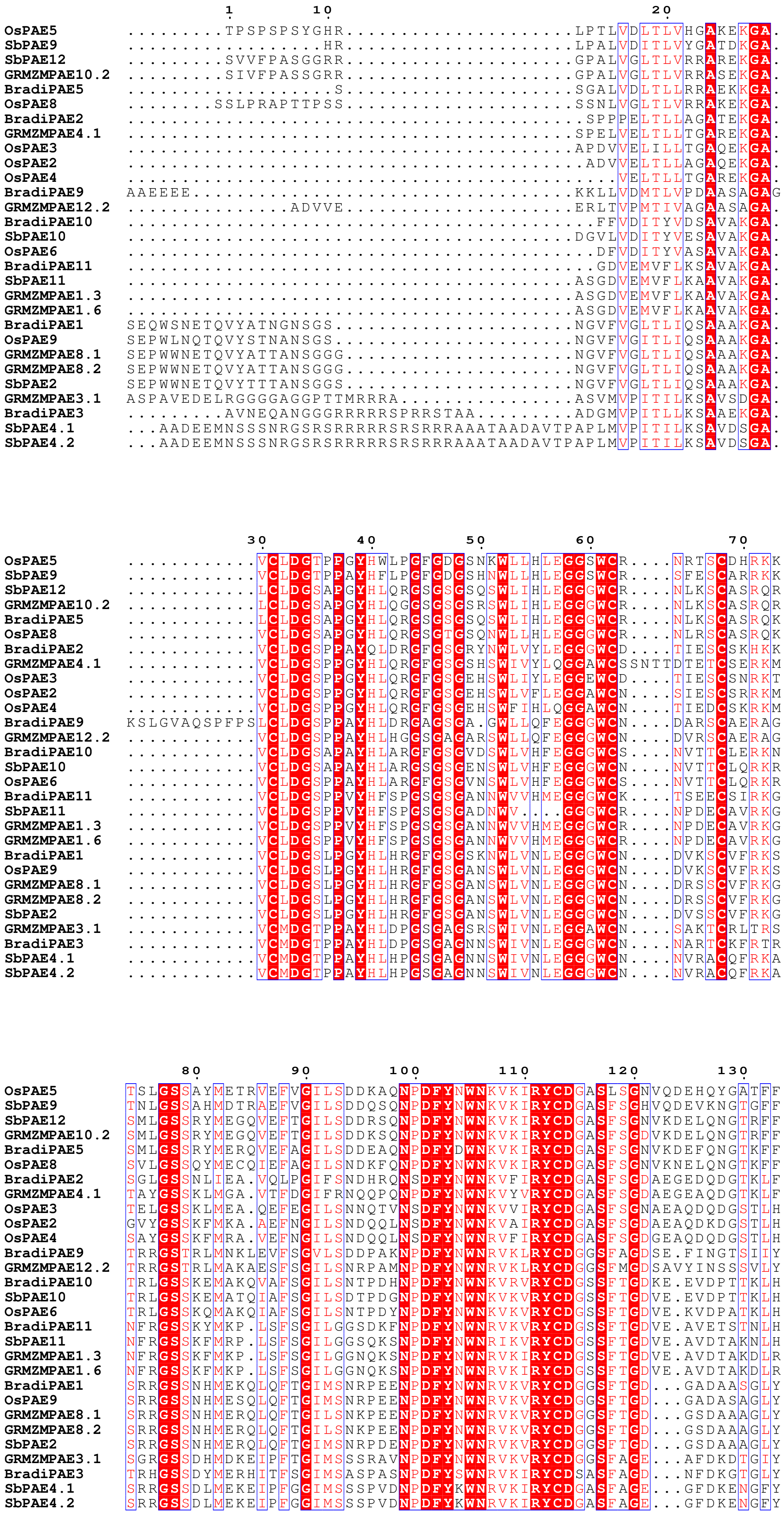
1 10 20
OsPAE5 .........TPSPSPSYGHR....................LPTLVDLTLVHGAKEKGA.
SbPAE9 ...............HR....................LPALVDITLVYGATDKGA.
SbPAE12 .........SVVFPASGGRR....................GPALVGLTLVRRAREKGA.
GRMZMPAE10.2 .........SIVFPASSGRR....................GPALVGLTLVRRASEKGA.
BradiPAE5 ................S....................SGALVDLTLLRRAEKKGA.
OsPAE8 ........SSLPRAPTTPSS....................SSNLVGLTLVRRAKEKGA.
BradiPAE2 ....SPPPELTLLAGATEKGA.
GRMZMPAE4.1 ....SPELVELTLLTGAREKGA.
OsPAE3 ....APDVVELILLTGAQEKGA.
OsPAE2 ....ADVVELTLLAGAQEKGA.
OsPAE4 ....VELTLLTGAREKGA.
BradiPAE9 AAEEEE....................................KKLLVDMTLVPDAASAGAG
GRMZMPAE12.2 ...............ADVVE....................ERLTVPMTIVAGAASAGA.
BradiPAE10 ....FFVDITYVDSAVAKGA.
SbPAE10 ....DGVLVDITYVESAVAKGA.
OsPAE6 ....DFVDITYVASAVAKGA.
BradiPAE11 ....GDVEMVFLKSAVAKGA.
SbPAE11 ....ASGDVEMVFLKAAVAKGA.
GRMZMPAE1.3 ....ASGDVEMVFLKAAVAKGA.
GRMZMPAE1.6 ....ASGDVEMVFLKAAVAKGA.
BradiPAE1 SEQWSNETQVYATNGNSGS.......................NGVFVGLTLIQSAAAKGA.
OsPAE9 SEPWLNQTQVYSTNANSGS.......................NGVFVGITLIQSAAAKGA.
GRMZMPAE8.1 SEPWWNETQVYATTANSGGG......................NGVFVGLTLIQSAAAKGA.
GRMZMPAE8.2 SEPWWNETQVYATTANSGGG......................NGVFVGLTLIQSAAAKGA.
SbPAE2 SEPWWNETQVYTTTANSGGS......................NGVFVGLTLIQSAAAKGA.
GRMZMPAE3.1 ASPAVEDELRGGGGAGGPTTMRRRA.................ASVMVPITILKSAVSDGA.
BradiPAE3 .........AVNEQANGGGRRRRRSPRRSTAA.........ADGMVPITLLKSAAEKGA.
SbPAE4.1 ...AADEEMNSSSNRGSRSRRRRRRSRSRRRAAATAADAVTPAPLMVPITILKSAVDSGA.
SbPAE4.2 ...AADEEMNSSSNRGSRSRRRRRRSRSRRRAAATAADAVTPAPLMVPITILKSAVDSGA.

30 40 50 60 70
OsPAE5 ...........VCLDGTPPGYHWLPGFGDGSNKWLLHLEGGSWCR....NRTSCDHRKK
SbPAE9 ...........VCLDGTPPAYHFLPGFGDGSHNWLLHLEGGSWCR....SFESCARRKK
SbPAE12 ...........LCLDGSAPGYHLQRGSGSGSQSWLIHLEGGGWCR....NLKSCASRQR
GRMZMPAE10.2 ...........LCLDGSAPGYHLQGGSGSGSRSWLIHLEGGGWCR....NLKSCASRQR
BradiPAE5 ...........LCLDGSAPGYHLQRGSGSGSQSWLIHLEGGGWCR....NLKSCASRQK
OsPAE8 ...........VCLDGSAPGYHLQRGSGTGSQNWLLHLEGGGWCR....NLRSCASRQK
BradiPAE2 ...........VCLDGSPPAYQLDRGFGSGRYNWLVYLEGGGWCD....TIESCSKHKK
GRMZMPAE4.1 ...........VCLDGSPPGYHLQRGFGSGSHSWIVYLQGGAWCSSNTTDTETCSERKM
OsPAE3 ...........VCLDGSPPGYHLQRGFGSGEHSWLIYLEGGEWCD....TIESCSNRKT
OsPAE2 ...........VCLDGSPPGYHLQRGFGSGEHSWLVFLEGGAWCN....SIESCSRRKM
OsPAE4 ...........VCLDGSPPGYHLQRGFGSGEHSWFIHLQGGAWCN....TIEDCSKRKM
BradiPAE9 KSLGVAQSPFPSLCLDGSPPAYHLDRGAGSGA.GWLLQFEGGGWCN....DARSCAERAG
GRMZMPAE12.2 ...........VCLDGSPPAYHLHGGSGAGARSWLLQFEGGGWCN....DVRSCAERAG
BradiPAE10 ...........VCLDGSAPAYHLARGFGSGVDSWLVHFEGGGWCS....NVTTCLERKN
SbPAE10 ...........VCLDGSAPAYHLARGSGSGENSWLVHFEGGGWCN....NVTTCLQRKR
OsPAE6 ...........VCLDGSPPAYHLARGFGSGVNSWLVHFEGGGWCS....NVTTCLQRKR
BradiPAE11 ...........VCLDGSPPVYHFSPGSGSGANNWVVHMEGGGWCK....TSEECSIRKG
SbPAE11 ...........VCLDGSPPVYHFSPGSGSGADNWV....GGGWCR....NPDECAVRKG
GRMZMPAE1.3 ...........VCLDGSPPVYHFSPGSGSGANNWVVHMEGGGWCR....NPDECAVRKG
GRMZMPAE1.6 ...........VCLDGSPPVYHFSPGSGSGANNWVVHMEGGGWCR....NPDECAVRKG
BradiPAE1 ...........VCLDGSLPGYHLHRGFGSGSKNWLVNLEGGGWCN....DVKSCVFRKS
OsPAE9 ...........VCLDGSLPGYHLHRGFGSGANSWLVNLEGGGWCN....DVKSCVFRKS
GRMZMPAE8.1 ...........VCLDGSLPGYHLHRGFGSGANSWLVNLEGGGWCN....DRSSCVFRKG
GRMZMPAE8.2 ...........VCLDGSLPGYHLHRGFGSGANSWLVNLEGGGWCN....DRSSCVFRKG
SbPAE2 ...........VCLDGSLPGYHLHRGFGSGANSWLVNLEGGGWCN....DVSSCVFRKG
GRMZMPAE3.1 ...........VCMDGTPPAYHLDPGSGAGSRSWIVNLEGGAWCN....SAKTCRLTRS
BradiPAE3 ...........VCMDGTPPAYHLDPGSGAGNNSWIVNLEGGGWCN....NARTCKFRTR
SbPAE4.1 ...........VCMDGTPPAYHLHPGSGAGNNSWIVNLEGGGWCN....NVRACQFRKA
SbPAE4.2 ...........VCMDGTPPAYHLHPGSGAGNNSWIVNLEGGGWCN....NVRACQFRKA

80 90 100 110 120 130
OsPAE5 TSLGSSAYMETRVEFVGILSDDKAQNPDFYNWNKVKIRYCDGASLSGNVQDEHQYGATFF
SbPAE9 TNLGSSAHMDTRAEFVGILSDDQSQNPDFYNWNKVKIRYCDGASFSGHVQDEVKNGTGFF
SbPAE12 SMLGSSRYMEGQVEFTGILSDDRSQNPDFYNWNKVKIRYCDGASFSGNVKDELQNGTRFF
GRMZMPAE10.2 SMLGSSRYMEGQVEFTGILSDDKSQNPDFYNWNKVKIRYCDGASFSGDVKDELQNGTRFF
BradiPAE5 SMLGSSRYMERQVEFAGILSDDEAQNPDFYDWNKVKIRYCDGASFSGNVKDEFQNGTKFF
OsPAE8 SVLGSSQYMECQIEFAGILSNDKFQNPDFYNWNKVKIRYCDGASFSGNVKNELQNGTKFF
BradiPAE2 SGLGSSNLIEA.VQLPGIFSNDHRQNSDFYNWNKVFIRYCDGASFSGDAEGEDQDGTKLF
GRMZMPAE4.1 TAYGSSKLMGA.VTFDGIFRNQQPQNPDFYNWNKVYVRYCDGASFSGDAEGEAQDGTKLF
OsPAE3 TELGSSKLMEA.QEFEGILSNNQTVNSDFYNWNKVVIRYCDGASFSGNAEAQDQDGSTLH
OsPAE2 GVYGSSKFMKA.AEFNGILSNDQQLNSDFYNWNKVAIRYCDGASFSGDAEAQDKDGSTLH
OsPAE4 SAYGSSKFMRA.VEFNGILSNDQQLNSDFYNWNRVFIRYCDGASFSGDGEAQDQDGSTLH
BradiPAE9 TRRGSTRLMNKLEVFSGVLSDDPAKNPDFYNWNRVKLRYCDGGSFAGDSE.FINGTSIIY
GRMZMPAE12.2 TRRGSTRLMAKAESFSGILSNRPAMNPDFYNWNRVKLRYCDGGSFMGDSAVYINSSSVLY
BradiPAE10 TRLGSSKEMAKQVAFSGILSNTPDHNPDFYNWNKVRVRYCDGSSFTGDKE.EVDPTTKLH
SbPAE10 TRLGSSKEMATQIAFSGILSDTPDGNPDFYNWNKVKVRYCDGSSFTGDVE.EVDPTTKLH
OsPAE6 TRLGSSKQMAKQIAFSGILSNTPDYNPDFYNWNKVKVRYCDGSSFTGDVE.KVDPATKLH
BradiPAE11 NFRGSSKYMKP.LSFSGILGGSDKFNPDFYNWNRVKVRYCDGSSFTGDVE.AVETSTNLH
SbPAE11 NFRGSSKFMRP.LSFSGILGGSQKSNPDFYNWNRIKVRYCDGSSFTGDVE.AVDTAKNLH
GRMZMPAE1.3 NFRGSSKFMKP.LSFSGILGGNQKSNPDFYNWNRVKIRYCDGSSFTGDVE.AVDTAKDLR
GRMZMPAE1.6 NFRGSSKFMKP.LSFSGILGGNQKSNPDFYNWNRVKIRYCDGSSFTGDVE.AVDTAKDLR
BradiPAE1 SRRGSSNHMEKQLQFTGIMSNRPEENPDFYNWNRVKVRYCDGGSFTGD...GADAASGLY
OsPAE9 SRRGSSNHMESQLQFTGIMSNRPEENPDFYNWNRVKVRYCDGGSFTGD...GADASAGLY
GRMZMPAE8.1 SRRGSSNHMERQLQFTGILSNKPEENPDFYNWNRVKVRYCDGGSFTGD...GSDAAAGLY
GRMZMPAE8.2 SRRGSSNHMERQLQFTGILSNKPEENPDFYNWNRVKVRYCDGGSFTGD...GSDAAAGLY
SbPAE2 SRRGSSNHMERQLQFTGIMSNRPDENPDFYNWNRVKVRYCDGGSFTGD...GSDAAAGLY
GRMZMPAE3.1 SGRGSSDHMDKEIPFTGIMSSSRAVNPDFYNWNRVKVRYCDGGSFAGE...AFDKDTGIY
BradiPAE3 TRHGSSDYMERHITFSGIMSASPASNPDFYSWNRVKIRYCDSASFAGD...NFDKGTGLY
SbPAE4.1 SRRGSSDLMEKEIPFGGIMSSSPVDNPDFYKWNRVKIRYCDGASFAGE...GFDKENGFY
SbPAE4.2 SRRGSSDLMEKEIPFGGIMSSSPVDNPDFYKWNRVKIRYCDGASFAGE...GFDKENGFY

```
                        140       150       160       170       180       190
OsPAE5        FRGQRIWEAVMAELLP.KGLARAKQAFLTGCSAGGLSTYIHCDDFRALLPKDST.VKCLA
SbPAE9        FRGQRIWEAVMAELLS.KGLARAKQAFLTGCSAGGLSTYIHCDDFRAVLPNTPT.VKCLA
SbPAE12       FRGQRIWEAVMNELVV.KGLRNAKQAFLTGCSAGGLATYIHCDSFRALLPKDSR.VKCLA
GRMZMPAE10.2  FRGQRIWEAVMNELVV.KGLRNAKQAFLTGCSAGGLATYIHCDSFRALLPKDSR.VKCLA
BradiPAE5     FRGQRIWKAVMDELLL.KGLKHAKQAFLTGCSAGGLATYIHCDDFRALLPKDSR.VKCLA
OsPAE8        FRGQRIWEAVMSELLL.KGLRHAKQAFLTGCSAGGLATFIHCDNFRTLLPKDSR.VKCLA
BradiPAE2     FRGLRIWEAVIDELME.KGLANAKQALLAGCSSGGLAVLLHCDNFSARFPQTVP.VKCFS
GRMZMPAE4.1   FRGSRIWDAVVDELMG.KGMDAAEQALLAGCSAGGLATLLHCDDFRARFPQEVP.VKCLP
OsPAE3        FRGLRIWQAVLDELME.KGLASAKQALLSGCSAGGLATLLHCNDFHARFPKEVS.AKCLP
OsPAE2        FRGLRIWEAVVDELMG.KGLATAKQAILSGCSAGGLAALLHCNDFHARFPKEVS.AKCLP
OsPAE4        FRGLRIWEAVINELMG.KGLATAKQAILSGCSAGGLAALLHCNDFYARFSKEVS.AKCLP
BradiPAE9     MRGQRIWDAIITDLFR.KGLATAQQVLLSGCSAGGLATFFHCDDLQERLGGATT.....
GRMZMPAE12.2  FSGQRIWDAIVADLLR.KGLARADKVLLSGCSAGGLATFFHCDGLKQRLGAAAT.VKCLS
BradiPAE10    YRGARVWQAVMEDLLA.KGMDRAENALISGCSAGGLTSVLHCDRFRDRMPVEAN.VKCLS
SbPAE10       FRGARIWQAVMEDLLA.KGMDKAENALISGCSAGGLTSILHCDRFHDLLPLAAR.VKCLS
OsPAE6        YRGARVWQAVMDDLLA.KGMNSANNALISGCSAGGLTSILHCDRFRDLFPVDTK.VKCLS
BradiPAE11    YRGNRVWQAIIQDLLD.RGMSKAQNALLSGCSAGGLAAILHCDRFSDLLPASAK.VKCFS
SbPAE11       YRGFRVWRAIMDDLLTVRGMNKAKYALLSGCSAGGLAAILHCDRFRDLFPATTK.VKCFS
GRMZMPAE1.3   YRGFRVWRAVIDDLLTVRGMSKAQNALLSGCSAGGLAAILHCDRFHDLFPAKTK.VKCFS
GRMZMPAE1.6   YRGFRVWRAVIDDLLTVRGMSKAQNALLSGCSAGGLAAILHCDRFHDLFPAKTK.VKCFS
BradiPAE1     FRGQRIWQAAMDDLMS.QGMRSASQALLSGCSAGGASTILHCDEFRGLFPSNTR.VKCLA
OsPAE9        FRGQRIWQAAMDDLMA.QGMRYANQALLSGCSAGGVSTILHCDEFRGLFSGSTN.VKCLA
GRMZMPAE8.1   FRGQRIWQAAMDDLMA.QGMRYANQALLSGCSAGGVSTILHCDEFHGLFPSNTR.VKCLA
GRMZMPAE8.2   FRGQRIWQAAMDDLMA.QGMRYANQALLSGCSAGGVSTILHCDEFHGLFPSNTR.VKCLA
SbPAE2        FRGQRIWQAAMDDLMA.QGMRYANQALLSGCSAGGVSTILHCDEFRGLFPSNTR.VKCLA
GRMZMPAE3.1   FRGQRIWNAVIRHLLS.IGMANADQVLLAGCSSGGLAVILHCDQLRAFFPSGSTVVKCIS
BradiPAE3     FRGQRIWDAAIQHLLS.IGMASADQVLLTGCSAGGLAAILHCDQFSAFFAGKNTTVKCLA
SbPAE4.1      FRGQRIWDATVRHLLS.IGMASAEQVLLTGCSAGGLAVILHCDQFQAFFPRSTTVVKCLA
SbPAE4.2      FRGQRIWDATVRHLLS.IGMASAEQVLLTGCSAGGLAVILHCDQFQAFFPRSTTVVKCLA
```

```
                                  200       210       220       230
OsPAE5        DGGFFLDV......EDISGRRYMRGFYNDVARQQDLRKRFPG.CSSD..M...EPG....
SbPAE9        DGGFFLDV......EDISGRRYMRGFYNDVARLQDVHKRFPH.CSSD..M...EPG....
SbPAE12       DGGFFLDV......EDISGRRTMQSFYSDVVRLQGLRERFSH.CNSN..M...EAG....
GRMZMPAE10.2  DGGFFLDV......EDISGRRTMHSFYSDIVRLQGLRERFSH.CNSN..M...DAG....
BradiPAE5     DGGFFLDV......EDISKQRTLRAFYSEVVRLQDLKRRFLH.CSSS..E...DPG....
OsPAE8        DGGFFLDV......EDISGQRTMRAFYNDVVRLQDLRGRFPH.CGPN..M...DLG....
BradiPAE2     DAGFFLDI......KDISGERFIRSVFSGVVHLQNVRKVLPKDCLAK..K...EPT....
GRMZMPAE4.1   DGGFFLDI......KDLSGERHMRSVFSGVVQLQNVSGVLPKRCLAK..K...DPAEARS
OsPAE3        DAGIFLDI..LCSSEDLSGKRLMWSVFNGTVQLQNVSEVLPKDCLAK..K...VRT....
OsPAE2        DAGFFLDV..FCSSEDLSGERHMWSVFNGTVHLQNVREVLSKDCLTK..K...DPT....
OsPAE4        DAGFFLDIASFCSSEDLSGERHMWSVFNGTVHLQNVTQVLSKDCLAK..K...DPT....
BradiPAE9     ..................................GAQKNLNKECLNS..ML..YPY....
GRMZMPAE12.2  DAGFFLDL......SDISGSNTIRQFFSSLVSLQGIQKNLNMDCLSS..TSTDNAY....
BradiPAE10    DAGFFIDV......KDIAGEKHAADFFNDVVTTHGSAKNLPSSCTSK..L...PPG....
SbPAE10       DAGFFINE......KDVAGVGYIAAFFNDVVTTHGSANNLPSPCTSM..L...PPG....
OsPAE6        DAGFFINE......KDIAGVEYIVAFFNGVATTHGSAKNLPSACTSR..L...SPG....
BradiPAE11    DAGYFFDG......TDITGNNYVRKSYKDIVNLHGSAKSLPSSCTSK..R...SPE....
SbPAE11       DAGYFFDG......KDISGNYYARSIYKNVVNLHGSAKNLPASCTSK......QPE....
GRMZMPAE1.3   DAGYFFDG......KDISGNFYARSIYKSVVNLHGSAKNLPASCTSKPKQ...SPE....
GRMZMPAE1.6   DAGYFFDG......KDISGNFYARSIYKSVVNLHGSAKNLPASCTSKPKQ...SPE....
BradiPAE1     DAGMFLDT......VDVAGRREMREFFNGIVRLQGSGRSLPRSCTSR..M...DKT....
OsPAE9        DAGMFLDF......VDVSGQREMRDFFNGIVRLQGSGRSLPRSCTSR..M...DKT....
GRMZMPAE8.1   DAGMFLDT......VDVSGRREMRSFFNGIVRLQGSGRSLPRSCTSH..M...DKT....
GRMZMPAE8.2   DAGMFLDT......VDVSGRREMRSFFNGIVRLQGSGRSLPRSCTSH..M...DKT....
SbPAE2        DAGMFLDT......VDVSGRREMRSFFNGIVRLQGSGRSLPRSCTAR..M...DKT....
GRMZMPAE3.1   DGGLYLDA......VDVSGGRSLRSYFGDIVAMQGIAQNLPPACTAR..L...DAT....
BradiPAE3     DAGLFLDA......LDVSGGRSLRSYYGEIVAMQEVARNLPPSCTGH..L...DAT....
SbPAE4.1      DAGLFLDA......SDVSGGRSLRSYYSDIVAMQGVAPNLPPACTAR..L...DTT....
SbPAE4.2      DAGLFLDA......SDVSGGRSLRSYYSDIVAMQGVAPNLPPACTAR..L...DTT....
```

```
                                             240       250       260
OsPAE5        ...........................QCFFPQEVAKGITTPMFILNPAYDVWQVEHVLT
SbPAE9        ...........................QCFFPQEVAKSITTPMFILNPAYDVWQVEHVLS
SbPAE12       ...........................QCLFPREVVKHIVNPVFVLNPAYDAWQVQHALA
GRMZMPAE10.2  ...........................QCFFPREVVKHIVNPVFVLNPAYDAWQVQHALA
BradiPAE5     ...........................QCFFPREVVKAIHTPVFVLNPAYDAWQVQHVLA
OsPAE8        ...........................QCFFPSEVVKDIITPVFVLNPAYDAWQVQHVLS
BradiPAE2     ...........................DCFFPAEVIKSINTPTFILNSGYDSWQIQNVLV
GRMZMPAE4.1   CSLDLYDIFASPFNMLSCQRFVFLDLRQCFFPAELIKSISTPTFIVNSEYDSWQIANVVA
OsPAE3        ...........................ECFLATELVKSITAPTLIVNSAYDSWQIRDTLA
OsPAE2        ...........................ECFFPAELVKSITAPTLILNSAYDSWQIRNALA
OsPAE4        ...........................ECFFPAELVKSITAPTLILNSAYDSWQIQNALA
BradiPAE9     ...........................QCFFPQYALQNIRTPYFILNSAYDVYQFHHTFV
GRMZMPAE12.2  ...........................LCFFPQFALANIRTPFFILNSAYDVYQFHHILV
BradiPAE10    ...........................MCLFPQNEVKQIQTPLFILNAAYDSWQVRNILV
SbPAE10       ...........................MCFFPKNEVKQIHTPLFILNAAYDSWQVRNILV
OsPAE6        ...........................MCFFPQNEVKQIQTPLFILNAAYDSWQVRNILV
BradiPAE11    ...........................LCFFPQYVIPTLRTPLFILNAAYDTWQIRNILA
SbPAE11       ...........................LCMFPQYVVPTMRTPLFILNAAYDSWQVKNVLA
GRMZMPAE1.3   ...........................LCMFPQYVVPTMRTPLFILNAAYDSWQVKNVLA
GRMZMPAE1.6   ...........................LCMFPQYVVPTLRTPLFILNAAYDSWQVKNVLA
BradiPAE1     ...........................SCFFPQNVLPNIQTPTFILNTAYDVWQLQQSVA
OsPAE9        ...........................SCFFPQNVVPNIQTPTFILNTAYDVWQLQQSVA
GRMZMPAE8.1   ...........................SCFFPQNVLPTIRTPTFVLNTAYDVWQLQQSVA
GRMZMPAE8.2   ...........................SCFFPQNVLPTIRTPTFVLNTAYDVWQLQQSVA
SbPAE2        ...........................SCFFPQNVLPNIQTPTFVLNTAYDVWQLQQSVA
GRMZMPAE3.1   ...........................SCFFPQNIIDGVKTPLFLLNAAYDFIQIVLSLA
BradiPAE3     ...........................SCFFPQNVIDSIKTPIFLLNAAYDAWQIEESLA
SbPAE4.1      ...........................SCFFPQNVIDGINTPIFLLNAAYDVWQIQESLA
SbPAE4.2      ...........................SCFFPQNVIDGINTPIFLLNAAYDVWQIQESLA
```

```
             270       280       290       300       310       320
OsPAE5       PDGSDPQNLWQDCRMDITKCNTKQLEILQGFRKSLLDAI.SEFKKKRGWGMFIDSCFIHC
SbPAE9       PEGSDPQNLWQNCRTDITKCSSKQLEVLQGFRKALLDAI.NEFKKRRDWGMFIDSCFIHC
SbPAE12      PEASDPQHSWLDCRLDISKCGSEQLEILQGFRKELHDAI.SEVKQKRDWGFYINSCFVHC
GRMZMPAE10.2 PEASDPQHSWLDCRLDISKCSPKQLGILQGFRKELHDAI.SEAKQKRGWGFYINSCFVHC
BradiPAE5    PEASDPKHSWLDCRLDISKCNPNQLKILQGFREELHVAM.SELKQKKDWGIFIDSCFVHC
OsPAE8       PVASDPQHSWLECRLDISKCDSNQLEILQGFRKKLHDTI.SELKHKKDWGFFIDSCFIHC
BradiPAE2    PDETSPEKSWLTCKANIRECNPTQIEALHGFRETLVNDL.KVVQDKEDWGLFIDSCFTHC
GRMZMPAE4.1  PDGSYTGDAWTSCRDNIRNCSSEQMDVLHGFRAELIREL.KVAEGEREWGLFVDSCFTHC
OsPAE3       PVGSYPGQSWLNCTNDIGNCNSTQMEVLNGFRKKFVDGV.KVVKDKKDWGLFIDSCFMHC
OsPAE2       PDGSFPGQSWSSCKTDIRNCSSTQIQVFNGFRNKFVDDV.EIVKDKKDWGLFIDSCFTHC
OsPAE4       PDGSFPGQSWSSCKTDIRNCSSTQIQVFNGFRNKFVDDI.EVVKDKKDWGLFIDSCFTHC
BradiPAE9    PPSCDPRGQWSRCKSDPAACSTSQIATLQGLRNAMLTAL.NLFEGDSKVGMFINSCFAHC
GRMZMPAE12.2 PPSSDPGGHWSRCKSDPGGCNATQIATLQGLRSGMLTSL.RQFKSKPEAGMFINSCFAHC
BradiPAE10   PGGSDPH..WRSCKHDINQCSEKQLKTLQGFRDDFLKAL.EEQGSSSSRGLFINSCFAHC
SbPAE10      PGVADPHGKWHSCKHDIGQCSASQLRVLQGFRGDFLKEV.SEQANSDSRGLFINSCFVHC
OsPAE6       PGFADPHGKWHSCKHDIDQCPASQLQILQGFRDDFLKAL.KEQGTPSTRGLFINSCFVHC
BradiPAE11   PNAADPKKTWAKCKLDIKSCSSSQLVTLQNFRKDFLAAL.PQPGQSPSLGIFIDSCFAHC
SbPAE11      PSPADPKKTWAQCKLDIKSCSPSQLTTLQNFRTDFLAAL.P...KTPSVGMFIDSCNAHC
GRMZMPAE1.3  PSPADPKKTWAQCKLDIKSCSASQLTTLQNFRTDFLAAL.P...KTQSVGMFIDSCNAHC
GRMZMPAE1.6  PSPADPKKTWAQCKLDIKSCSASQLTTLQNFRTDFLAAL.P...KTQSVGMFIDSCNAHC
BradiPAE1    PKTADPQGLWQRCKQNHAFCSGNQLQFLNGFRNEMLDAV.KGFSGSRQNGVFINSCFAHC
OsPAE9       PKRADPQGLWRGCRMNHASCNSNQLQFLQGFRNQMLDAV.RGFSGARQNGLFINSCFAHC
GRMZMPAE8.1  PRTADPQGLWSKCRTNHAFCNSNQLQFLQGFRNQMLDAV.RGFSASRQNGLFINSCFAHC
GRMZMPAE8.2  PRTADPQGLWSKCRTNHAFCNSNQLQFLQGFRNQMLDAV.RGFSASRQNGLFINSCFAHC
SbPAE2       PRTADPQGLWSKCRTNHAFCNSNQLQFLQGFRNQMLDAV.KGFSASRRNGLFINSCFAHC
GRMZMPAE3.1  PDRADPSGAWRACKSNRTACSASQMSFLQDFRDQMVASV.KGFSGSRSNGVFLSSCFAHC
BradiPAE3    PNRADPSGAWRACKYNRSACDASQIKFLQSFRDQMVASV.KAFSGSRSNGLFINSCFAHC
SbPAE4.1     PTGADPSGAWRACKSNHSACDASQMKFLQDFRDQMVASVNNGFAGSRSNGLFINSCFAHC
SbPAE4.2     PTGADPSGAWRACKSNHSACDASQMKFLQDFRDQMVASVNNGFAGSRSNGLFINSCFAHC

              330                 340       350       360            370
OsPAE5       QSMKSLAWHSP.....SASRINNKTVAEAVGDWFFDRRE..VKEIDCEYPCNPTCFNVV.
SbPAE9       QSMKALAWHSP.....SAARINNKTAAEAVGDWFFDRRE..VKEIDCEYPCNPTCYNVV.
SbPAE12      QSLNSLTWHSP.....TSPRVNNKSIAEAVGDWFFDRRE..VKEIDCEYPCNPTCHNLV.
GRMZMPAE10.2 QSLNSLTWHSP.....TSPRVNNKSIAEAVGDWFFDRRE..VKEIDCEYPCNPTCHNLV.
BradiPAE5    QSLNSLTWHSP.....SSPRVSNKTIAEAVGDWFFDRRE..VKELDCEYPCNPTCHNLV.
OsPAE8       QSLNSLTWHSP.....SSLRVNNKTIAEAVGDWFFDRRE..VKEIDCEYPCNPTCHNLV.
BradiPAE2    QTPFRISWDSP.....ISPRLQNKSIAEAVGDWHFGRSRSGVKQIDCEYPCNPTCSTQL.
GRMZMPAE4.1  QTQSSDWWHSP.....TSPRLGNQTVAEAVGDWYFGRRRV.VKQVDCEYPCNPTCSIQ..
OsPAE3       QTKYSISWSSQ.....FSPVLGNMTIAKAVGDWYFERSKT.VKEIDCEYPCNPTCKLTG.
OsPAE2       QTPFNISWSSQ.....ASPVLGSKTVAEAVGDWYFERSYE.VKEIDCEYPCNPTCSSQL.
OsPAE4       QTPFDISWNSQ.....ASPVLGNKIVAEAIGDWYFERSYE.VKEIDCEYPCNPTCSSQL.
BradiPAE9    QSELQDTWFAP.....NSPSLHNKTIAELVGDWYFERGA..AQEIDCAYPCDLTCHNIIP
GRMZMPAE12.2 QSELQDTWFAP.....NSPSIDNKKIAEVVGDWYFERGA..AVEIDCAYPCDSTCRNLI.
BradiPAE10   QSEIQEIWFAP.....DSPVLGNKKIANAIGDWFYDRSP..FQEIDCPYPCDSSCH..V.
SbPAE10      QSESQELWFSS.....DSPKLGNTTIANAVGDWFFGRSS..FQKIDCPYPCDSTCHNGI.
OsPAE6       QSETQETWFAS.....GSPMLETKTIADAVGDWFYDRNP..FQKIDCPYPCDSTCHNRI.
BradiPAE11   QSGAQDTWIGE.....GSPSIQKMRIGKAVGDWFYNRHV..SQLIDCPYPCNPTCKN...
SbPAE11      QSGSQDTWLAD.....GSPTVNKTQIGKAVGDWFYDREV..SRQIDCPYPCNPTCKN...
GRMZMPAE1.3  QSGSQDTWLAD.....GSPTVNKTQIGKAVGDWYYDREV..PRQIDCPYPCNPTCKN...
GRMZMPAE1.6  QSGSQDTWLAD.....GSPTVNKTQIGKAVGDWYYDREV..PRQIDCPYPCNPTCKN...
BradiPAE1    QSERQDTWYSN.....NSPRLGNRRIAEAVGDWFFERGD..AKYTDCTYPCDGTCHHLV.
OsPAE9       QSERQDTWYAG.....DSPRLGNKRIAEAVGDWFFDRAD..AKYTDCAYPCDGTCHHLT.
GRMZMPAE8.1  QSERQDTWYAN.....NSPRLGNKKIADAVGDWFFERGN..AKYTDCPYPCDGTCHHLV.
GRMZMPAE8.2  QSERQDTWYAN.....NSPRLGNKKIADAVGDWFFERGN..AKYTDCPYPCDGTCHHLV.
SbPAE2       QSERQDTWYAN.....NSPRLGNKKIADAVGDWFFERGD..AKYTDCPYPCDGTCHHLV.
GRMZMPAE3.1  QSEQLGTWNTKP...GGSPTIQNKGISKSVGDWYFDRAE..VKAVDCRYPCDNTCHHII.
BradiPAE3    QSELPATWNDAP....GSPAVQNKGIAKSVGDWYFGRAE..VKAIDCPYPCDNTCRHII.
SbPAE4.1     QSELPMTWSDNAAGGGASPAIQSRGIAKSVGDWYFGRAQ..VKAIDCPYPCDRTCRNII.
SbPAE4.2     QSELPMTWSDNAAGGGASPAIQSRVTCNAAIHIYTH.....IKAIK..............

                 380
OsPAE5       ....LEQPYQEG..
SbPAE9       ....LDQPYKED..
SbPAE12      ....FAKAFKI...
GRMZMPAE10.2 ....FARPFKI...
BradiPAE5    ....FDKPFKG...
OsPAE8       ....FAKPFKA...
BradiPAE2    ....PS........
GRMZMPAE4.1  ..............
OsPAE3       ..............
OsPAE2       ....PT........
OsPAE4       ....PK........
BradiPAE9    TGYPFDRVKEIPEF
GRMZMPAE12.2 ....PIDKNGFAGA
BradiPAE10   ....FKNSSET...
SbPAE10      ....YEDSSQA...
OsPAE6       ....YDDPSEA...
BradiPAE11   ........REED..
SbPAE11      ........REDD..
GRMZMPAE1.3  ........RDDD..
GRMZMPAE1.6  ........RDDD..
BradiPAE1    ....F...RGRGL.
OsPAE9       ....F...RGDY..
GRMZMPAE8.1  ....F...RGDH..
GRMZMPAE8.2  ....F...RGDH..
SbPAE2       ....F...RGDH..
GRMZMPAE3.1  ..............
BradiPAE3    ..............
SbPAE4.1     ..............
SbPAE4.2     ..............
```
