# Supplementary material for: Plant pectin acetylesterase structure and function: new insights from bioinformatic analysis
Source: BMC Genomics. 2017 Jun 8;18:456. doi: 10.1186/s12864-017-3833-0 (PMC5465549; doi:10.1186/s12864-017-3833-0)

Additional file 6.

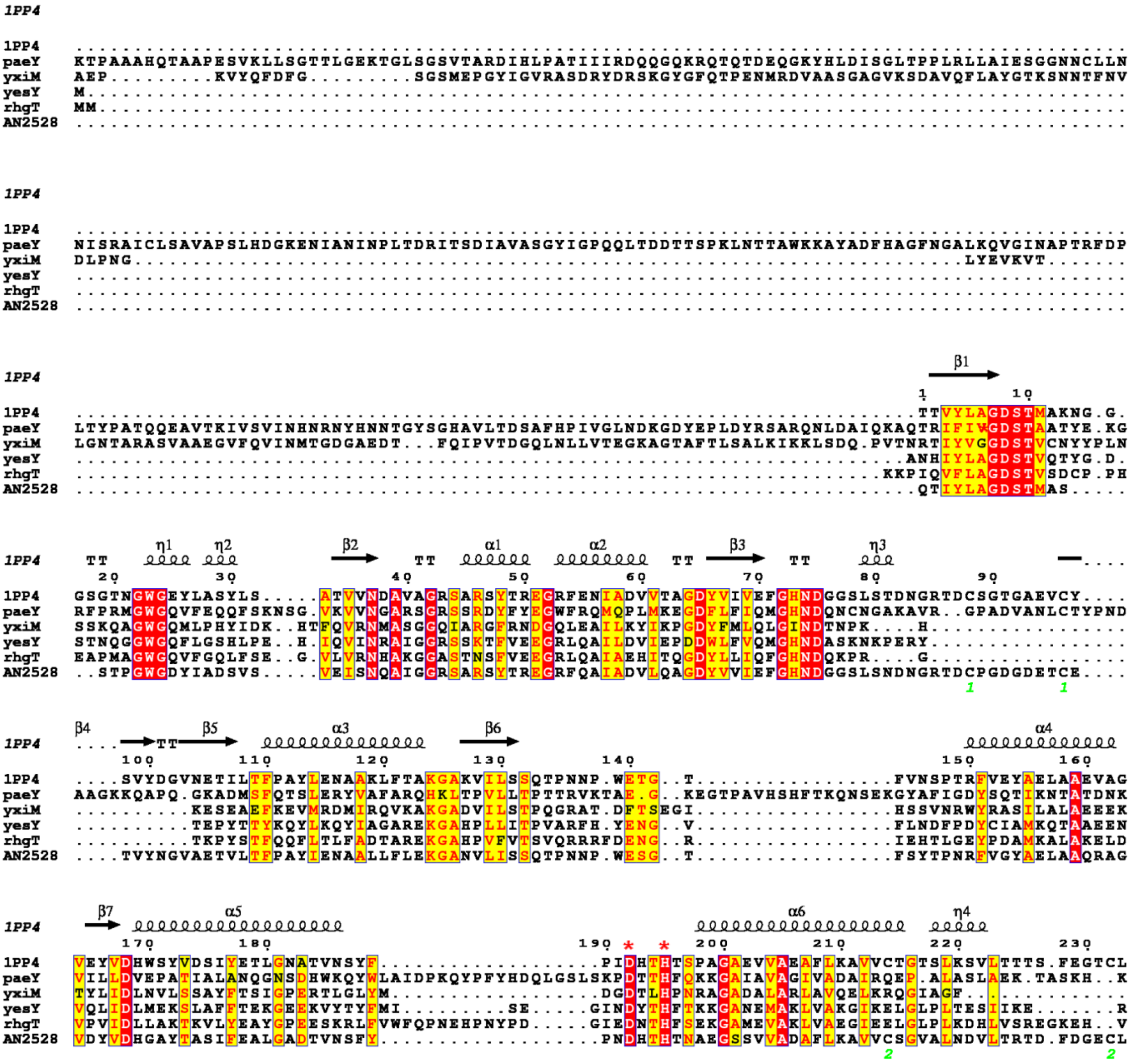

Supplement: Supplementary file 6 — 3D homology modeling of AtPAE8 with a rhamnogalacturonan acetylesterase from Aspergillus aculeatus. (a) AtPAE8 3D model threaded with an AacRGAE (PDB code: 1DEO) using FUGUE [75]. (b) Structure of AacRGAE [28]. (c) Pairwise structure alignment between AtPAE8 and AacRGAE using T-coffee and rendered with ESPript3 [73]. (PDF 394 kb) [file 12864_2017_3833_MOESM6_ESM.pdf]
