# Supplementary material for: Plant pectin acetylesterase structure and function: new insights from bioinformatic analysis
Source: BMC Genomics. 2017 Jun 8;18:456. doi: 10.1186/s12864-017-3833-0 (PMC5465549; doi:10.1186/s12864-017-3833-0)
Supplement: Supplementary file 14 — Structure sequence alignment of AtPAEs with a carbohydrate acetylesterase from kiwi (AeCXE1, PDB code: 2O7R). The AtPAEs and the kiwi carbohydrate acetylesterase (AeCXE1) sequences without their signal peptide were retrieved from Uniprot and structure-aligned with AeCXE1 (PDB code: 2O7R) using Expresso [72] and rendered using ESPript3 [73]. The two conserved glycine residues involved in the active site are displayed with a blue star. α, β, n and T represent α-helix, β-strand, 310 helix and β-turn, respectively. (PDF 9.88 kb) [file 12864_2017_3833_MOESM14_ESM.pdf]

Additional file 14.

a

**AtPAE4**	100%											
**AtPAE5**	79.46%	100%										
**AtPAE9**	46.21%	46.44%	100%									
**AtPAE3**	45.66%	46.68%	50%	100%								
**AtPAE6**	43.10%	45.11%	47.61%	77.04%	100%							
**AtPAE1**	39.28%	40.93%	47.80%	56.86%	54.94%	100%						
**AtPAE2**	42.78%	43.79%	47.63%	62.24%	60.15%	66.48%	100%					
**AtPAE12**	44.05%	47.08%	49.36%	61.98%	64.30%	57.69%	68.60%	100%				
**AtPAE10**	44.44%	47.72%	49.24%	62.24%	64.14%	59.89%	69.19%	87.84%	100%			
**AtPAE8**	44.80%	47.73%	48%	52.53%	53.60%	48.62%	53.86%	54.13%	54.66%	100%		
**AtPAE7**	43.20%	46.19%	45.65%	44.02%	45.65%	45.05%	48.64%	46.73%	46.73%	57.33%	100%	
**AtPAE11**	41.57%	44.56%	45.65%	45.92%	44.56%	46.15%	48.91%	48.09%	46.46%	53.80%	83.42%	100%
	**AtPAE4**	**AtPAE5**	**AtPAE9**	**AtPAE3**	**AtPAE6**	**AtPAE1**	**AtPAE2**	**AtPAE12**	**AtPAE10**	**AtPAE8**	**AtPAE7**	**AtPAE11**

b

**AtPAE4**	100%											
**AtPAE5**	89.29%	100%										
**AtPAE9**	61.28%	61.86%	100%									
**AtPAE3**	73.54%	72.37%	68.87%	100%								
**AtPAE6**	71.78%	70.81%	67.31%	90.46%	100%							
**AtPAE1**	70.23%	65.95%	66.53%	82.10%	78.79%	100%						
**AtPAE2**	65.75%	65.36%	58.75%	78.21%	77.43%	74.70%	100%					
**AtPAE12**	71.98%	70.62%	67.31%	85.60%	86.77%	81.71%	80.15%	100%				
**AtPAE10**	72.37%	71.59%	68.28%	85.60%	85.79%	82.49%	80.54%	96.10%	100%			
**AtPAE8**	68.28%	67.89%	73.54%	76.26%	75.29%	76.65%	67.12%	76.65%	76.65%	100%		
**AtPAE7**	69.26%	69.26%	71.40%	75.29%	74.90%	76.26%	65.36%	75.68%	74.90%	84.82%	100%	
**AtPAE11**	67.70%	67.89%	71.40%	75.87%	75.68%	76.84%	65.95%	75.09%	74.12%	85.01%	95.52%	100%
	**AtPAE4**	**AtPAE5**	**AtPAE9**	**AtPAE3**	**AtPAE6**	**AtPAE1**	**AtPAE2**	**AtPAE12**	**AtPAE10**	**AtPAE8**	**AtPAE7**	**AtPAE11**
