# Supplementary material for: Plant pectin acetylesterase structure and function: new insights from bioinformatic analysis
Source: BMC Genomics. 2017 Jun 8;18:456. doi: 10.1186/s12864-017-3833-0 (PMC5465549; doi:10.1186/s12864-017-3833-0)
Supplement: Supplementary file 16 — Primer sequences designed from the cDNA sequence of AtPAE4, AtPAE8 and AtPAE10. (PDF 87 kb) [file 12864_2017_3833_MOESM16_ESM.pdf]

Additional file 16.

PDB Code	Function	Polymer	(%) Sequence identity Modeller	(%) Sequence identity SIAS	(%) Sequence similarity SIAS	Ligand	Reference
4UYU	*Homo sapiens Palmitoleoyl-protein carboxylesterase*	Homo-2-mer	28	29	41	Wnt	Kakugawa *et al.*, 2015
4UZK	*Drosophila Melanogaster Palmitoleoyl-protein carboxylesterase*	Homo-2-mer	24	25	35	Wnt	Kakugawa *et al.*, 2015
3FVR	*Bacillus pumilus Acetyl Xylan Esterase*	Homo-6-mer	18	17	42	/	unpublished
1L7A	*Bacillus subtilis* Cephalosporin C deacetylase	Homo-6-mer	16	17	44	/	unpublished
1VLQ	*Thermotoga maritima Acetyl xylan esterase*	Homo-6-mer	16	15	43	/	Levisson *et al.*, 2012

Wnt: palmitoleoylated protein
The sequence identity and similarity between AtPAE8 and each template was performed with SIAS (http://imed.med.ucm.es/Tools/sias.html).
